# Supplementary material for: ChronoRoot 2.0: an open AI-powered platform for 2D temporal plant phenotyping
Source: Gigascience. 2026 Feb 28;15:giag018. doi: 10.1093/gigascience/giag018 (PMC13042295; doi:10.1093/gigascience/giag018)
Supplement: giag018_Supplemental_File [file giag018_supplemental_file.pdf]

Supplementary Material

S1. Functional PCA

This appendix provides an intuitive explanation of functional principal component analysis (FPCA) for readers without a quantitative background. The goal is to illustrate, through a simple simulated example, how FPCA decomposes variation across a population of curves into independent modes of variation. We consider curves composed of two distinct components: a smooth, broad parabolic shape and a rapid oscillatory pattern (see Figure S1. These components vary independently across samples.

Using a large set of such simulated curves, we apply FPCA to extract the dominant patterns of variation. Each original curve can then be approximately reconstructed as a combination of a mean curve and weighted contributions from the first few principal components. This decomposition helps clarify how variation is structured across a population and which types of patterns dominate.

Fig. S1 shows five example decompositions. Each row corresponds to one simulated curve, split into its two main functional components. This illustration is meant to serve as a visual reference for understanding the role of FPCA in analyzing biological signals that vary smoothly over a continuous domain.

To make this concept more concrete, consider a plant biology scenario where we monitor the growth of plant roots over time. For each plant, we record the length of its primary root at regular intervals, generating a smooth growth curve. These curves reflect dynamic biological processes, including genetic and environmental influences on growth.

Now suppose we are studying several different genotypes or treatments. Each plant's root grows at its own pace and may exhibit unique features: some may grow rapidly early and then plateau, while others grow steadily or even display fluctuating growth due to stress or environmental factors.

By applying FPCA to this dataset of root growth curves, we can:

i. Summarize the dominant patterns of variation: For instance, the first principal component (PC1) might capture differences in overall growth speed (e.g., fast vs. slow growers), while the second component (PC2) might reflect differences in the timing of growth acceleration (e.g., early vs. late spurts).

ii. Reduce dimensionality: Rather than analyzing hundreds of time points, each curve can be represented compactly by just a few scores (weights) corresponding to its projection onto the first few functional components.

iii. Cluster or classify plants based on growth patterns: FPCA scores can be used to group plants with similar dynamic traits or to distinguish between genotypes or treatments based on how their roots grow over time.

This approach is particularly valuable in the plant phenotyping scenarios covered by Chronoroot, where growth dynamics are critical but can be challenging to summarize with static metrics. FPCA allows us to capture and quantify subtle temporal trends in a principled, interpretable way, even when the curves are complex or noisy.

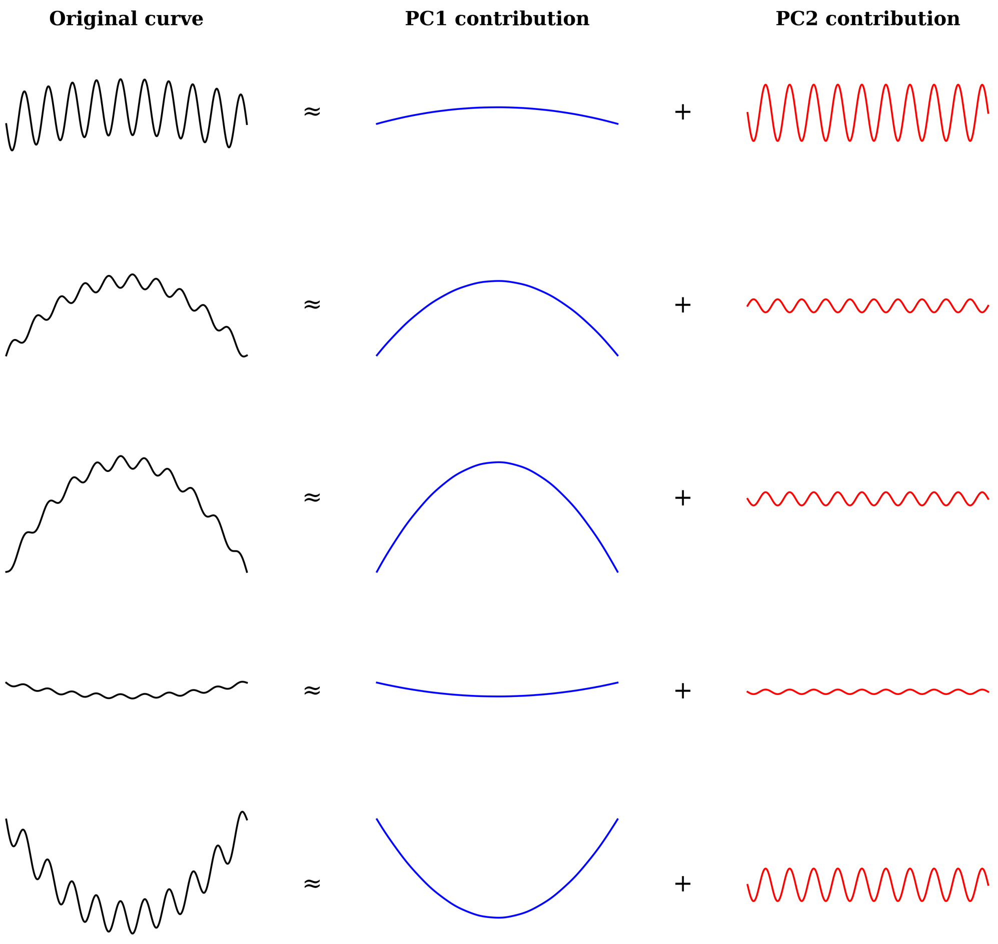

Figure S1. Functional principal component decomposition of simulated curves. Curves were generated as a linear combination of a quadratic function and a high-frequency sine wave, with coefficients drawn independently from normal distributions: $f(x) = a \cdot x^2 + b \cdot \sin(10\pi x)$ with $a \sim \mathcal{N}(0, 1^2)$ and $b \sim \mathcal{N}(0, 0.1^2)$. Each row corresponds to a random instance of such curves. Each curve was centered by subtracting its mean value. Functional PCA was applied to the dataset (using 10,000 randomly sampled curves), and the first two principal components (PCs) were extracted. In each row, the left panel shows the original curve. The middle and right panels show the contributions of the first and second components (PC1 and PC2), respectively. The components are orthogonal and reflect statistically independent sources of variation: the first captures the parabolic shape (due to variation in a), while the second captures the oscillatory pattern (variation in b).

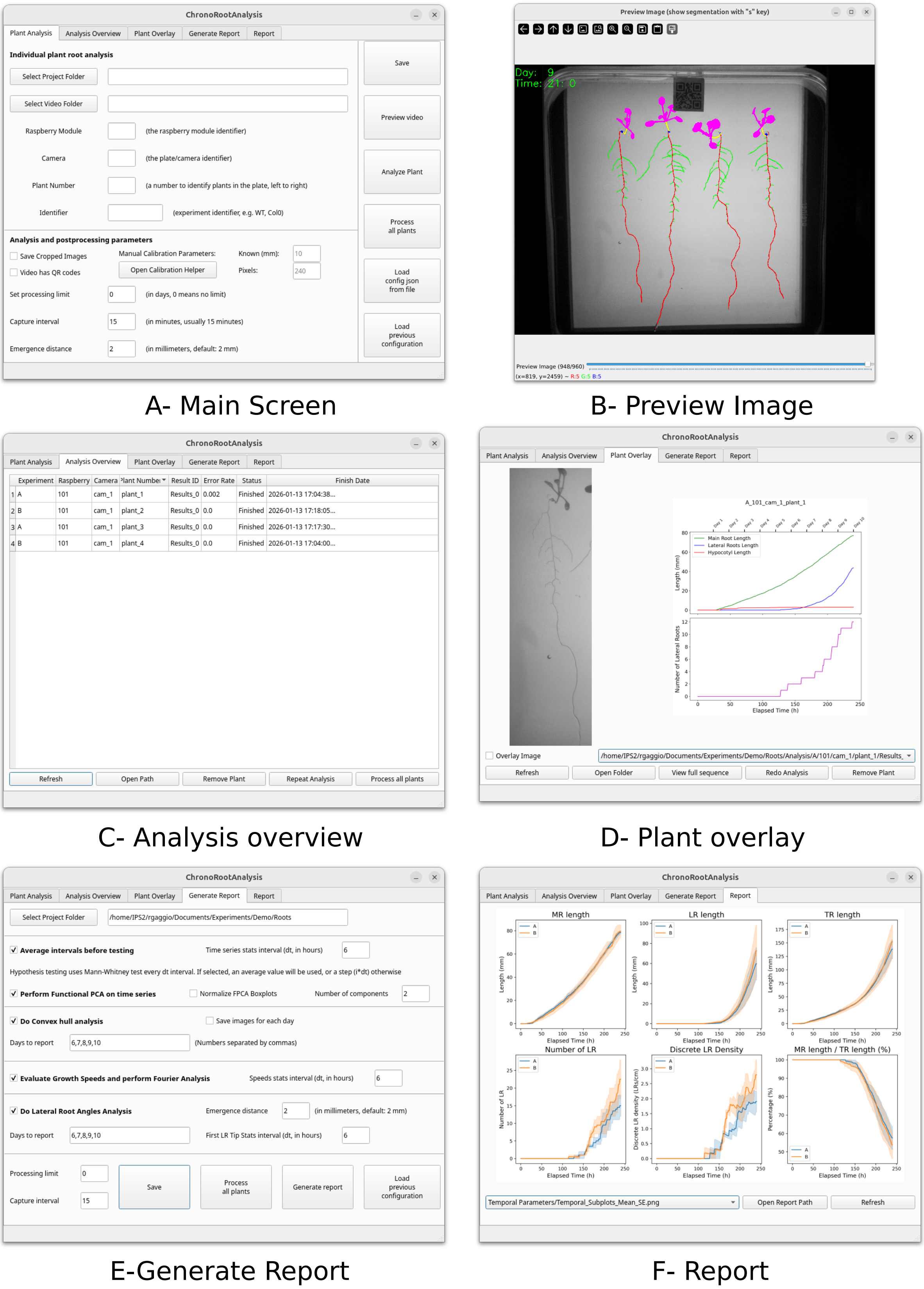

Figure S2. The Standard Root Phenotyping Interface: (A) Main Screen: Plant Analysis tab showing experiment parameters, input/output paths, and processing controls. (B) Preview Image tab with temporal navigation and segmentation toggle for quality assessment. (C) Analysis Overview tab displaying processing completion and error rates. (D) Plant Overlay tab showing individual plant measurements and segmented visualization. (E) Generate Report tab for customizing measurement selection. (F) Report tab displaying finalized architectural analysis results.

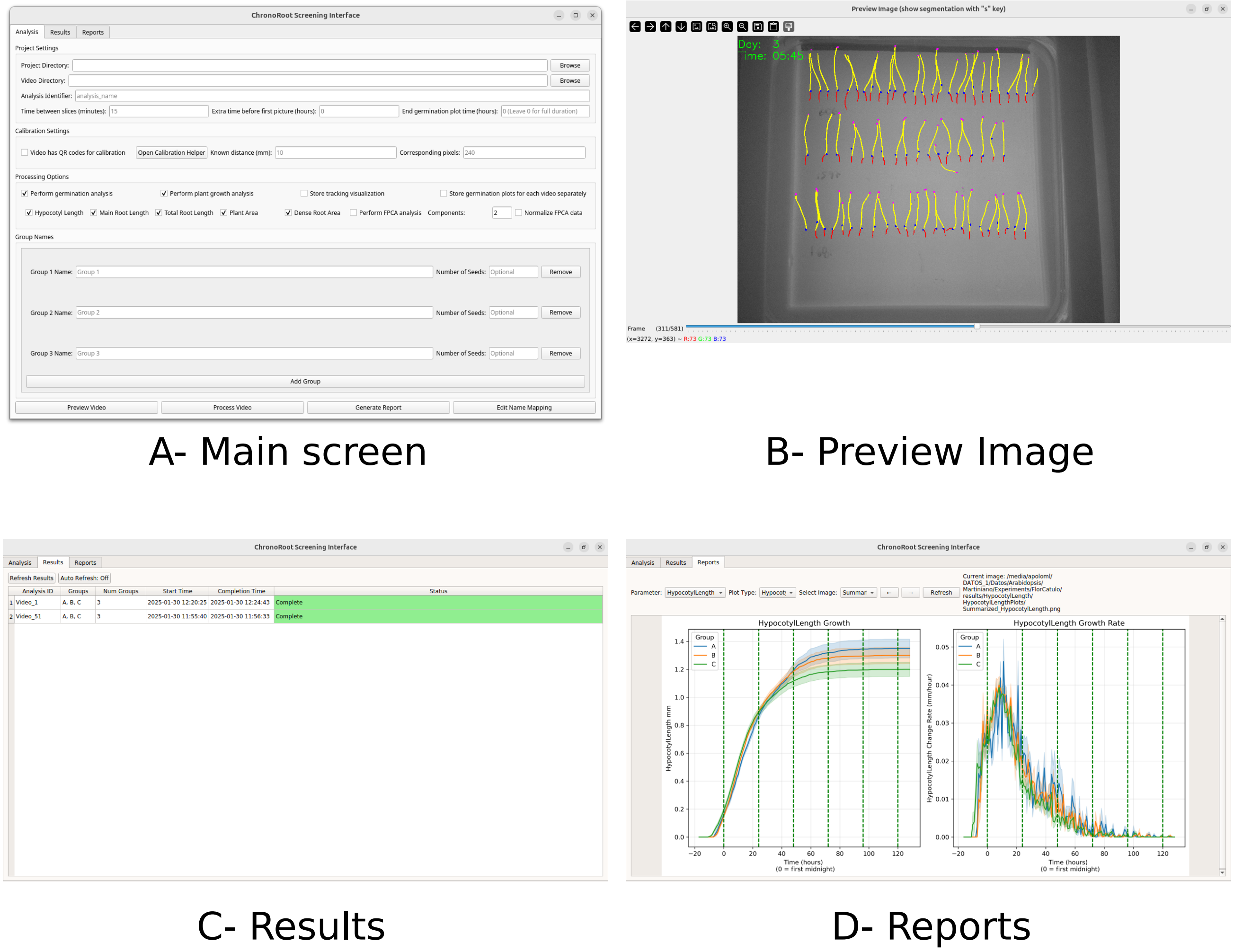

Figure S3. The Screening Interface. (A) Main Screen: Analysis tab with experiment setup, group definition, seed counting, and calibration tools. (B) Preview Image tab providing temporal navigation and segmentation quality assessment. (C) Results tab showing processing status for all plants across experimental groups. (D) Reports tab displaying comparative visualizations and statistical analyses between experimental conditions.

Table S1. Detailed report of segmentation performance across plant organs for *arabidopsis thaliana*.

Configuration	Metric	Complete Root	Main Root	Lateral Root	Seed	Hypocotyl	Leaf	Petiole
Residual	DC	0.800±0.123	0.803±0.122	0.735±0.176	0.686±0.277	0.699±0.230	0.847±0.162	0.758±0.161
	HD	6.658±9.703	6.649±9.895	8.948±11.217	14.811±16.148	10.323±13.220	5.169±7.701	7.160±12.339
	Cp	0.930	0.933	0.895	-	-	-	-
	Cr	0.937	0.939	0.897	-	-	-	-
Residual (Fast)	DC	0.797±0.126	0.800±0.126	0.726±0.183	0.699±0.256	0.695±0.228	0.844±0.163	0.751±0.162
	HD	7.162±10.081	7.243±10.218	9.931±10.178	14.850±15.882	10.051±13.968	5.348±9.297	7.592±13.285
	Cp	0.926	0.929	0.901	-	-	-	-
	Cr	0.935	0.937	0.892	-	-	-	-
Standard	DC	0.798±0.119	0.800±0.119	0.720±0.195	0.696±0.281	0.695±0.240	0.844±0.165	0.758±0.156
	HD	7.765±12.436	7.867±12.513	7.750±9.282	15.792±17.682	8.730±10.412	5.147±7.480	6.666±12.053
	Cp	0.934	0.938	0.890	-	-	-	-
	Cr	0.937	0.938	0.915	-	-	-	-
Standard (Fast)	DC	0.793±0.123	0.796±0.123	0.711±0.203	0.698±0.276	0.687±0.245	0.840±0.166	0.753±0.155
	HD	7.902±12.523	7.986±12.661	8.791±9.679	16.349±18.227	8.916±11.339	7.688±12.846	6.953±11.259
	Cp	0.929	0.933	0.874	-	-	-	-
	Cr	0.936	0.936	0.901	-	-	-	-

Table S2. Detailed report of segmentation performance across plant organs for tomato.

Training	Configuration	Metric	Complete Root	Main Root	Lateral Root	Seed	Hypocotyl	Aerial
Tomato	Standard	DC	0.836±0.178	0.840±0.178	0.643±0.294	0.866±0.202	0.844±0.197	0.778±0.225
		HD	26.470±19.212	26.726±19.700	27.811±19.615	17.744±23.242	7.578±13.767	12.602±16.797
		Cp	0.920±0.133	–	–	–	–	–
		Cr	0.779±0.230	–	–	–	–	–
Tomato	Standard (Fast)	DC	0.805±0.194	0.812±0.188	0.615±0.289	0.884±0.163	0.834±0.201	0.768±0.235
		HD	32.563±19.591	32.483±20.232	33.433±19.507	17.432±22.671	12.784±21.839	19.241±24.454
		Cp	0.910±0.157	–	–	–	–	–
		Cr	0.733±0.253	–	–	–	–	–
Tomato	Residual	DC	0.882±0.154	0.884±0.151	0.651±0.279	0.857±0.191	0.894±0.138	0.807±0.222
		HD	19.676±20.152	15.238±16.119	23.138±20.741	18.005±21.034	6.021±11.986	11.156±17.283
		Cp	0.908±0.153	–	–	–	–	–
		Cr	0.868±0.189	–	–	–	–	–
Tomato	Residual (Fast)	DC	0.871±0.165	0.876±0.159	0.632±0.277	0.839±0.202	0.885±0.147	0.785±0.216
		HD	23.438±20.654	18.147±18.024	24.354±20.547	18.172±20.642	6.290±11.842	13.965±18.417
		Cp	0.904±0.159	–	–	–	–	–
		Cr	0.853±0.197	–	–	–	–	–
Both	Standard	DC	0.853±0.173	0.862±0.162	0.639±0.324	0.885±0.159	0.850±0.186	0.791±0.218
		HD	25.934±20.458	27.324±20.089	23.581±18.204	15.667±21.435	10.579±17.906	15.651±15.909
		Cp	0.914±0.141	–	–	–	–	–
		Cr	0.816±0.223	–	–	–	–	–
Both	Standard (Fast)	DC	0.842±0.181	0.849±0.172	0.638±0.312	0.887±0.150	0.851±0.187	0.771±0.234
		HD	25.137±20.132	26.765±19.903	26.755±18.153	14.647±20.058	8.937±14.857	17.830±17.904
		Cp	0.908±0.156	–	–	–	–	–
		Cr	0.791±0.232	–	–	–	–	–
Both	Residual	DC	0.893±0.162	0.889±0.157	0.673±0.301	0.886±0.166	0.904±0.144	0.854±0.229
		HD	9.943±13.860	11.355±14.100	15.416±16.647	11.604±17.573	5.976±12.757	14.552±23.488
		Cp	0.916±0.138	–	–	–	–	–
		Cr	0.896±0.177	–	–	–	–	–
Both	Residual (Fast)	DC	0.889±0.168	0.883±0.169	0.664±0.288	0.881±0.153	0.895±0.143	0.859±0.196
		HD	10.218±13.685	12.090±13.817	17.317±18.033	12.134±17.901	6.445±13.448	13.454±19.100
		Cp	0.905±0.165	–	–	–	–	–
		Cr	0.899±0.172	–	–	–	–	–

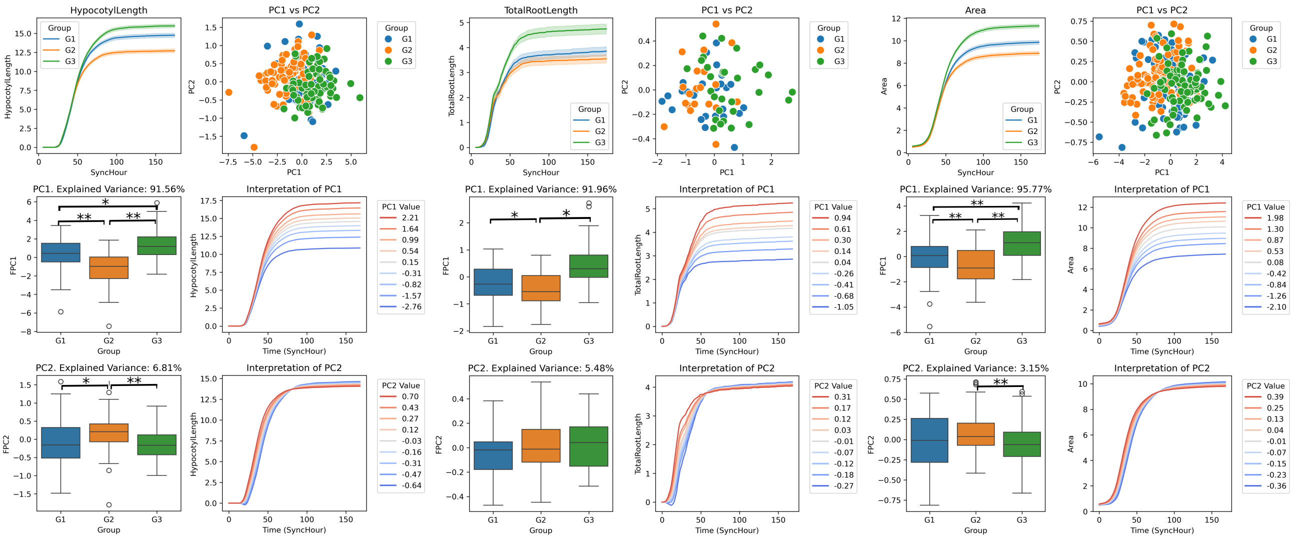

Figure S4. Use Case 3 - FPCA Analysis. To complement the growth dynamics presented in Figure 5-B, we performed FPCA to summarize and compare the developmental trajectories of hypocotyl length, total root length, and projected area across genotypes G1, G2, and G3. The first two principal components accounted for over 90% of the total variance in each trait, capturing the main temporal patterns of growth. For each metric, the top panels show the mean trajectories with standard error bars for each genotype and a scatter plot of PC1 vs PC2. The middle and bottom rows illustrate the distribution of FPCA scores by group for PC1 and PC2, along with visual interpretations of each component. p-values: * < 0.05. ** < 0.001
