# Supplementary material for: ChronoRoot 2.0: an open AI-powered platform for 2D temporal plant phenotyping
Source: Gigascience. 2026 Feb 28;15:giag018. doi: 10.1093/gigascience/giag018 (PMC13042295; doi:10.1093/gigascience/giag018)
Supplement: giag018_GIGA-D-25-00276_revision_1 [file giag018_giga-d-25-00276_revision_1.pdf]

GigaScience

ChronoRoot 2.0: An Open AI-Powered Platform for 2D Temporal Plant Phenotyping

--Manuscript Draft--

**Manuscript Number:**	GIGA-D-25-00276R1
**Full Title:**	ChronoRoot 2.0: An Open AI-Powered Platform for 2D Temporal Plant Phenotyping
**Article Type:**	Technical Note
**Funding Information:**	ECOS-SUD Exchange Program (no. A20N05) — Not applicable<br>IRP LOCOSYM (CNRS) — Not applicable<br>Fondo para la Investigación Científica y Tecnológica — Dr Federico Ariel<br>AXA Research Fund — Dr Federico Ariel<br>International Centre for Genetic Engineering and Biotechnology — Dr Federico Ariel<br>Agence Nationale de la Recherche (ANR-17-EUR-0007) — Dr Thomas Blein
**Abstract:**	Background: Plant developmental plasticity, particularly in root system architecture, is fundamental to understanding adaptability and agricultural sustainability. Existing automated phenotyping solutions face limitations including binary segmentation approaches, restricted structural analysis capabilities, and text-based interfaces that limit accessibility, with most focusing solely on root structures while overlooking valuable information from simultaneous analysis of multiple plant organs.<br>Findings: ChronoRoot 2.0 builds upon established low-cost hardware while significantly enhancing software capabilities and usability. The system employs nnUNet architecture for multi-class segmentation, demonstrating significant accuracy improvements while simultaneously tracking six distinct plant structures encompassing root, shoot, and seed components: main root, lateral roots, seed, hypocotyl, leaves, and petiole. This architecture enables easy retraining and incorporation of additional training data without requiring machine learning expertise. The platform introduces dual specialized graphical interfaces: a Standard Interface for detailed architectural analysis with novel gravitropic response parameters, and a Screening Interface enabling high-throughput analysis of multiple plants through automated tracking. Functional Principal Component Analysis integration enables discovery of novel phenotypic parameters through temporal pattern comparison. We demonstrate multi-species analysis, with Arabidopsis thaliana and Solanum lycopersicum, both morphologically distinct plant species. Three use cases in Arabidopsis thaliana and validation with tomato seedlings demonstrate enhanced capabilities: circadian growth pattern characterization, gravitropic response analysis in transgenic plants, and high-throughput etiolation screening across multiple genotypes.<br>Conclusions: ChronoRoot 2.0 maintains the low-cost, modular hardware advantages of its predecessor while dramatically improving accessibility through intuitive graphical interfaces and expanded analytical capabilities. The open-source platform makes sophisticated temporal plant phenotyping more accessible to researchers without computational expertise.<br>Software availability: https://chronoroot.github.io
**Corresponding Author:**	Nicolas Gaggion<br>Universidad de Buenos Aires Facultad de Ciencias Exactas y Naturales<br>Santa Fe, ARGENTINA
**Corresponding Author Secondary Information:**	
**Corresponding Author's Institution:**	Universidad de Buenos Aires Facultad de Ciencias Exactas y Naturales
**Corresponding Author's Secondary Institution:**	
**First Author:**	Nicolas Gaggion

Powered by Editorial Manager® and ProduXion Manager® from Aries Systems Corporation

**First Author Secondary Information:**	
**Order of Authors:**	Nicolas Gaggion
	Noelia A. Boccardo
	Rodrigo Bonazzola
	Maria Florencia Legascue
	Maria Florencia Mammarella
	Florencia Sol Rodriguez
	Federico Emanuel Aballay
	Florencia Belén Catulo
	Andana Barrios
	Luciano J. Santoro
	Franco Accavallo
	Santiago Nahuel Villarreal
	Leonardo I. Pereyra-Bistrain
	Moussa Benhamed
	Martin Crespi
	Martiniano Maria Ricardi
	Ezequiel Petrillo
	Thomas Blein
	Federico Ariel
	Enzo Ferrante
**Order of Authors Secondary Information:**	
**Response to Reviewers:**	Answer to reviewers for Manuscript GIGA-D-25-00276<br><br>Dear GigaScience Editor,<br><br>We would like to thank you for your positive feedback and the reviewers for their comments. In what follows, we address the reviewer's comment in a point-by-point response. Editorial comments were also addressed in the main manuscript.<br>Reviewer 1<br><br>Comment 1:<br>Reviewer #1: This manuscript presents ChronoRoot 2.0, a platform that significantly enhances the continuous phenotyping of plant root systems and other organs by integrating a multi-class segmentation model based on nnUNet with low-cost hardware. The article is well-structured, methodologically sound, and presents good results, demonstrating strong engineering integration and technical depth. The following suggestions are provided from a practical perspective:<br><br>1. Generalization across species remains unverified<br>Although the authors claim that the model is easily transferable to other plant species, all current experiments are limited to Arabidopsis thaliana. It is recommended to add other results on at least one non-Arabidopsis species to strengthen the claim of cross-species generalizability.<br><br>Response 1:<br><br>To address the concern regarding generalization across species, we have incorporated a comprehensive tomato (Solanum lycopersicum) analysis throughout the manuscript. We present a new dataset of 480 annotated images, detailed in Section 2.3 (Lines

162-182), where we also specify the annotation strategy used for this species. A quantitative evaluation of the nnUNet performance on tomato seedlings is provided in Section 3.1.2 (Lines 568-594) and summarized in Table 4. This evaluation includes a comparison of training strategies, demonstrating that a combined dataset approach enhances performance. Furthermore, we added a new Use Case 4 (Lines 740-763) accompanied by Figure 6, which showcases the practical application of the platform for analyzing tomato phenotypic traits.

Comment 2:
2. Segmentation performance is not clearly presented
The multi-class segmentation results are mainly shown through figures. It would be helpful for reading and understanding to show these results with quantitative metrics such as Dice coefficient and Hausdorff distance for each structure in tabular format.

Response 2:

We have integrated comprehensive quantitative evaluations in tabular format. Tables 3 and 4, replacing original Figure 3, present summarized performance metrics for both Arabidopsis and tomato, while Supplementary Tables S1 and S2 were incorporated to provide detailed per-organ metrics, including Dice coefficients, Hausdorff distance, completeness, and correctness.

Comment 3:
3. Limited system generalizability
The proposed platform strictly relies on a specific hardware-software setup (e.g., infrared image input only), which restricts its applicability to other root imaging datasets. In comparison, tools such as RPT: An integrated root phenotyping toolbox have demonstrated the ability to handle more complex root architectures. Even the authors mention that using model fine-tuning is possible to improve its generalization, however nnUNet model is quite sensitivity to the input data distribution, therefore further evaluation on more diverse and complex datasets is recommended.

Response 3:

We appreciate the reviewer's point about generalizability, and agreed with his statement. To clarify: ChronoRoot 2.0 is specifically designed as the software companion to the ChronoRoot hardware platform (Gaggion et al., GigaScience 2021). The system is optimized for infrared images captured under the controlled conditions of this hardware setup, detailed in lines (183-190). While the nnUNet architecture allows adaptation to other species or imaging systems, this would require retraining with appropriately annotated data from those systems. Importantly, to showcase how our model can be adapted to other species, we have now incorporated results for Tomato plants in the revised manuscript. We incorporated all of this in the Discussion, in lines 782-798.

Comment 4:
4. Software setup remains challenging
Although the authors provide a well-structured code and detailed documentation of the software, the installation process involves numerous dependencies and remains difficult for users without a computational background. It is suggested to release an all-in-one installation package (e.g., a standalone executable) to lower the barrier for broader adoption.

Response 4:

We have completely reworked the software architecture to address this concern, transforming the installation from a complex multi-step process into a streamlined, user-friendly experience that mimics a standalone executable:
We fused the three original Conda environment to a single one to allow a simpler installation for advanced users. More importantly for general users, we used this unified architecture to build a "Standalone-Like" Installer:

Powered by Editorial Manager® and ProduXion Manager® from Aries Systems Corporation

	Apptainer/Singularity Integration: We developed a deployment system using Apptainer that wraps our new Docker base image. Unlike standard Docker containers that can have permission issues, this runs entirely with user-level permissions.<br>Native Launcher Experience: We created installation scripts for both Linux and Windows (at the moment working for Windows 10 via WSL) that automatically generate system-native Start Menu shortcuts.<br>One-Click Execution: Users no longer need to open terminals or activate environments. They simply click the "ChronoRoot" icon in their OS menu, and the software launches instantly.<br>This solution provides the ease of use of a compiled executable while maintaining the scientific reproducibility of a containerized environment. These updates are reflected in the Code and Data Availability section (Lines 873-898), which now includes the updated requirements and a link to the unified container image, and are included in our website at https://chronoroot.github.io/<br>Reviewer 2<br><br>Comment 1:<br>Reviewer #2: In their manuscript entitled "ChronoRoot 2.0: An Open AI-Powered Platform for 2D Temporal Plant Phenotyping", the authors present an updated version of their previously published framework called ChronoRoot, a tool aimed at the segmentation and quantification of morphological features of plants, in particular their root system. New features have been added, the major one being a graphical interface developed to lower the computational barrier that command lines interfaces constitute for researchers without a bioinformatics background. The segmentation performances have been improved using a deep learning method based on the nnUNet architecture, which, according to authors, makes it easier to adapt to other plant species than A. thaliana. The framework can measure a large variety of morphological parameters of plants such as the length of main/lateral roots, the convex hull area, the emergence angle of roots and can also segment and classify up to 6 organs of plants (including leaves). Temporal analysis through Fourier analysis is available, as well as functional principal component analysis (fPCA), that is used by authors to discover hidden features of plants growth. Through three use cases, authors highlight the different functionalities of ChronoRoot 2.0, showing the improvements compared to the first version of the framework.<br>Overall, the manuscript is well written and the logic underlying the study is quite easy to follow. The metrics used and analytical methods are relevant given the objectives of ChronoRoot. However, the installation of the software (through Docker), despite being extensively described by authors in the related GitHub, is far from being easy, and I could not run it correctly even after a few hours of investigations. As a Windows user, I had to adapt the commands and find "tricks" to finally install the software, I guess that a Linux user would have less issues for the installation, but as the tool, with its GUI, is targeted to biologists without a computational background, the use of Docker is already a barrier; so with additional issues coming from a non-UNIX OS, I'm afraid that the target is slightly missed: users able to install ChronoRoot 2.0 are likely the ones with computational background, and then do not need a GUI.<br>While authors provided an extensive documentation for the installation, there are no guidelines or tutorial for the use of ChronoRoot itself; I am then lost when trying to use any of the three components (standard, screening or segmentation tools); there are no indications of the format required for the videos, the filenames, folders architecture, parameters, etc. A step-by-step tutorial with toy data for each components (standard, screening, segmentation) would be more than needed in order to help users learn how to use the framework (or simply check that the installation is correct; currently, I couldn't say if it's not running on my computer because of an incorrect installation or me not knowing how to correctly use the software…)<br>While I recognize the value of Docker for long-term maintenance and version compatibility, I think this still requires basic computational skills that will discourage most of the targeted users. A standalone executable version (ideally three versions for Windows, Linux or Mac) would be really great to have in addition to the Docker solution, so it would make it easier for beginners to adopt ChronoRoot as part of their workflow.<br>In summary, I'm convinced that ChronoRoot 2.0 with its new GUI and improved morphometrics tools, would be a great framework for the plant biologists community, and while I haven't been able to run the software myself, I am confident that it actually performs the features described in the associated manuscript. But I think authors

Powered by Editorial Manager® and ProduXion Manager® from Aries Systems Corporation

should work to improve the accessibility of their tool before the manuscript can be accepted.
The following is a detailed list of my comments, from the major to minor comments:

[MAJOR]

1 - Clear tutorial missing on how to use the software (standard, screening and segmentation components). It would be great to have a doc for each component associated with example data, which users can download in order to reproduce the results shown in the tutorial.

Response 1:

We have addressed this by embedding Demo Data directly into our new container images and also accessible via direct download. By unifying our environment, we were able to package a standardized dataset that is instantly available to every user upon installation. We have added comprehensive, step-by-step tutorials to the new ChronoRoot website (https://chronoroot.github.io/) that utilize this specific data. This ensures that every user, regardless of their operating system, can immediately verify their installation and learn the workflow by reproducing the exact results shown in the documentation, without needing to download external files or configure paths manually.

Comment 2:
2 - A standalone executable version of ChronoRoot 2.0 would be great to have (especially for non-UNIX users).

Response 2:

We have effectively implemented this suggestion by completely re-engineering our Windows support to mimic a standalone executable. We recognized that asking biologists to routinely manage Docker commands or terminals was a significant barrier. To solve this, we performed a complete codebase refactor, unifying our previous multi-environment structure into a single Conda environment, which allowed us to develop a novel Windows Installer (at the moment for Windows 10). This installer bridges the gap between Windows and Linux containers with the following workflow:
One-Time Setup: The user is required to open WSL2 (Windows Subsystem for Linux) only once to run a single installation command (wget and bash installer_windows.sh that is a simple copy/paste in WSL2 terminal). This script handles all complexity: it can either install the lightweight Apptainer runtime, pull our unified container image, and set up the environment, or directly install the conda environment if a installation of Conda is available.
Start Menu Integration: Crucially, the installer automatically generates Windows Desktop and Start Menu shortcuts (.lnk files) that point to the internal Linux container.
Invisible Complexity: Once installed, the user never needs to touch the terminal again. Clicking the "ChronoRoot" icon transparently handles the WSL invocation and container launching in the background.
To the user, ChronoRoot 2.0 now behaves exactly like a native standalone executable: there is no need to type commands, manage Docker daemons, or handle file permissions during daily use. This provides the requested simplicity while preserving the scientific robustness of a containerized environment. These updates are reflected in the Code and Data Availability section (Lines 873-898), which now includes the updated requirements and a link to the unified container image.
Comment 3:
3 - ChronoRoot 2.0, with the nnUNet architecture is said to be easily adaptable to diverse plant species analysis; but authors never showed this claim on other species than A. thaliana. It would be great to test the segmentation and morphometrics measurements on other publicly available datasets with other species than A. thaliana; this would prove the claim and also highlight the interoperability of ChronoRoot (which is not restricted to ChronoRoot hardware setup).

Response 3:

Powered by Editorial Manager® and ProduXion Manager® from Aries Systems Corporation

We have incorporated comprehensive tomato (Solanum lycopersicum) analysis throughout the manuscript (Section 3.1.2 in lines 568-594, Tables 4 and S2, and Use Case 4 in lines 740-763), demonstrating model retraining capability across morphologically distinct species.
However, we wish to clarify an important design consideration: ChronoRoot 2.0 is specifically designed as the software companion to the ChronoRoot hardware platform (Gaggion et al., GigaScience 2021). We do not claim plug-and-play interoperability with arbitrary imaging setups or publicly available datasets from other systems. The system is optimized for infrared images captured under the controlled conditions of this hardware setup.
Testing on external public datasets would require those images to: (1) be captured with similar infrared imaging, (2) have comparable resolution and lighting conditions, (3) show plants in similar growth orientations. Most public root datasets use visible light, different resolutions, or 3D imaging systems, making them incompatible with our trained models without retraining. Our tomato example demonstrates that such retraining is feasible when appropriately annotated data from compatible imaging systems are available, but this is distinct from plug-and-play application to arbitrary external datasets. We incorporated this into the discussion between lines 782-798.

Comment 4:
4 - Similarly, authors compared the segmentation performance of ChronoRoot 2.0 versus its first version; but as other segmentation tools exist, it would be great to compare ChronoRoot 2.0 to the most recent ones, as it's a critical parameter when choosing between available tools.

Response 4:

To improve our segmentation performance analysis, we have expanded the evaluation to include multiple nnUNet architectural variants (Lines 198-208, 513-533). Tables 2-4 and S1-S2 present comprehensive comparisons between the standard nnUNet architecture and the recently introduced nnUNet with residual encoder connections (nnUNet-ResEnc, Isensee et al. 2024), representing the current state-of-the-art in the nnUNet family. Additionally, we evaluate both configurations with and without test-time augmentation (TTA), providing users with multiple accuracy-speed trade-off options.

Regarding comparison with other root segmentation tools: most existing tools (e.g., RootNav, DIRT, SmartRoot) are designed for different imaging modalities (visible light, X-ray CT, or endpoint analysis) or focus solely on binary root/background segmentation. ChronoRoot 2.0 addresses a distinct problem—multi-class segmentation of six plant structures in infrared temporal sequences—making direct quantitative comparison inappropriate. Our comparison with the original ChronoRoot (which addresses the same imaging modality and experimental design) provides the most relevant performance benchmark, while the nnUNet architectural comparison demonstrates we employ current state-of-the-art segmentation methodology.

Comment 5:
5 - I don't understand why users need to manually define the ROI when using the standard interface, while it is automatically detected with the screening interface; similarly, the segmentation methods are different between these two interfaces, the standard one being more accurate than the screening one (as far as I understand the manuscript, since I couldn't test it myself). It would be great to have the choice both for the ROI settings (it could be an automatic detection, and users could then manually edit the ROI if needed) and for the segmentation method (users could prefer using the screening interface with accurate segmentation, even if it's more computationally expensive).

Response 5:
This is an intentional design reflecting fundamentally different experimental scales and objectives. The Standard Interface targets detailed root architecture analysis of individual plants, typically 4-6 plants per plate that can develop for up to two weeks without crossing. Manual ROI definition serves dual purposes: (1) allowing researchers to assign genotype/treatment labels to specific individuals (we are mixing the genotypes inside the plate in random positions), and (2) providing a quality control checkpoint where biologists verify plant identity and suitability before analysis. This

"one ROI equals one plant" approach enables tracking complex root architectures over extended periods where precise individual identity is paramount.
The Screening Interface targets high-throughput experiments with up to 100 plants per plate. At this density, plants can only be measured for a few days before crowding/overlap make individual tracking impossible, and where plant structure is far simpler, allowing an easy automatic detection of the plant. Here, ROIs define groups of plants sharing the same genotype/treatment, not individuals. Within each group ROI, the SORT algorithm automatically detects and tracks multiple plants. This design reflects that high-density screening sacrifices long-term individual tracking for increased sample size during the brief early developmental window before crowding.
Both interfaces use identical nnUNet segmentation at their core—there is no "more accurate" versus "less accurate" segmentation method. The fundamental difference is analytical scope: Standard Interface performs deep architectural analysis of few individuals over weeks, while Screening Interface performs rapid comparative analysis of many individuals over days.
We incorporated this in Discussion lines 799-810
Comment 6:
[MINOR]
1 - (page 4; section "Specialized Processing Pipelines") "This approach effectively addresses common imaging challenges in plant phenotyping, such as water droplets, condensation artifacts, or temporary occlusions, providing stable root structure detection throughout developmental timeframes.": a reference is missing.
In a similar frame, how does nnUNet perform against imaging artefacts?

Response 6:

Regarding the performance of nnU-Net against imaging artifacts, it is important to note that while nnU-Net's default data augmentation (e.g., Gaussian noise, blurring, and simulated intensity shifts) provides a baseline level of robustness, it is not inherently designed to recognize domain-specific artifacts like water droplets or agar scars as "background."

Our strategy addressed this through an iterative approach. Each time we encounter "failure case" images where scars in the agar, droplets, condensation or possible light reflections initially caused segmentation errors, we manually correct them, and re-integrate them into the training dataset. Empirically, we found that incorporating these specific artifacts directly into the training set led to a significant increase in performance. Because our hardware maintains a fixed light source and camera geometry, these artifacts (droplets and agar disturbances) are the primary sources of noise; by training nnU-Net to explicitly classify them as background, the model achieves high stability across developmental timeframes.

We plan to continue this strategy in the future, evolving our training set and model weights.

Comment 7:
2 - What is the minimal detection length for lateral roots (as I guess it depends on the skeletonization process and related noise on main root)?

Response 7:
In our pipeline, the spatial resolution is approximately 0.04~0.05 mm/pixel. To mitigate skeletonization noise and ensure that spurs on the root axes are not incorrectly identified, we implement a structural pruning threshold on the skeleton prior to graph construction. This threshold is set to a minimum of 5 pixels, which establishes a theoretical minimal detection length of 0.2 mm.
Furthermore, as now described in the manuscript in lines 340-355, we also apply a temporal verification window during measurement post-processing. Any measurement, including root detections, is only validated if the structure persists for at least 6 hours, effectively filtering out transient artifacts or temporary occlusions. These combined spatial and temporal filters ensure biological consistency for all tracked organs.

Comment 8:
3 - The measurements results that can be exported in RSML format is a nice feature for interoperability, great job!

Powered by Editorial Manager® and ProduXion Manager® from Aries Systems Corporation

Response 8:

Thank you!

Comment 9:
4 - (page 6; section "Software Implementation and User Interface") "The interface includes
quality control through visual feedback systems": I don't understand the meaning of this sentence.
Response 9:
We have revised this section of the manuscript to clarify that "visual feedback" refers to the interactive validation steps built into the software workflow. Users can first inspect the segmentation quality on the raw video before selecting individual plants and defining their specific root starting positions. After the automated graph construction, the interface allows the user to watch the growth video with the resulting skeletonized graph and measurements overlaid in real time. This allows for a direct visual inspection of the tracking performance, ensuring that any plant with biological or technical anomalies can be discarded or re-analyzed before being included in the final automated reports and statistical investigations.
This was incorporated in the tutorials and in lines 445-453 in the manuscript

Comment 10:
5 - (page 5; section "Multiple Plant Screening Analysis") "The hypocotyl analysis module incorporates validation steps for reliable measurement in multi-plant scenarios. The system automatically detects physiologically impossible growth rates and artifacts that can occur when different plants touch and their segmentations combine. Biological constraints are enforced through validation of non-decreasing length measurements and growth directions against expected gravitropic responses."
This feature is an interesting QC check, but I was wondering if user can disable it. I couldn't test it, so the following is based on conjectures: what is the threshold(s) for the impossible growth rates? Can user edit this threshold(s)? For the gravitropic responses, does the analysis assume gravity is directed to the bottom of images? I'm thinking about a very particular experimental case, but given the nature of the ChronoRoot hardware, I think this would be the typical setup for nomad experiments, especially for experiments in low gravity (in the ISS for example). In this particular case, I guess that the gravitropic responses could be studied and users would like to define the gravity field (or no gravity field). For this reason, I think it would be nice to enable users to decide if they want the QC check or not. Again, this is a very particular case, but it illustrates how a validation steps can limit the range of applications of a given setup.

Response 10:

We thank the reviewer for this observation. We agree that hard-coded assumptions about growth direction could limit the tool's applicability in novel setups, such as the microgravity or inverted experiments mentioned. In response, we have updated the analysis module to remove the directional gravitropic constraint, without finding any problems in the reproduction of our results.

Still, this validation step serves as a high-constraint filter primarily designed to detect and handle plant overlaps. When seedlings touch, segmentation algorithms often merge them, resulting in sudden, non-biological jumps in measured size. To prevent these artifacts from corrupting the dataset, the system automatically identifies and removes these compromised traces from the processed results. To address the reviewer's suggestion regarding flexibility without adding complex UI toggles, we explicitly rely on data transparency. Since the processed output prioritizes statistical purity, we ensure that researchers always have access to the raw, unfiltered measurements. This allows users to bypass our collision filtering entirely and apply custom validation logic if their specific experiment requires analyzing touching plants or unusual growth patterns. This was incorporated to the manuscript in lines 376-381.

Comment 11:
6 - I think some figures would look better in log scale: Figures 3B-3C, spectra in Figure

Powered by Editorial Manager® and ProduXion Manager® from Aries Systems Corporation

4, germination curves in Figure 6

Response 11:

Figures 3B-3C were changed to tables 2, 3 and 4 according to reviewer's comments to help the readability.
As for the germination curves, we removed the later time point where no change was visible, increasing the graph time-resolution where the majority of the changes are happening. We believe this makes the current version without log transformation already more readable.

Comment 12:
7 - In Figure 4's legend, the number of replicates per conditions is missing; it is an important metric as mean curves and standard deviations are used.

Response 12:

Figures 3, 4 and 5 captions now include the number of replicates per condition.

Comment 13:
8 - Figure 4D: why some negative values for LR length?

Response 13:

Figure 4D (now 3D in the revised manuscript) is intended to show how the curves vary when we modify the different values of the Principal Components, for interpretability purposes. As we vary these components to extreme values, non plausible solutions emerge. As you can see, the negative values in the explanations correspond to curves with very high (PC2) or very low (PC1) values.

Comment 14:
9 - For the FFT analysis and circadian cycles, it would be great to conduct the analysis on plants keeping in dark: how does it translate to the power spectrum?

Response 14:

This is a very interesting suggestion. However, while the question is relevant, our paper focuses on presenting a case study analysis of ChronoRoot's enhancements, rather than conducting new experiments to address additional biological questions. That is the reason why we believe that such an analysis is out of the scope of our current work, and would be more appropriately explored in a dedicated paper examining the regulation of root architecture by light regimes.

Comment 15:
10 - Some statistical significance tests are missing:
* difference between spectra and significance of peaks (Figure 4C)
* Figures 5B & S4

Response 15:

This was incorporated in the figures.

Comment 16:
11 - I would like to see the graphs PC1 vs. PC2 for the fPCA analyses; it's always informative to have a visual overview of the PCA projections (maybe as supplementary figures if it doesn't fit the main ones?).

Response 16:

We thank the reviewer for the idea. It is now included in Use Cases 1 (Figure 2), 3 (Figure S4) and 4 (Figure 6) .

Powered by Editorial Manager® and ProduXion Manager® from Aries Systems Corporation

Comment 17:
12 - It might be over my head, but I have the feeling that the fPCA is missing important correlations between morphological metrics (especially MR vs. LR lengths). It is likely due to the fact that the fPCA is conducted on a projection of the multi-dimensional dataset [MR(t), LR(t), TR(t), etc.]_i to one of its dimensions (for example MR(t)); hence losing the temporal correlations between this measurement and others. I was wondering to what extent it would be feasible to integrate all morphological metrics for a given plant to a single fPCA? I would be interested in authors' thoughts on this.

Response 17:
We thank the reviewer for this insightful observation. Indeed, the analysis currently presented in the manuscript performs functional PCA independently for each morphological metric (e.g., MR, LR, TR), and therefore does not explicitly model cross-metric temporal correlations.
From a methodological standpoint, the reviewer is correct that a multivariate extension of fPCA (often referred to as multivariate or vector-valued fPCA) could, in principle, integrate several time-varying morphological descriptors into a single joint decomposition. However, this approach would add significant methodological complexity, require careful balancing between traits, and lead to components that are much harder to interpret biologically than the univariate fPCA used here.
Our primary objective in this work is to provide a robust, interpretable, and broadly accessible framework for phenotypic time-series analysis within ChronoRoot. The current per-trait fPCA design was chosen deliberately to maximize interpretability and usability for non-expert users, while still capturing the dominant temporal modes of variation for each biologically meaningful metric. Implementing a full multivariate fPCA pipeline would significantly increase the methodological burden of the tool and shift it away from this design philosophy.
We therefore consider multivariate fPCA to be a valuable direction for advanced users who wish to perform more specialized integrative analyses, and we provide access to all raw temporal measurements so that such analyses can be carried out externally using dedicated statistical frameworks.

Comment 18:
13 - Some typos:
* Use case 1 paragraph refers to Figure 5, while it's actually Figure 4
* User case 3 paragraph mentions Figure 5, while it should be Figure 6
* In figure 4, color legends have been switched
* In use case 2 "construct bearing 2000-pb region": should be "2000 bp"

Response 18:

We have fixed all these typos.
Reviewer 3
Comment 1:
This paper presents a new machine learning and software pipeline for temporal analysis of arabidopsis seedlings. The work improves upon prior work by the same authors on Chronoroot, and shows good performance. Notably, the machine learning pipelines, measurement / analysis and user experience are substantially improved.

I have no major concerns over this paper, I think it is very good. The use cases did a good job of showing the benefits of the software, and the figures are of very high quality. The work outputs standardized file formats and will be a welcome addition to the community work in this area. The github repository hosting the software is well put together, with excellent instructions and guidance on installation and use. I have some minor suggestions for the manuscript:

- It isn't clear what resolution of images are being used during the image processing and machine learning pipeline. The native resolution of the images is quoted. It may be that nnUnet handles this, but it is useful information to include. In general the detail on the training of the networks is fairly vague, again perhaps nnUnet handles this but these parameters remain useful should someone wish to improve upon the network. Is tiling used, resizing? What about training vs inference?

Powered by Editorial Manager® and ProduXion Manager® from Aries Systems Corporation

Response 1:

We thank the reviewer for this observation. Images are captured at native 3280 x 2464 resolution (approximately 0.04 mm per pixel) and processed by nnUNet at full single-channel resolution without resizing. This is now detailed in Lines 184-189.

The nnUNet framework self-configures its architecture based on dataset properties, automatically determining optimal patch sizes, that is the full image in our case, network depth, kernel sizes, stride patterns, normalization schemes, and learning rate schedules during the planning phase prior to training. This self-configuration eliminates the need for manual hyperparameter tuning.

Comment 2:
- Related to this, there is a throwaway line in the discussion that it is easy to retrain to new species because of the use of nnUnet. More info here is required to make this claim, presumably you are not specifically facilitating this via the software? Training nnunet doesn't necessarily mean that the output will be compatible with your measurement pipeline, depending on the size and shape of the species? Your manuscript submission includes a fork of the nnUnet repo - what changed in your version, should people training use the original or your copy? Perhaps a more straightforward question would be, would re-training of the network require someone with a strong technical background?

Response 2:

We apologize for the confusion regarding the nnUNet repository reference. During initial development of ChronoRoot 2.0, we worked with nnUNet version 1.x, which required minor modifications for integration. ChronoRoot 2.0 now uses the official nnUNet v2 repository (https://github.com/MIC-DKFZ/nnUNet) without any modifications to the framework itself. The improved modularity of nnUNet v2 eliminated the need for custom changes.

Regarding retraining for new species: the process requires modest technical skills but no machine learning expertise. The workflow consists of: (1) manually annotating a set of images (we recommend 300-500 images minimum) using ITK-SNAP with the same five or six-class scheme (main root, lateral roots, seed, hypocotyl, aerial (or leaves and petiole), (2) organizing images and annotations following nnUNet's standardized folder structure, (3) executing standard nnUNet preprocessing and training commands, and (4) copying the trained weights to the ChronoRoot model directory. nnUNet automatically handles architecture configuration, hyperparameter optimization, and cross-validation, where no manual tuning is required. A researcher comfortable with command-line operations and basic file management can complete this workflow by following our documentation. As for step 2, probably the most arbitrary one, we provided scripts to convert from our ground truth data to the nnUNet format. We are working on improving our documentation for this particular case, which can be found in our dedicated website at https://chronoroot.github.io/tutorials/training_on_your_images/.

To better illustrate how our model can be adapted to new species, we are now including experiments in Tomato species (see lines 162-182, 568-594), where the model has been retrained and repurposed for this plant.

Comment 3:
- It might be useful to extend your consideration of the limitations of the work - for example the age, species etc. of the seedlings you expect to work here. Other software in this space often struggles with well-established root systems with many crossovers, you mention this briefly.

Response 3:

We thank the reviewer for this suggestion. Key constraints include:

Powered by Editorial Manager® and ProduXion Manager® from Aries Systems Corporation

Temporal limitations: ChronoRoot hardware allows the imaging in classical 12cmx12cm square plates, and a lot of limitations come from this restricted space. Experiments typically conclude when the main root reaches the plate bottom (usually 10-14 days for Arabidopsis, 5-7 days for tomato), as roots may then grow along the bottom surface (invisible to imaging) or cease elongation and increase lateral root production. To ensure comparable developmental stages, researchers select analysis timeframes after reviewing complete image sequences.

Plant density and crowding:
The Standard Interface supports 4-6 plants per plate for extended tracking, as higher densities lead to root system overlap that prevents individual architectural analysis. The Screening Interface accommodates up to 100 plants per plate but limits analysis to early development (3-5 days) before crowding occurs. When plants physically touch, segmentation cannot reliably separate individuals.

Species and developmental stage: The system is optimized for seedling-stage analysis when organs are clearly distinguishable. Mature plants with dense root systems featuring numerous crossovers and overlapping lateral roots present challenges for accurate segmentation and tracking. Other species with very different morphologies from our training data (Arabidopsis and tomato) will require retraining with appropriately annotated data.

We included this in Discussions line 798-809

Reviewer 4

Comment 1:
Reproducibility report for: ChronoRoot 2.0: An Open AI-Powered Platform for 2D Temporal Plant Phenotyping

- Recommendations for authors
We recommend the following improvements based on our observations to enhance the study's reproducibility:

-- ROI selection and seed placement: The results depend heavily on ROI selection and seed placement. However, the tutorial did not mention how to choose this to reproduce the end result. Adding this would improve reproducibility.

Response 1:
The reviewer is correct that the manual initialization step is the source of the numerical variation.
In the Standard Interface, the "Seed Placement" does not merely indicate the location of the seed; it defines the spatiotemporal origin of the main root's growth. To ensure accurate quantification, the user must advance the video timeline to the specific frame where the main root begins to emerge and segment, and select the point at that moment.
If the point is selected at the beginning of the video (Day 0) before emergence, the algorithm may integrate slight segmentation noise or seed coat artifacts during the dormancy period, leading to the differences in length and p-values observed by the reviewer. When the point is selected at the precise moment of emergence, the noise above this point is effectively filtered.
Tutorials for the interface now include many examples and explanations on how to place the initial point, for both tomato and arabidopsis.
Available at our new website: https://chronoroot.github.io/
Comment 2:
-- MacOS installation guide: An installation guide is provided for Linux and Windows. Adding a guide for MacOS users would enhance reproducibility.

Response 2:

Powered by Editorial Manager® and ProduXion Manager® from Aries Systems Corporation

	We included a small section on our new docker tutorial at https://chronoroot.github.io/tutorials/docker/. We acknowledge that our support is mostly Linux and Windows 10 at the moment, and we will continue working on better support.<br><br>Comment 3:<br>-- p-value reporting: The p-value reported in Figs. 4B and 4D is currently only through an asterisk, indicating significance. Reporting exact values improves reproducibility.<br><br>Response 3:<br><br>We appreciate the reviewer's focus on reproducibility. To clarify, the figures presented in the use cases are direct exports from the ChronoRoot 2.0 interface, designed to showcase the platform's automated visualization and reporting capabilities. We have now standardized the notation across all figures, using * for $p<0.05$ and ** for $p<0.001$. Regarding the reporting of exact values: because these use cases are functional demonstrations of the software's output, we chose to maintain the visual format a user would see in the software's dashboard. However, we have added a clarification to the manuscript stating that for every analysis, ChronoRoot 2.0 automatically generates text files containing the exact p-values, mean, standard deviation, and sample sizes (n) for every parameter. This ensures that while the visual output is optimized for quick interpretation, the raw numerical data is always preserved for rigorous verification. We have updated the text in lines 607–614 to make this explicit.
**Additional Information:**	
**Question**	**Response**
Are you submitting this manuscript to a special series or article collection?	No
**Experimental design and statistics**<br><br>Full details of the experimental design and statistical methods used should be given in the Methods section, as detailed in our Minimum Standards Reporting Checklist. Information essential to interpreting the data presented should be made available in the figure legends.<br><br>Have you included all the information requested in your manuscript?	Yes
**Resources**<br><br>A description of all resources used, including antibodies, cell lines, animals and software tools, with enough information to allow them to be uniquely identified, should be included in the Methods section. Authors are strongly encouraged to cite Research Resource Identifiers (RRIDs) for antibodies, model organisms and tools, where possible.	Yes

Have you included the information requested as detailed in our Minimum Standards Reporting Checklist?	
**Availability of data and materials**<br><br>All datasets and code on which the conclusions of the paper rely must be either included in your submission or deposited in publicly available repositories (where available and ethically appropriate), referencing such data using a unique identifier in the references and in the “Availability of Data and Materials” section of your manuscript.<br><br>Have you have met the above requirement as detailed in our Minimum Standards Reporting Checklist?	Yes
GigaScience has policies and guidelines in place for the use of generative AI-writing tools such as ChatGPT. If you have used such writing tools to assist with writing the manuscript this must be declared and cited in the text. Authors should not list AI-writing tools and other AI-assisted technologies as an author or co-author and should acknowledge that they are fully responsible for text generated or refined by AI-writing tools.<p><br><br>A summary of use (particularly in the introduction or among methods) needs to be included at the end of the paper, and the outputs should also be included as a supplementary file hosted in GigaDB or other open repositories. Please read our guidelines for more information. <p><br><br>By submitting to GigaScience, you are aware of the journal's AI-writing tools policy, and if you have declared use of such tools below, you have acknowledged this where appropriate in your manuscript and have made a summary of use and outputs available. <p><br><b>AI-assisted writing tools have been used in the preparation of this manuscript?	No

Manuscript clean
Click here to access/download;Manuscript;Manuscript.pdf

GigaScience, 2026, 1–19

doi: xx.xxxx/xxxx
Manuscript in Preparation
Technical Note

TECHNICAL NOTE

ChronoRoot 2.0: An Open AI-Powered Platform for 2D Temporal Plant Phenotyping

Nicolás Gaggion[1,2,3,4,5,*], Noelia A. Boccardo[1,2], Rodrigo Bonazzola[6], María Florencia Legascue[1,2], María Florencia Mammarella[2], Florencia Sol Rodriguez[1], Federico Emanuel Aballay[1], Florencia Belén Catulo[1], Andana Barrios[4,5], Luciano J. Santoro[1], Franco Accavallo[2], Santiago Nahuel Villarreal[1,2], Leonardo I. Pereyra-Bistrain[4,5], Moussa Benhamed[4,5], Martin Crespi[4,5], Martiniano María Ricardi[1], Ezequiel Petrillo[1], Thomas Blein[4,5], Federico Ariel[1] and Enzo Ferrante[3]

[1]Instituto de Fisiología, Biología Molecular y Neurociencias (IFIBYNE), CONICET-Universidad de Buenos Aires, Argentina and [2]APOLO Biotech, Argentina and [3]Instituto de Ciencias de la Computación, CONICET-Universidad de Buenos Aires, Argentina and [4]Université Paris-Saclay, CNRS, INRAE, Université Evry, Institute of Plant Sciences Paris-Saclay (IPS2), 91190 Gif-sur-Yvette, France and [5]Université Paris Cité, CNRS, INRAE, Institute of Plant Sciences Paris-Saclay (IPS2), 91190 Gif-sur-Yvette, France and [6]Instituto de Investigación en Señales, Sistemas e Inteligencia Computacional sinc(i), CONICET-Universidad Nacional del Litoral, Argentina

[*]Address correspondence to: ngaggion@dc.uba.ar

Abstract

Background: Plant developmental plasticity, particularly in root system architecture, is fundamental to understanding adaptability and agricultural sustainability. Existing automated phenotyping solutions face limitations including binary segmentation approaches, restricted structural analysis capabilities, and text-based interfaces that limit accessibility, with most focusing solely on root structures while overlooking valuable information from simultaneous analysis of multiple plant organs.
Findings: ChronoRoot 2.0 builds upon established low-cost hardware while significantly enhancing software capabilities and usability. The system employs nnUNet architecture for multi-class segmentation, demonstrating significant accuracy improvements while simultaneously tracking six distinct plant structures encompassing root, shoot, and seed components: main root, lateral roots, seed, hypocotyl, leaves, and petiole. This architecture enables easy retraining and incorporation of additional training data without requiring machine learning expertise. The platform introduces dual specialized graphical interfaces: a Standard Interface for detailed architectural analysis with novel gravitropic response parameters, and a Screening Interface enabling high-throughput analysis of multiple plants through automated tracking. Functional Principal Component Analysis integration enables discovery of novel phenotypic parameters through temporal pattern comparison. We demonstrate multi-species analysis, with *Arabidopsis thaliana* and *Solanum lycopersicum*, both morphologically distinct plant species. Three use cases in *Arabidopsis thaliana* and validation with tomato seedlings demonstrate enhanced capabilities: circadian growth pattern characterization, gravitropic response analysis in transgenic plants, and high-throughput etiolation screening across multiple genotypes.
Conclusions: ChronoRoot 2.0 maintains the low-cost, modular hardware advantages of its predecessor while dramatically improving accessibility through intuitive graphical interfaces and expanded analytical capabilities. The open-source platform makes sophisticated temporal plant phenotyping more accessible to researchers without computational expertise.
Software availability: https://chronoroot.github.io

Key words: Plant phenotyping; Root system architecture; Deep learning segmentation; Temporal analysis; High-throughput screening; Open-source software; *Arabidopsis thaliana*; Tomato

Introduction

Plants, as sessile organisms, must develop sophisticated adaptive strategies to cope with their immediate environment throughout their lifecycle. This fundamental biological constraint has driven the evolution of remarkable developmental plasticity, enabling plants to complete their life cycles under varying and often suboptimal growth conditions [1]. The root system, being the primary interface between plant and soil, exhibits particularly notable phenotypic plasticity in response to environmental variables [2]. Understanding the dynamics of root system development and its plastic responses has become increasingly critical in the context of climate change and the growing need for sustainable agriculture.

Under controlled conditions, root development is typically observed through images of plants growing vertically on semisolid agarized medium. Root system architecture (RSA) is then characterized through various parameters such as main root length and lateral root density [3]. Several semi-automatic tools assist in root phenotyping at specific time points, yet comprehensive temporal analysis remains technologically challenging [4, 5]. In addition, most existing tools focus exclusively on root structures, overlooking valuable information that could be gained from analyzing other plant organs simultaneously, from an integrative perspective.

The original ChronoRoot [6] system introduced automated temporal phenotyping through a low-cost approach combining off-the-shelf electronics, 3D printed hardware components, and deep learning models. This system demonstrated the potential for automated analysis in plant root developmental studies through high-throughput temporal phenotyping of *Arabidopsis thaliana* RSA. However, its practical application revealed several limitations that restricted its broader adoption in the plant science community.

The binary segmentation approach of the original Chronoroot, while effective for basic root architecture analysis, proved inadequate for capturing the full complexity of plant development, particularly during early growth stages. The requirement for manual seed positioning created a bottleneck in high-throughput analysis, while the text-based interface presented a barrier to adoption by researchers without computational expertise. Additionally, the system's focus on root structures alone meant that valuable information about other plant organs and their developmental relationships was not captured. These limitations highlighted the need for a more comprehensive and accessible solution that could capture the full complexity of plant development while maintaining analytical rigor.

ChronoRoot 2.0: An Integrated Solution for Plant Phenotyping

Building upon this foundation, we present ChronoRoot 2.0, which significantly expands the capabilities and accessibility of the platform through several key innovations, as illustrated in the comprehensive pipeline shown in Figure 1. The complete workflow begins with the Hardware Module (Fig. 1-A) that combines Raspberry Pi-controlled cameras and infrared LED backlighting. This setup enables consistent Image Acquisition (Fig. 1-B) irrespective of day/night cycles, with a temporal resolution of 15 minutes for extended monitoring of plant development.

At the core of these improvements is an upgraded nnUNet Segmentation module (Fig. 1-C) based on the nnUNet architecture [7], which performs simultaneous multi-class segmentation of six distinct plant structures: main root (class 1), lateral roots (class 2), seed (class 3), hypocotyl (class 4), leaves (class 5) and petiole (class 6). This advancement enables comprehensive tracking of plant development from seed to mature seedling, capturing the intricate relationships between different organs during growth. The choice of nnUNet as the core architecture was motivated by its proven success in medical image segmentation and its ability to self-configure hyperparameters, making it particularly suitable for biologists without extensive machine learning expertise. While the segmentation model is trained to work with both *Arabidopsis thaliana* and *Solanum lycopersicum* (tomato), it can be easily adapted for other species, leveraging the self-configuring capabilities of the nnUNet architecture. This architectural choice, combined with newly developed graphical user interfaces, significantly lowers the barrier to entry for researchers seeking to implement plant phenotyping in their work.

After segmentation, researchers can select between two distinct but complementary interfaces based on their experimental needs. The Standard Root Phenotyping Interface (Fig. 1-D) maintains continuity with the original ChronoRoot design, focusing on detailed RSA analysis of individual plants. This interface provides researchers with tools for precise measurement and analysis of root development patterns through a graph-based representation approach. It maintains the core strengths of the original ChronoRoot system while adding comprehensive visualization capabilities and an intuitive graphical user interface for analyzing basic architecture, growth dynamics, spatial distribution, and newly determined angular measurements.

The new Screening Interface (Fig. 1-E) extends the system's capabilities by enabling automated analysis of multiple plants simultaneously. It incorporates the Simple Online Realtime Tracking (SORT) algorithm[8] for robust plant identification across experimental groups, along with manual calibration tools for standardized measurements. This interface specializes in early development analysis through three dedicated modules: germination analysis, hypocotyl analysis, and plant analysis, enabling researchers to efficiently process and compare multiple experimental conditions.

Both interfaces implement comprehensive quality control mechanisms through real-time feedback via interactive visualization tools. The system introduces several novel analytical capabilities, including automated seed detection and tracking that eliminates the need for manual positioning. Remarkably, we have incorporated here Functional Principal Component Analysis (FPCA) for time series comparison across different groups of plants (e.g. genotypes, treatments, or combinations), enabling the discovery of new data-driven phenotypic parameters that may not be apparent through traditional analysis methods.

Through this dual-interface approach, ChronoRoot 2.0 addresses diverse needs of the plant science community. The Standard Interface provides the precise control and detailed analysis capabilities needed for in-depth root architecture studies of individual plants, while the Screening Interface enables efficient processing of multiple plants and experimental groups, making high-throughput phenotyping accessible to a broader research community.

The remainder of this paper details the technical implementations and validations of each component of Chronoroot 2.0. We begin by describing the enhanced segmentation capabilities and their validation, followed by detailed explanations of the specialized analyses enabled by each interface. We then present the statistical frameworks implemented for both detailed RSA studies and multi-plant screening experiments. Finally, we discuss the system's graphical user interfaces and their role in making advanced phenotyping accessible to the broader plant science community.

Compiled on: January 17, 2026.
Draft manuscript prepared by the author.

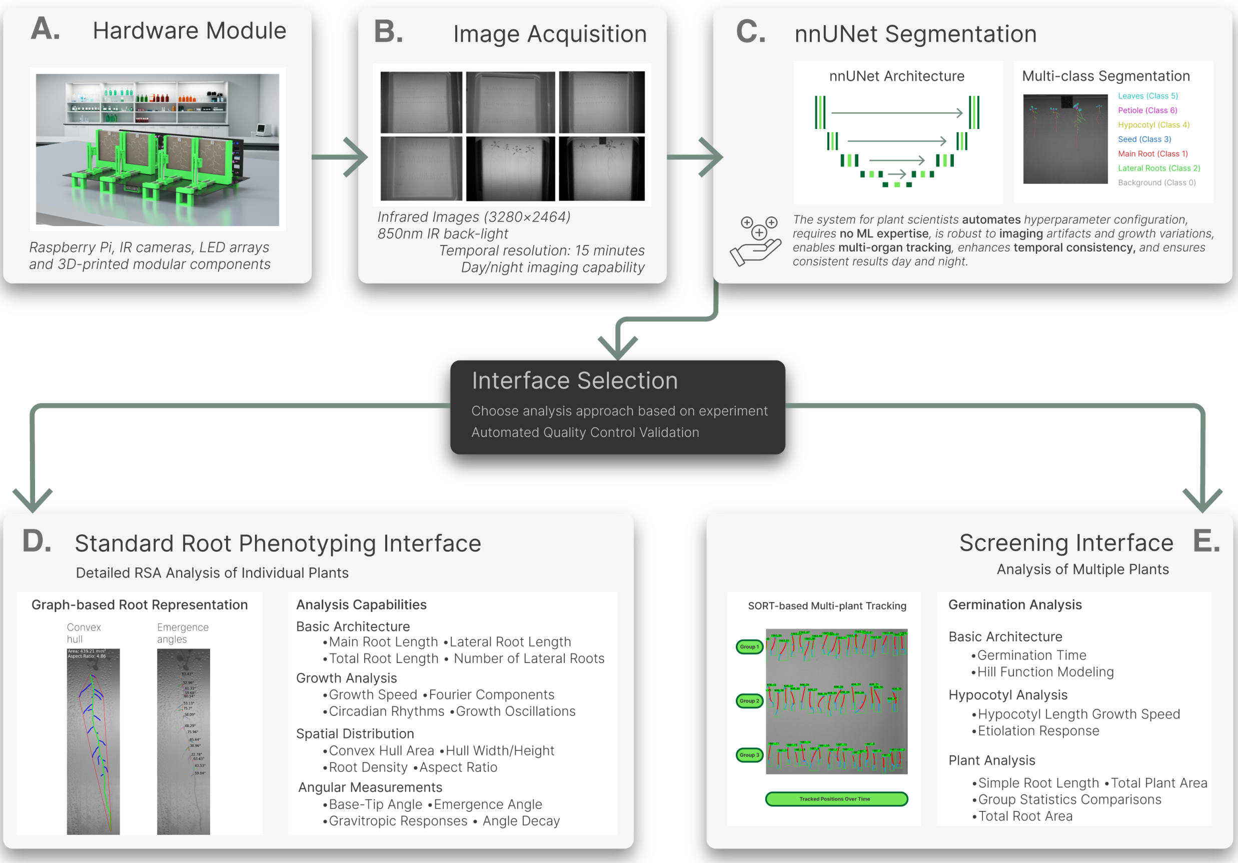

Figure 1. ChronoRoot 2.0: An integrated platform for temporal plant phenotyping. (A) The hardware module combines affordable components for automated imaging in controlled environments. (B) Infrared images are captured continuously, enabling consistent monitoring during both day and night cycles. (C) A multi-class segmentation model based on nnUNet automatically identifies and tracks six plant structures: main root, lateral roots, seed, hypocotyl, leaves, and petiole. The system routes data through an interface selection step, offering two specialized analysis workflows: (D) the Standard Interface for detailed architectural analysis of individual plants, and (E) the Screening Interface for high-throughput experiments involving multiple individuals.

Materials and Methods

System Architecture Overview

ChronoRoot 2.0 is built as a modular software system that integrates hardware control, image processing, and analysis capabilities within a unified framework. The system architecture comprises three main components: a hardware control module for image acquisition using fixed-focus infrared cameras, a deep learning-based segmentation core for multi-class plant structure identification, and two specialized graphical user interfaces designed for different experimental scenarios.

The components operate through a standardized data pipeline: the hardware module captures infrared images at defined intervals, which are then processed by the segmentation core to identify distinct plant structures, before being analyzed through either of the specialized interfaces. The Standard Root Phenotyping Interface enables detailed architectural analysis through graph-based representation, while the Screening Interface facilitates high-throughput analysis of multiple plants through automated plant tracking. This integrated architecture ensures consistent data processing while enabling flexible deployment for various experimental needs.

Hardware Implementation and Image Acquisition

The ChronoRoot device [6] is an affordable and modular imaging system based on 3D-printed and laser cut pieces combined with off-the-shelf electronics. Each module consists of a Raspberry Pi (v3)-embedded computer controlling four fixed-zoom and fixed-focus cameras (RaspiCam v2), and an array of infrared (IR) LED back-light. In between each camera and the corresponding IR array, there is a vertical 12 x 12 cm plate for seedling growth, allowing automatic image acquisition repeatedly along the experiment without any modification or movement of the imaging setup.

The four-plate module is compact (62 x 36 x 20 cm) and can be placed in any standard plant growth chamber. The different parts of the imaging setup (back-light, plate support and camera) can be positioned along a horizontal double-rail to control the field of view of the camera and accurate lighting. In addition, the camera can be moved vertically. ChronoRoot allows image acquisition at a high temporal resolution (a set of pictures every minute). The use of an IR back-light (850 nm) and optional long pass IR filters (> 830 nm) allow acquiring images of the same quality independently from the light conditions required for the experiment, during day and night.

Deep Learning Segmentation Framework

ChronoRoot 2.0's machine learning capabilities rests on a comprehensive dataset of plant developmental sequences that represents a significant expansion from the original ChronoRoot. The dataset comprises 911 manually annotated images of *Arabidopsis thaliana* and 480 images of tomato, capturing multiple plants across various developmental stages. Expert biologists performed detailed annotation using ITK-SNAP [9], chosen for its precise annotation tools and user-friendly interface.

The annotation process captured seven distinct structural

classes:

- Class 0: Background (non-plant regions)
- Class 1: Main root (primary root axis)
- Class 2: Lateral roots (all secondary roots)
- Class 3: Seed (pre- and post-germination structures)
- Class 4: Hypocotyl (stem region between root-shoot junction and cotyledons)
- Class 5: Leaves (including both cotyledons and true leaves)
- Class 6: Petiole (stalk that attaches the leaf to the stem)

In the tomato dataset, leaves and petiole were annotated as a single combined aerial part class, reflecting species-specific morphological differences.

Image acquisition utilized the ChronoRoot hardware system's Raspberry Pi Noir V2 camera, producing infrared images at 3280 x 2464 resolution (approximately ≈ 0.04 mm per pixel). Images are processed at single channel full native resolution, preserving all structural detail throughout both training and inference. To ensure robust model generalization, the dataset incorporates specimens from various experimental conditions and genetic backgrounds.

The segmentation framework implements the nnUNet architecture [7], selected for its self-configuring capabilities and proven performance in biomedical image analysis. The network dynamically adjusts its depth, width, kernel sizes, stride patterns, normalization schemes, and learning rate schedules based on the training dataset's properties, eliminating the need for extensive hyperparameter tuning. This adaptability is particularly valuable in plant phenotyping contexts, where morphological diversity and varying experimental conditions create unique imaging challenges. Our implementation uses the official nnUNet v2 framework, facilitating straightforward adaptation to new plant species through standard retraining procedures. The framework provides two architectural variants: the standard convolutional nnUNet and the recently introduced nnUNet with residual encoder connections [10], which improves feature learning through skip connections in the encoder pathway. Additionally, the framework supports optional test-time augmentation, where predictions from multiple augmented versions of each image are averaged to improve segmentation robustness, particularly for boundary detection.

Specialized Processing Pipelines

ChronoRoot 2.0 implements a multi-stage processing pipeline that begins with temporal consistency enhancement of the segmentation outputs. All segmentations produced by the nnUNet undergo a weighted trailing average approach, with special consideration for the multi-class nature of the predictions. This temporal integration strategy significantly improves tracking robustness by incorporating historical structural information alongside new observations. This temporal averaging is selectively applied only to the main root (class 1) and lateral roots (class 2) channels, as these structures require particular stability for accurate tracking. The accumulation is expressed as $a^t = s^t + \alpha a^{t-1}$, where s^t is the current segmentation at time t, a^{t-1} is the accumulated mask up to the previous time step, and α is a weight factor determined by the temporal resolution of the sequence. This approach effectively addresses common imaging challenges in plant phenotyping, such as water droplets, condensation artifacts, or temporary occlusions, providing stable root structure detection throughout developmental timeframes.

Following this temporal processing, the **Standard Root Phenotyping Interface** implements a Region of Interest (ROI)-based analysis approach similar to the one available in the original ChronoRoot system. After initial segmentation of the full image, this interface requires user interaction to define individual ROIs for each plant to be analyzed. This manual ROI selection is crucial for ensuring accurate, independent processing without interference from neighboring specimens. Within each ROI, the system first performs binary mask refinement through morphological operations and connected component analysis. The subsequent skeletonization and graph construction processes operate solely on the refined binary mask within the current ROI, ensuring that the resulting graph structure represents only the selected plant's root system. This approach enables precise measurement of root system architecture parameters and growth patterns while maintaining the ability to analyze multiple plants from the same image sequence over time through sequential processing.

The **Screening Interface** extends the system's capabilities to high-throughput scenarios through automated multi-plant tracking based on the SORT (Simple Online Realtime Tracking) algorithm [8]. The tracking system begins with robust seed detection through contour analysis of segmentation masks and maintains plant identities across frames through a sophisticated combination of Kalman filtering [11] and the Hungarian [12] algorithm. Kalman filtering enables prediction of plant positions in subsequent frames based on their movement patterns, while the Hungarian algorithm optimizes the association between predicted and detected positions, ensuring reliable tracking even in crowded scenes. The interface supports definition of experimental groups for comparative studies and implements comprehensive quality control mechanisms during data postprocessing, including automatic removal of plants that cover or touch each other or exhibit abnormal movement patterns. This automated approach enables simultaneous but simpler analysis of multiple plants while maintaining measurement accuracy, significantly increasing experimental throughput without compromising data quality.

Analysis Frameworks

The analysis capabilities of ChronoRoot 2.0 comprise three main components: the Standard Root System Architecture Analysis that maintains continuity with the original ChronoRoot system while adding enhanced features, the High-Throughput Screening Analysis that enables efficient processing of multiple plants simultaneously, and a new module implementing Functional Principal Component Analysis (FPCA) that provides sophisticated temporal pattern analysis. In what follows we provide a more detailed description of each module.

Standard Root System Architecture Analysis

The Standard Root Phenotyping Interface provides detailed architectural analysis of individual plant root systems through time. This analysis pipeline builds upon the core capabilities of the original ChronoRoot system while introducing new measurements and enhanced processing methods.

The analysis begins with user definition of ROIs, allowing precise selection of individual plants from multi-plant images. Within these ROIs, the system processes segmentation masks through morphological operations and thinning algorithms to obtain skeletal representations of the root system. These skeletons are then analyzed to identify key nodes, which serve as the basis for constructing a graph representation using a depth-first search algorithm [13].

The graph-based approach, combined with temporal tracking of nodes across frames, enables automatic both node and edge classification and measurement of key architectural features. The system distinguishes between main root and lateral root segments through analysis of the graph structure, with special consideration for complex topologies such as loops where lateral roots reconnect with the main root axis. All measurements provided by ChronoRoot 2.0 are summarized in Table 1, organized into five categories: basic architecture, growth analysis, spatial distribution, angular measurements, and high-throughput analysis. The rightmost column indicates which use case (numbered 1-3) demonstrates the practical application of each metric, with detailed results presented in

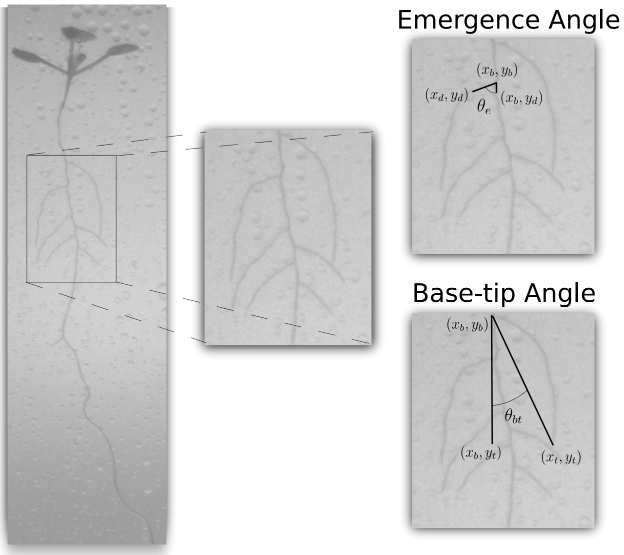

Figure 2. Angular measurements in root system architecture. Illustration of base-tip angle (θ_{bt}) and emergence angle (θ_e) calculations on a *Arabidopsis thaliana* plant showing how these complementary metrics quantify different aspects of lateral root orientation.

the Results section.

Basic architectural parameters capture the fundamental dimensions of the root system through main root length, total lateral root length, and their relationships. Growth dynamics are analyzed through temporal derivatives of these measurements, with special attention to circadian patterns revealed through Fourier analysis of filtered growth speeds. Spatial distribution metrics, computed daily, use convex hull analysis to characterize the overall root system shape and space utilization.

Building upon these established measurements, ChronoRoot 2.0 introduces novel angular parameters that provide detailed insight into lateral root development patterns. These measurements are particularly relevant for quantifying gravitropic responses and directional growth dynamics that are central to plant developmental studies. The measurements leverage both the graph structure, which provides precise identification of lateral root base and tip positions, and the labeled skeleton representation, which enables tracking of root paths for emergence angle calculations.

Two complementary angles are calculated with respect to the vertical axis, where 0 degrees represents perfectly vertical growth. The base-tip angle (θ_{bt}) measures the overall orientation using three reference points: the root base coordinates (x_b, y_b), the root tip coordinates (x_t, y_t), and the vertical projection of the tip (x_b, y_t):

$$\theta_{bt} = \arccos\left(\frac{y_t - y_b}{\sqrt{(x_t - x_b)^2 + (y_t - y_b)^2}}\right) \cdot \frac{180}{\pi} \quad (1)$$

This measurement captures the terminal orientation of the root after all developmental adjustments have occurred. In contrast, the emergence angle (θ_e) quantifies the initial growth trajectory by measuring the angle at a fixed distance d (default 2 mm) from the base:

$$\theta_e = \arccos\left(\frac{y_d - y_b}{\sqrt{(x_d - x_b)^2 + (y_d - y_b)^2}}\right) \cdot \frac{180}{\pi} \quad (2)$$

For these novel angular parameters (illustrated in Figure 2), the system maintains temporal consistency through careful tracking of individual root components between frames. This temporal integration is particularly important for lateral roots, whose identities must be preserved across timepoints to enable reliable measurement of architectural changes throughout development.

To facilitate data exchange and integration with the broader plant phenotyping community, all measurements are exported in the Root System Markup Language (RSML) format [14]. This standardized XML-based format stores the complete hierarchical structure of main and lateral roots along with their spatial coordinates and derived metrics, enabling interoperability with other root phenotyping tools.

To prepare the data for final analysis and visualization, all measurements pass through an automated post-processing script that ensures biological and structural consistency across the time series. To account for skeletonization noise and avoid the inclusion of false-positive "spurs" on the root axes, we implemented a structural pruning threshold where a minimum length of 5 pixels ($\approx$ 0.2 mm) is required for a skeleton branch to be preserved prior to graph construction. Additionally, a measurement post-processing script filters out "false starts" and transient misdetections through a temporal verification window: any detected structure is only validated if it persists beyond a 6-hour threshold. Finally, the pipeline enforces a monotonic growth constraint for length measurements, preventing impossible decreases in plant size over time. While these cleaned results are used for the study figures, the raw segmentations, graphs, and original RSML data remain available for researchers who wish to perform their own specific analyses.

Multiple Plant Screening Analysis

The Screening Interface is designed for efficient analysis of multiple plants simultaneously. This framework comprises three specialized analysis modules, each optimized for specific aspects of plant development while maintaining multiple plants high-throughput processing capabilities, with their corresponding metrics summarized in Table 1 under Multiple Plant Analysis.

The germination analysis module implements validated approaches from previous germination analysis systems [15] to monitor seed morphology changes and detect emergence events. The module employs the Four-Parameter Hill Function to model germination progression:

$$G(t) = G_0 + \frac{G_{max} \cdot t^n}{t_{50}^n + t^n},$$

where G_0 represents the base germination level, G_{max} is the maximum germination percentage, n denotes the steepness parameter, and t_{50} represents the time to 50% germination. The Time of Maximum Germination Rate (TMGR) is calculated as:

$$TMGR = t_{50} \cdot (\frac{n-1}{n+1})^{1/n}.$$

The hypocotyl analysis module incorporates validation steps for reliable measurement in multi-plant scenarios. The system automatically detects physiologically impossible growth rates and artifacts that can occur when different plants touch and their segmentations combine, incorporating biological constraints by forcing non-decreasing length measurements. To ensure full transparency, the platform preserves all raw, unfiltered measurements alongside the processed results. These files are exported in standard formats, allowing researchers to apply custom validation logic or modify the underlying code for specialized needs.

The plant analysis module provides rapid quantification of basic growth parameters through efficient skeletonization techniques. While not as detailed as the graph-based analysis of the Standard Interface, this module extracts fundamental measurements such as main root length, root area, and full plant area, enabling effective high-throughput screening of general growth patterns.

Table 1. Overview of metrics provided by ChronoRoot 2.0. Measurements organized by category: Basic Architecture, Growth Analysis, Spatial Distribution, Angular Measurements, and High-throughput Analysis. The Use Case column indicates which demonstration study (#1: circadian analysis, #2: gravitropic response, #3: etiolation screening) employs each metric.

Category	Metric	Units	Measurement Method	Use Case
Basic Architecture	Main Root (MR) Length	mm	Path length along graph skeleton	1
	Lateral Root (LR) Length	mm	Sum of all LR path lengths	1
	Total Root (TR) Length	mm	MR Length + LR Length	1
	Number of Lateral Roots	count	Unique edges emerging from main root path	1
	Discrete LR Density	LRs/cm	10 * Number of LRs / MR Length	1
	Main Over Total Root	ratio	MR Length / TR Length	1
Growth Analysis*	Growth Speed	mm/h	Temporal derivative of length measurements	1
	Detrended Growth Speeds	mm/h	Raw speeds minus median-filtered signal	1
	Fourier Components	Hz	Fast Fourier Transform of detrended time series	1
Spatial Distribution	Convex Hull Area	mm^2	OpenCV convex hull function	2
	Convex Hull Width	mm	Maximum horizontal extent	2
	Convex Hull Height	mm	Maximum vertical extent	2
	Root Density	mm/mm^2	Root Length / Convex Hull Area	2
	Aspect Ratio	ratio	Height / Width	2
Angular Measurements	Base-Tip Angle	degrees	Angle between vertical and root tip	2
	Emergence Angle	degrees	Angle at 2mm from base	2
Multiple Plant Analysis	Germination Time	hours	Time to radicle emergence	3
	T50	hours	Time to 50% germination	3
	TMGR	hours	Time of maximum germination rate	3
	Final Germination	%	Percentage of germinated seeds	3
	Seed Size	mm^2	Area at experiment start	3
	Hypocotyl Length	mm	Path length of hypocotyl skeleton	3
	Hypocotyl Growth Speed	mm/h	Length difference between timepoints	3
	Total Plant Area	mm^2	Sum of all segmented regions	3
	Simple Root Length	mm	Path length of primary root skeleton	3

*Applicable to any temporal measurement

Functional Data Analysis of Plant Development

A major methodological advancement in ChronoRoot 2.0 is the implementation of FPCA for analyzing temporal patterns in plant development. FPCA [16] represents a significant analytical improvement over conventional time-series approaches by treating growth trajectories as continuous functions rather than discrete measurement points.

While traditional plant growth analysis typically relies on point-wise comparisons or summary statistics, which can miss subtle patterns in developmental dynamics, this approach considers the entire growth curve as a functional unit. This enables detection of complex temporal patterns and variations in growth rates that might be overlooked by conventional methods, particularly valuable in plant development studies where the timing and rate of growth can be as biologically relevant as final measurements. For more details and a more graphical explanation of FPCA, we refer the reader to Supplementary Material S1.

FPCA processes temporal measurements through several steps. First, each growth trajectory is converted into a functional data object using monomial basis expansion, providing a continuous representation of the development pattern. The system then performs dimensionality reduction to extract principal component functions that capture the main modes of variation in the data. These functional components are ranked by their explained variance ratio, with typically 2-3 components accounting for over 90% of the observed variation.

This analysis method can be applied to any temporal measurement extracted by either interface, including root lengths, growth rates, and organ areas. Through quantile-based reconstructions and divergent color palettes, the system provides intuitive visualizations of how components modify developmental trajectories, enabling researchers to detect subtle temporal patterns in growth, identify key time points where developmental trajectories diverge between conditions, and quantify complex growth behaviors through a reduced set of interpretable components.

Software Implementation and User Interface

ChronoRoot 2.0 introduces two dedicated graphical user interfaces developed with Python and PyQt5, replacing the original text-based configuration system. Both interfaces are built upon a shared foundation of scientific computing libraries including NumPy, Pandas, and SciPy for data processing and statistical analysis, OpenCV for image processing, and Matplotlib and Seaborn for visualization.

The Standard Root Phenotyping Interface maintains the core functionality of the original ChronoRoot system while adding modern visualization capabilities. This interface implements a comprehensive analysis pipeline through several interconnected modules (Supplementary Figure S2). The main interface provides tools for experimental configuration, ROI-based plant selection, and real-time visualization of segmentation results. Through an intuitive workflow, users can configure analysis parameters, process individual plants, and generate detailed architectural measurements. The analysis capabilities include convex hull analysis, lateral root angle measurements, growth speed evaluation with Fourier analysis, and detailed statistical testing using Mann-Whitney tests at configurable time intervals. Users can specify particular days for detailed reporting and adjust various measurement parameters such as emergence distance for lateral roots. The interface incorporates quality control through visual feedback systems that allow users to inspect the segmentation results prior to plant selection and manually define the root starting position. Once processed, the software generates growth videos overlaid with the resulting

Table 2. Segmentation performance comparison between original ChronoRoot and ChronoRoot 2.0. The nnUNet implementation outperforms previous models in both accuracy and processing speed in a separated test set (n=55). All nnUNet configurations achieve higher Dice scores than the original models. While test-time augmentation (TTA) shows no significant impact on segmentation overlap (Dice), it substantially improves boundary precision (Hausdorff), reducing error distances by removing spurious segmentations.

Model	Dice	Hausdorff (mm)	Processing Time (s)
Original ChronoRoot			
DSResUNet (Fast)	0.769 ± 0.043	7.25 ± 6.87	~0.5
Ensemble (Accurate)	0.772 ± 0.048	7.21 ± 7.02	~4.5
ChronoRoot 2.0 nnUNet			
Standard	0.809 ± 0.041	6.41 ± 5.39	2.80 ± 0.09
Standard (no TTA)	0.808 ± 0.042	11.08 ± 13.58	0.89 ± 0.07
Residual	0.812 ± 0.038	9.07 ± 9.33	5.34 ± 0.15
Residual (no TTA)	0.815 ± 0.032	13.10 ± 14.18	1.57 ± 0.04

graphs and showcases the measurements, enabling researchers to visually validate the tracking performance for each plant. Problematic individuals can then be discarded or re-analyzed before the system proceeds to the automated generation of comprehensive reports and statistical summaries.

The Screening Interface introduces a streamlined workflow for high-throughput phenotyping experiments (Supplementary Figure S3). The interface guides users through a systematic process from initial calibration to analysis, featuring a dedicated manual calibration tool for precise spatial measurements and an interactive group selection system for defining experimental conditions. Users can define regions of interest corresponding to different treatments or genotypes, and input manual seed counts when needed. The interface implements three specialized analysis modules: germination analysis, hypocotyl development tracking, and basic plant measurements. Real-time visualization tools allow users to monitor segmentation quality, tracking performance, and analysis results as they are generated.

Both interfaces employ multithreading to maintain responsiveness during computationally intensive operations. Quality control mechanisms are integrated throughout the workflows, enabling users to quickly identify and address potential issues. The system generates automated reports featuring graphical summaries and numerical statistics, making experimental results readily available for analysis and publication. The complete codebase and documentation are freely available through our GitHub repository (detailed in the Data Availability section), enabling reproducibility and further development by the community.

Results

The performance and capabilities of ChronoRoot 2.0 were evaluated through four key aspects. First, we assessed the core segmentation capabilities, comparing our nnUNet implementation against the original ChronoRoot system in both accuracy and computational efficiency. Second, we validated the system's multi-class detection capabilities, evaluating its performance in simultaneously identifying and tracking six distinct plant structures across both *Arabidopsis thaliana* and tomato. Third, we demonstrated the system's multi-species capability through comprehensive evaluation on both species, showcasing robust performance across morphologically distinct plants. Finally, we demonstrated the software's practical utility through four comprehensive use cases, showcasing its application in both detailed architectural analysis and high-throughput screening scenarios.

Table 3. Multi-class segmentation performance on the *Arabidopsis thaliana* dataset. All model configurations achieve similar results (n=176). Notably, the fast variants provide a significant reduction in processing time with a minor loss in segmentation performance or structural correctness.

Model	Dice	HD	Cp	Cr	Time (s)
Standard	0.763±0.196	8.519±12.500	0.934±0.074	0.937±0.106	2.821
Standard (Fast)	0.758±0.198	9.199±13.312	0.929±0.082	0.936±0.110	0.972
Residual	0.764±0.193	8.415±11.983	0.930±0.104	0.937±0.109	5.300
Residual (Fast)	0.763±0.189	8.743±12.324	0.926±0.109	0.935±0.113	1.630

Segmentation Performance with nnUNet

We first evaluated the segmentation performance of ChronoRoot 2.0's nnUNet implementation against the original ChronoRoot models using their established dataset (consisting of 339 train images and 55 test images) and metrics (Dice coefficient quantifies the overlap between predicted and ground truth segmentations, while the Hausdorff distance measures the maximum boundary error in millimeters), to validate our architectural improvements. This comparison not only validates the new segmentation approach but also demonstrates backward compatibility with the original system's binary segmentation task, ensuring continuity for existing users while providing enhanced capabilities. The nnUNet implementation showed substantial accuracy gains while maintaining practical processing speeds for high-throughput applications (Table 2).

The original ChronoRoot system offered two operational modes: a rapid DSResUNet implementation (0.5 seconds/image) and a more accurate but slower ensemble method (4.5 seconds/image). While the fast method enabled high-throughput processing, its accuracy (Dice coefficient: 0.769) limited its utility for detailed architectural studies. The ensemble approach achieved marginally better accuracy (Dice: 0.772) but at a significant computational cost.

Regarding ChronoRoot 2.0, we trained two different nnUNet architectural configurations: the standard convolutional architecture and a novel incorporation of a residual encoder architecture [10]. Our implementation allows users to activate or deactivate test-time augmentation (TTA) at inference time, providing a flexible trade-off between processing speed and segmentation quality. All nnUNet configurations substantially outperformed the original ChronoRoot models, achieving Dice coefficients above 0.808 while maintaining practical processing speeds. Disabling TTA reduces inference time by approximately 3-fold (from 2.80 to 0.89 seconds for standard architecture, and from 5.34 to 1.57 seconds for residual), enabling high-throughput processing. Importantly, test-time augmentation showed divergent effects on the two evaluation metrics: TTA had no significant impact on Dice coefficients, comparing architectures with and without TTA revealed nearly identical overlap performance, yet dramatically improved boundary precision as measured by Hausdorff distance, reducing error distances by 40-45%. This improvement stems from TTA's ability to remove spurious segmentations through prediction averaging, which primarily affects boundary outliers rather than overall segmentation overlap.

All training and inference time evaluations were conducted on a standard workstation equipped with an Intel(R) Core(TM) i7-8700 CPU, 64 GB RAM, and an NVIDIA Titan X GPU.

Multi-Class Segmentation Performance

Building upon these improvements in binary segmentation, we evaluated the nnUNet's performance in discriminating among six distinct plant structures. This multi-class capability represents a significant advancement over the original system, enabling tracking of multiple plant organs throughout development. The dataset was partitioned, within each of the three major experimental categories (etiolation, germination, and plant root analysis), into training (70%), validation (10%), and test (20%) sets following a video-based splitting strategy to prevent data leakage.

Table 4. Cross-species generalization and training strategy evaluation for the tomato dataset. Residual architectures show better results in Hausdorff Distance (HD) and Correctness (Cr), in the separated test set (n=181). The multi-species training strategy (Both) consistently outperforms training only on tomato data, showing that data diversity improves results across different morphologies.

Training	Configuration	Dice	HD	Cp	Cr	Time (s)
Tomato	Standard	0.815±0.218	19.793±20.590	0.920±0.133	0.779±0.230	2.198
Tomato	Standard (Fast)	0.801±0.220	24.357±22.973	0.910±0.157	0.733±0.253	0.813
Tomato	Residual	0.843±0.200	15.430±18.900	0.908±0.153	0.868±0.189	5.290
Tomato	Residual (Fast)	0.829±0.206	17.295±19.492	0.904±0.159	0.853±0.197	1.586
Both	Standard	0.828±0.212	19.803±20.335	0.914±0.141	0.816±0.223	2.199
Both	Standard (Fast)	0.822±0.214	19.722±19.897	0.908±0.156	0.791±0.232	0.768
Both	Residual	0.863±0.201	11.089±16.640	0.916±0.138	0.896±0.177	5.310
Both	Residual (Fast)	0.858±0.195	11.553±16.174	0.905±0.165	0.899±0.172	1.557

Beyond standard segmentation overlap metrics, successful root system analysis depends critically on preserving key morphological traits. We therefore evaluated the skeletonized root segmentations using completeness and correctness metrics, which directly assess structural fidelity [17]. Completeness measures the extent to which the extracted skeleton retains the original root structure, with higher values indicating fewer missing segments. Correctness evaluates the presence of extraneous or spurious branches in the extracted skeleton, with high values indicating that the segmentation accurately follows the true root architecture without introducing artifacts.

Table 3 presents the overall performance averaged across all plant structures, and the completeness and correctness calculated for the complete root, for each model configuration. All variants achieved similar Dice coefficients, with the standard and residual architectures showing no significant differences between them, but both significantly outperforming their respective fast (non-TTA) variants according to Wilcoxon Pair Ranked Test. Processing times ranged from 1 to 5 seconds per image, with the fast variants providing approximately 3-fold speedup. Detailed per plant organ values are shown in Supplementary Table S1.

Across species generalization: Tomato.

To evaluate the generalizability of our approach to other plant species, we trained and tested nnUNet models on a tomato dataset. The data was partitioned by experimental setup, with one complete acquisition (24 plates) reserved for testing, resulting in 299 training images and 181 test images. Note that in the tomato dataset, leaves and petioles were annotated as a single combined aerial part class, reflecting species-specific morphological differences from *Arabidopsis thaliana*.

We evaluated two training strategies: (1) models trained exclusively on tomato data, and (2) models trained on combined tomato and *Arabidopsis thaliana* datasets. For the combined training approach, *Arabidopsis* annotations were preprocessed to match the tomato class structure by merging leaf and petiole classes into a single aerial part category. All four model configurations (Standard, Standard Fast, Residual, and Residual Fast) were evaluated under both training regimes.

The results (Table 4) reveal several important findings. First, residual architectures consistently outperformed standard architectures across all metrics, with particularly notable improvements in Hausdorff distance and correctness measures. Second, incorporating *Arabidopsis* training data significantly enhanced performance across all model configurations, with the combined training strategy yielding the best results. These findings demonstrate both the transferability of knowledge across plant species and the value of diverse training data for robust segmentation performance. Detailed per plant organ values are shown in Supplementary Table S2.

Demonstration of Software Capabilities Through Use Cases

To validate ChronoRoot 2.0's practical utility across diverse experimental scenarios, we implemented three use cases. The first one examines root system architecture under long day and continuous light condition following the original publication [6], while the second analyzes published data from the transcription factor gene *NF-YA10* over-expressing plants [18]. The final use case demonstrates the high-throughput screening capabilities of the system, on an etiolation experiment. Importantly, all figure subpanels presented in these use cases are direct outputs from ChronoRoot 2.0 and serve as representative examples of the automated reports generated when users analyze their own data or the provided demo datasets. The only modification to these outputs is the addition of asterisks to indicate statistical significance: * for p<0.05 and ** for p<0.001. While the figures prioritize visual clarity for phenotype comparison, the exact statistical analysis values—including p-values, means, and standard deviations—are provided by the software as accompanying text files within the output folders, requiring no additional analysis beyond what the software automatically generates.

Use Case 1 - Temporal Dynamics of Root System Architecture: Replication and Extension of ChronoRoot Findings with Fourier and FPCA

Plant materials: *Arabidopsis thaliana* ecotype Col-0 seeds were surface sterilized and stratified at 4°C for 2d before being grown under long day conditions (16h light, 140$\mu Em^{-2}s^{-1}$/ 8h dark), or continuous light (24h light, 140$\mu Em^{-2}s^{-1}$) at 22°C, on half-strength Murashige and Skoog media (1/2 MS) (Duchefa, Netherlands) with 0.8% plant agar (Duchefa, Netherlands). Four seeds were used per plate.

Root system architecture exhibits complex temporal dynamics that can reveal fundamental aspects of plant adaptation to environmental conditions. Building upon the findings reported in [6], we investigated how different light regimes influence root development patterns, leveraging our enhanced analytical capabilities to uncover subtle temporal variations in growth dynamics.

To validate and extend the findings from ChronoRoot, we replicated its analysis pipeline and incorporated FPCA to further dissect the temporal dynamics of RSA. First, we computed conventional RSA metrics, including main root length, lateral root length, total root length, lateral root density, and the proportion of the main root relative to total root length (Figure 3-A). We then explored root growth dynamics by applying FPCA to the temporal evolution of root length.

The first functional principal component (PC1) captured the primary growth trajectory of roots (Figure 3-B), revealing differences between photoperiod conditions. The second (PC2) showed distinct divergence between long-day and continuous-light conditions, indicating temporal shifts in growth patterns. Following the ChronoRoot methodology, we also analyzed root elongation rates through Fourier Transform (Figure 3-C) to detect underlying oscillatory patterns, identifying both circadian (24-hour) and ultradian (12-hour) rhythms under long-day conditions, which were disrupted under continuous light. Similar analysis of lateral root length (Figure 3-D) showed comparable patterns.

Use Case 2 - Complete RSA Characterization of different Arabidopsis thaliana genotypes: Area covered, lateral root angles and tip angle decay over time

Plant materials: All plants used in this study are in Columbia-0 background. pNF-YA10:GFP-NF-YA10miRres (NF-YA10miRres) stable lines were obtained by transforming *Arabidopsis* plants with a construct bearing 2000 bp region upstream of the start codon of NF-YA10 amplified from genomic DNA (promoter region) and the coding sequence (CDS) of *NF-YA10* without miRNA cleavage site amplified from cDNA, thus resisting miR169-mediated post-transcriptional silencing of *NF-YA10* mRNA. More details were pub-

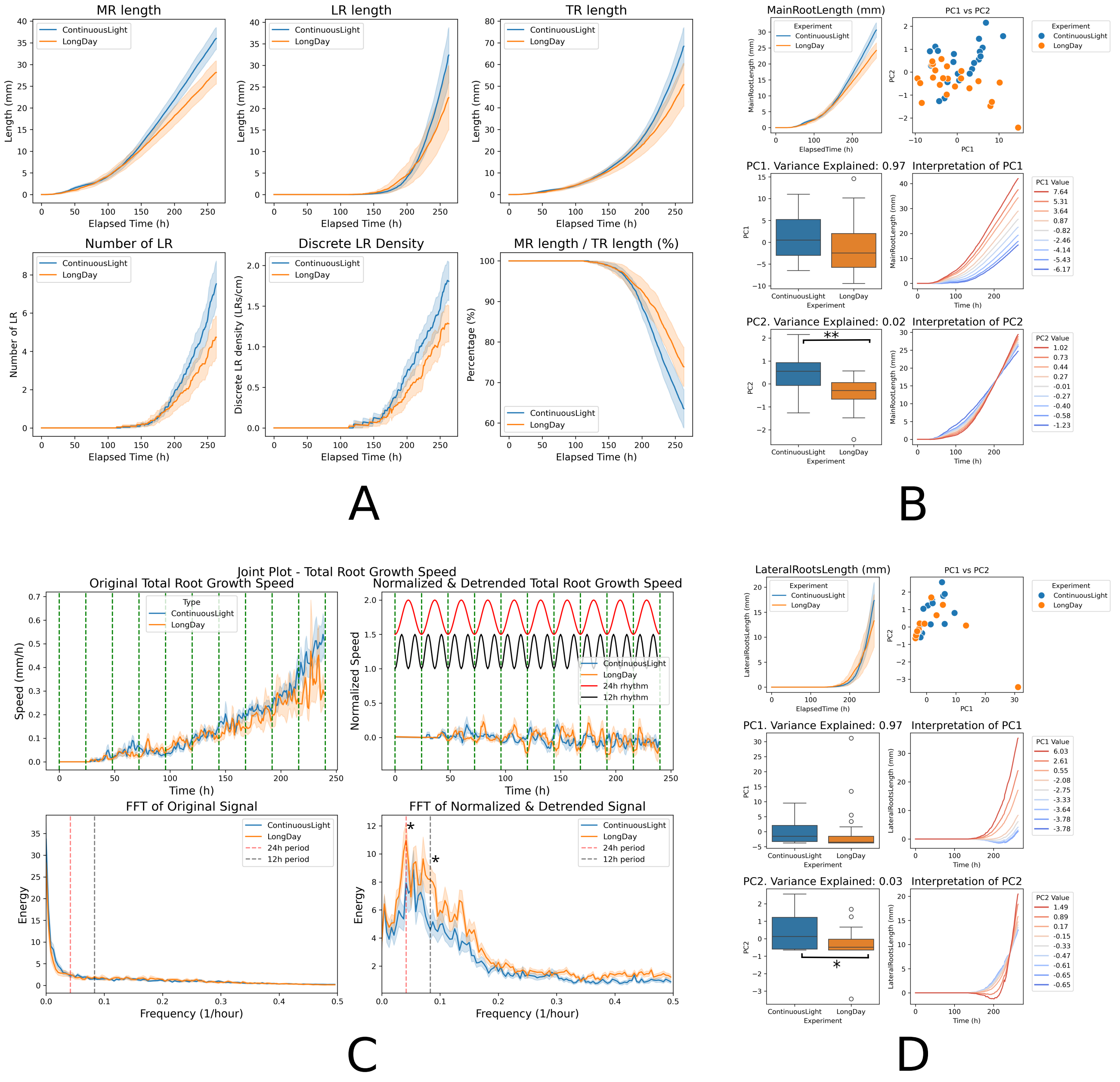

Figure 3. Use Case 1 - *Arabidopsis thaliana* Root system architecture dynamics under different light conditions. Comparison of long-day (16h/8h, blue, n=23) versus continuous light (24h, orange, n=21) shows divergent growth patterns. A - All basic Architectural RSA parameters, B - FPCA analysis of Main Root Length, significative differences found in PC2 (p-value<0.001), C - Fourier transform of Total Root Growth Rate (significative differences found at both 24h and 12h periods, p-value<0.05), D - FPCA analysis of Lateral Root Length, significative differences found in PC2 (p-value<0.05). Error bands: standard error.

lished at [18].

ChronoRoot 2.0's enhanced analytical capabilities revealed distinct architectural patterns between NF-YA10miRres and wild-type Col0 plants. Using the convex hull analysis (Figure 4-A and B), we quantified the overall root system distribution. Qualitative visualization (Figure 4-A) and quantitative metrics (Figure 4-B) showed that NF-YA10miRres plants developed significantly larger convex hull areas, indicating broader root system coverage. Moreover, these plants exhibited higher aspect ratios (height/width), suggesting that lateral roots grew at wider angles from the main root axis rather than clustering around it.

The novel angle measurement capabilities provided detailed insights into these architectural differences. During normal root development, lateral roots typically exhibit gravitropic responses, gradually bending downward after emergence - a phenomenon we term 'angle decay'. Temporal analysis of these lateral root angles (Figure 4-C) showed that NF-YA10miRres plants consistently maintained larger angles compared to wild-type, indicating an altered gravitropic response. The base-tip angle difference progressively increased, reaching a 20° differential after three days of growth. The emergence angles showed similar trends, becoming significantly different from wild-type by day 9. This temporal progression of angular differences suggests that the transcription factor NF-YA10 plays a role in regulating both the initial trajectory and subsequent gravitropic responses of lateral roots.

Use Case 3 - High-throughput Analysis of Etiolation in Arabidopsis thaliana seedlings

Plant materials: Lines G1, G2, and G3 were in the Columbia-0 (Col0) background. Seeds were surface sterilized and sown on MS medium supplemented with 1% agar in 120mm-side square petri dishes. To maximize the experimental throughput, up to 100 seeds were placed in each plate in a grid pattern, with 3 rows of 33 seeds.

To demonstrate ChronoRoot 2.0's capabilities for high-

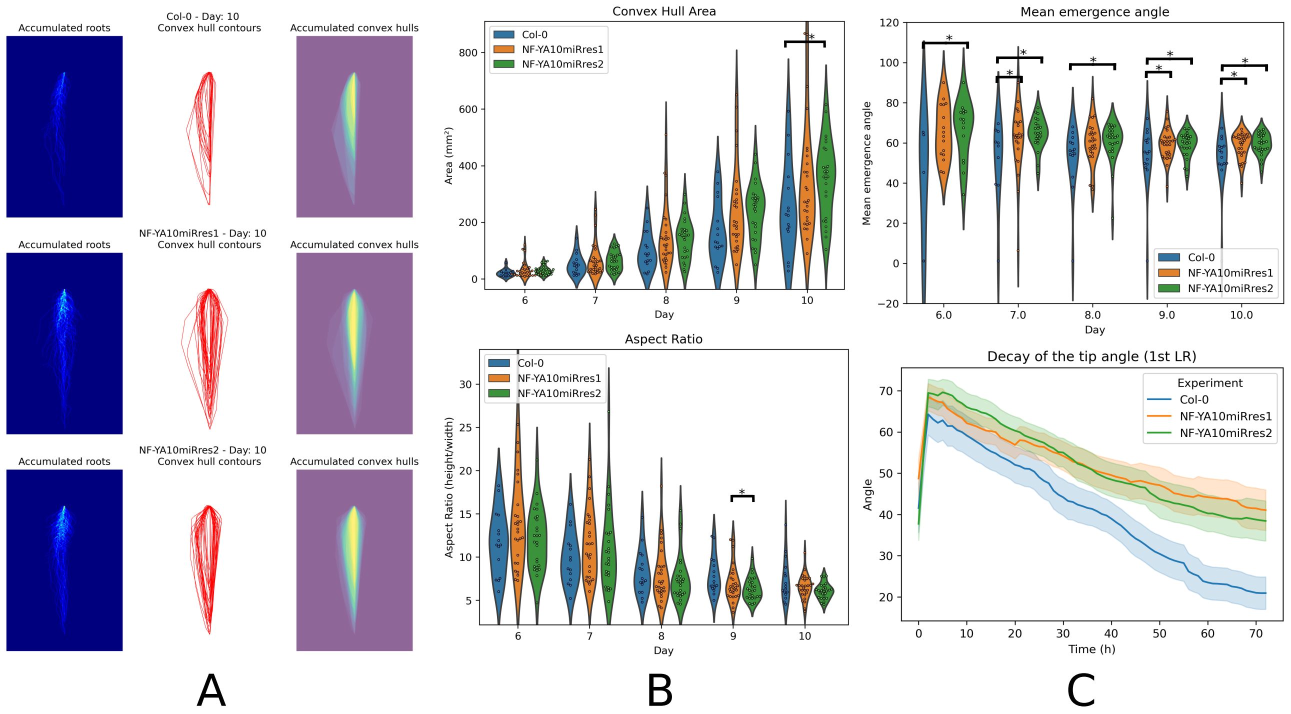

Figure 4. Use Case 2 - Altered root architecture in *Arabidopsis thaliana* NF-YA10miRes plants. A - Qualitative analysis of convex hull showing root system coverage differences between genotypes. B - Quantitative analysis of convex hull metrics (area and aspect ratio) between NF-YA10miRes plants (1 in orange, n=26), 2 in green, n=28) and Col0 controls (blue, n=16). C - Quantitative analysis of average emergence angle and base-tip angle for the first lateral root, demonstrating consistently wider angles in NF-YA10miRes plants compared to Col0. Error bars: standard error.

throughput phenotyping under specific growth conditions, we conducted an etiolation study across three genotypes. After brief light exposure for germination synchronization, plants were grown in complete darkness for 5 days, with automated infrared imaging every 15 minutes to track development without light interference.

The system's enhanced segmentation algorithm successfully distinguished between hypocotyl and root tissues, enabling precise quantification of both structures' growth dynamics. It was also able to identify smaller structures like the cotyledons (embryonic leaves) and the seed coverage (Figure 5-A). All genotypes exhibited the characteristic etiolation response, with dramatic hypocotyl elongation as seedlings searched for light. Hypocotyl length measurements revealed significant differences in elongation between the three genotypes, with Genotype G3 exhibiting the largest hypocotyl, followed by G1 and then G2 (Figure 5-B). The same differences can be appreciated in the growth rate curves, showing that G3 had more sustained growth, followed by G1 and then G2 (Figure 5-B). Analysis of root system development showed the same temporal pattern as the hypocotyl: growth during the first 3 days followed by complete growth cessation after day 4, marking the exhaustion of seed reserves under dark conditions. This pattern was clearly visible in both main root length progression and area coverage metrics (Figure 5-B).

All genotypes showed the germination of 50% of the seedlings between 13.04 and 13.78 h post light stimulation and did not differ significantly (Figure 5-C). This implied that hypocotyl length differences are due to higher growth rate or sustained growth rather than differences on germination time.

Comparison with manual hypocotyl measurements performed on the same dataset showed no statistically significant differences, demonstrating the robustness of the segmentation and measuring process (Figure 5-D). We compared measures at 36, 48 and 60 hours for the three genotypes.

To further characterize developmental patterns across genotypes, we applied FPCA to the growth trajectories (Supplementary Figure S4). This analysis revealed that over 97% of the variance in hypocotyl length, root length, and total plant area could be explained by just two principal components. The first component (PC1) primarily captured differences in final plant size/length, while the second component (PC2) represented temporal shifts in the growth pattern, similarly to the PC2 in Use Case 1. FPCA scores confirmed the genotype differences observed in the direct measurements, with G3 showing significantly higher PC1 scores for hypocotyl elongation and area development, followed by G1 and then G2, as expected.

Use Case 4 - Multi-species Capability: Tomato Analysis

Plant materials: Tomato seeds of cultivar M82 were surface sterilized and sown on MS medium under standard conditions. To accommodate the larger size of tomato seedlings compared to *Arabidopsis*, plant density was reduced to two seeds per plate.

To further demonstrate the species-agnostic design of ChronoRoot 2.0, we applied the complete analysis pipeline to tomato seedlings, which present larger organs, thicker roots, and increased curvature compared to *Arabidopsis*. The nnUNet-based segmentation accurately identified and tracked main roots, lateral roots, and hypocotyls over time in both wild type and mutant plants (Figure 6-A), enabling the extraction of standard architectural and temporal traits without parameter tuning.

Quantitative analysis revealed marked differences between the two genotypes. Spatial descriptors such as accumulated root traces and convex hulls showed that mutant seedlings explored a substantially smaller area than wild type plants (Figure 6-B). Temporal measurements of main root, lateral root, hypocotyl, and total length highlighted an early and persistent reduction in growth in the mutant condition (Figure 6-D). Principal component analysis of the extracted traits captured most of the variance with the first two components and clearly separated wild type and mutant populations, reflecting differences in overall growth magnitude and temporal progression (Figure 6-C).

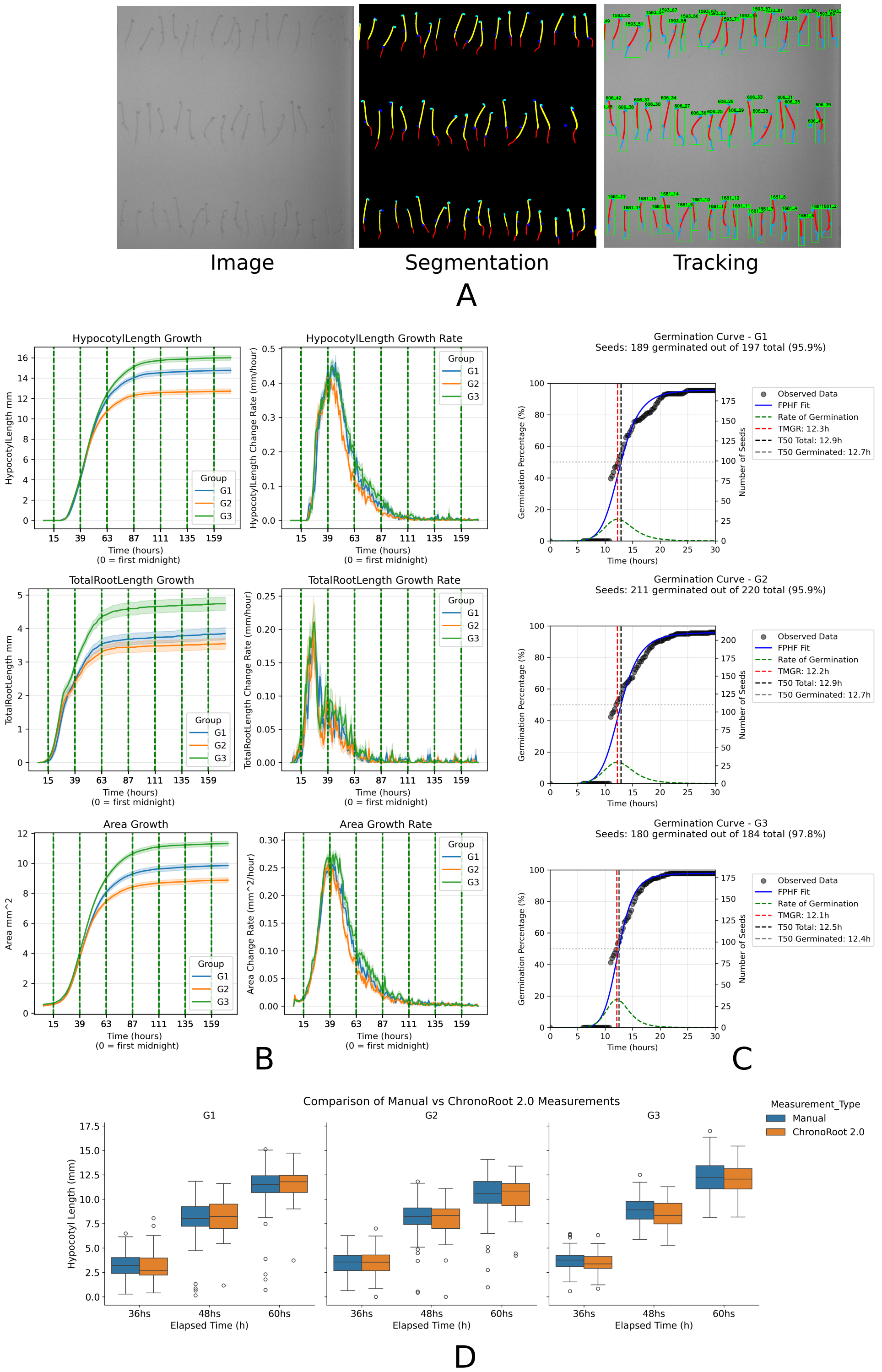

Figure 5. Use Case 3 - High-throughput analysis of *Arabidopsis thaliana* seedling etiolation. A - Representative infrared images showing temporal progression of etiolated seedling development. B - Hypocotyl, root and total area measurements with their corresponding growth rates (G1, blue, n=189. G2, orange, n=211; G3, green, n=180). C - Germination curves showed T50 at approximately 13 hs after light stimulation with no significant differences between the analyzed genotypes. D - Comparison of Manual (blue) and automatic (orange) hypocotyl length determinations at 36, 48 and 60 hours after light stimulation (no significant difference observed). Error bars: standard error.

Discussion

ChronoRoot 2.0 provides plant biologists with an integrated solution for analyzing root system development across multiple experimental scales, integrated with additional parameters of the seedling aerial organs. While the artificial nature of 2D growth systems on petri dishes represents an inherent limitation in root architecture studies, our results demonstrate how enhanced measurement capabilities can reveal meaningful biological patterns even within these constraints.

The multi-class segmentation approach addresses a significant challenge in developmental studies by enabling simultaneous analysis of multiple plant structures. Although root growth on agar plates differs from soil conditions, the ability to precisely track both below and above-ground organs provides valuable insights into developmental coordination. The etiolation response study demonstrates

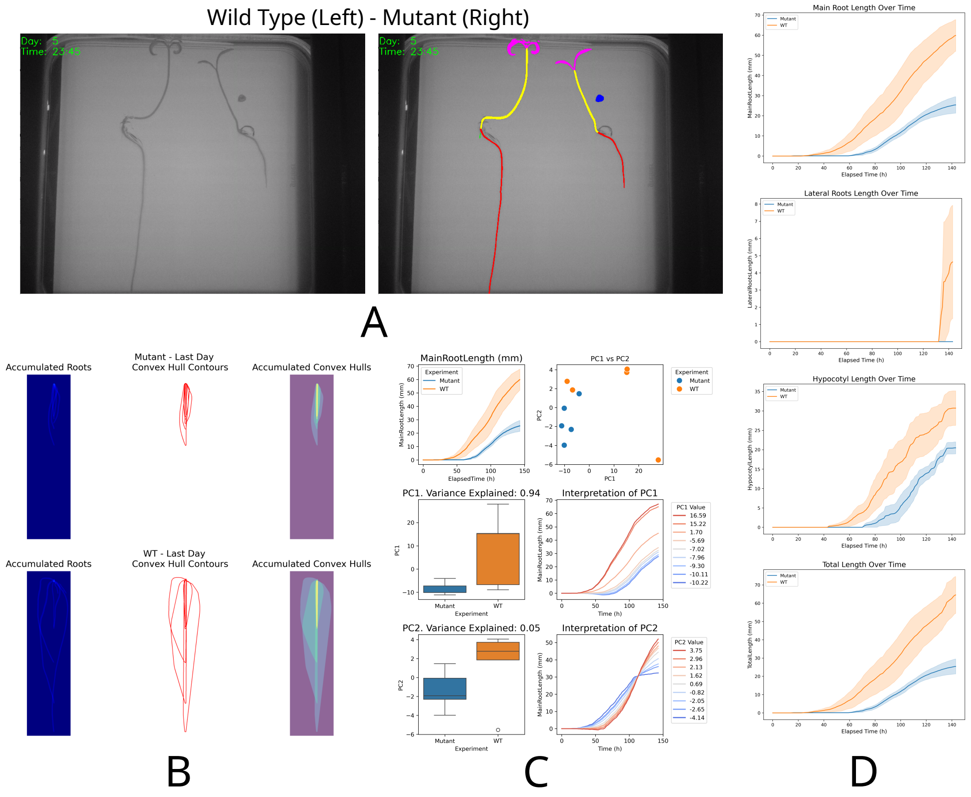

Figure 6. Use Case 4 - ChronoRoot 2.0 multi-species analysis on tomato seedlings. A - Representative time-lapse images of wild type (WT, left, n=5) and mutant (right, n=5) tomato seedlings, with nnUNet-based segmentation and organ tracking overlays. B - Spatial characterization of root system architecture showing accumulated root traces, last-day convex hull contours, and occupied growth area for mutant (top) and WT (bottom). C - Multivariate analysis of extracted traits: main root length dynamics, PCA variance explanation, boxplots for PC1 and PC2, and temporal interpretation of principal components. D - Organ-specific temporal growth curves for main root, lateral roots, hypocotyl, and total length, comparing WT and mutant seedlings.

how this capability can reveal resource allocation patterns during early development, with the simultaneous tracking of hypocotyl elongation and root growth providing a more complete understanding of seedling responses to dark conditions. To demonstrate the system's adaptability beyond *Arabidopsis thaliana*, we incorporated a tomato (*Solanum lycopersicum*) dataset. This addition validates that the self-configuring nnUNet core can be effectively retrained to handle the more robust and diverse morphologies of crop species. While applying the system to plants with fundamentally different architectures would require new annotated training data, this framework ensures that the adaptation process remains accessible to researchers without deep machine learning expertise.

While the software architecture is fundamentally modality-agnostic and offers a modular pathway for adaptation to other 2D imaging platforms (such as SPIRO [15]), the platform's robustness is anchored in its integration with our custom hardware. By coupling the software with an open-source, affordable, and easily assembled hardware unit, we ensure high data quality and stable temporal resolution (15-minute intervals) without presenting a significant financial barrier to adoption.

To maximize the utility of these segmentation capabilities within the spatial limits of 12x12 cm plates, we implemented a dual-interface system designed to handle specific experimental constraints. The *Standard Interface* is designed for high-precision architectural tracking of normally 4-6 plants per plate. These experiments are typically limited to 10-14 days for *Arabidopsis* or 5-7 days for tomato, concluding when the main root reaches the plate bottom or grows along the surface invisible to imaging. Conversely, the *Screening Interface* accommodates up to 100 plants per plate but limits analysis to the early developmental window (typically 3-5 days). This mode allows for massive data collection before plant crowding and physical contact prevent reliable segmentation.

The automated angle measurements introduce new possibilities for quantifying gravitropic responses in standardized conditions. While plate-based growth systems impose spatial constraints on root architecture, the precise measurement of emergence angles and their temporal evolution, as demonstrated in the NF-YA10miRres vs. Col0 analysis, enables systematic study of gravitropic regulation. These measurements provide a standardized framework for comparing gravitropic responses across genotypes and conditions, even within the limitations of 2D growth systems.

The temporal analysis capabilities represent a particular strength for understanding dynamic developmental processes. The identification of distinct growth rhythms under different light conditions demonstrates how high-resolution temporal data can reveal patterns that might be missed by endpoint measurements. While circadian patterns in artificial growth conditions may differ from natural environments, the ability to detect and quantify these rhythms provides valuable insights into the temporal organization of plant development. Moreover, the incorporation of FPCA-based analysis facilitates the interpretation of complex temporal signals by reducing their dimensionality, providing a novel and easily-explainable way to quantify dynamic growth patterns.

The analysis frameworks implemented in ChronoRoot 2.0 open new possibilities for understanding plant development, even within the constraints of traditional growth systems. The ability to automatically quantify subtle architectural differences and temporal patterns enables systematic comparison of developmental responses across genotypes and conditions. These capabilities are particularly valuable for studies investigating the genetic and environmental regulation of plant development, where precise quantification of phenotypic differences is essential. Furthermore, high-throughput comprehensive phenotyping emerges as a powerful tool for genome-wide association studies and the identification of key genes participating in plant development.

ChronoRoot 2.0's release as an open-source platform represents

our commitment to accessible, community-driven plant phenotyping tools. While the current implementation provides robust capabilities for analyzing plate-based growth experiments, the modular architecture and comprehensive documentation enable researchers to adapt and extend the system for their specific needs. By releasing both the software and hardware specifications openly, we aim to foster a collaborative community where researchers can share improvements, analytical modules, and experimental protocols. We hope that this approach to open science will not only ensure transparency and reproducibility but also allow the system to evolve alongside the changing needs of the plant biology community.

Code and Data Availability

The data supporting the findings of this study consists of three main components:

- The complete source code of ChronoRoot 2.0, including the implementation of all analysis methods described in this paper, is freely available under the GNU General Public License v3.0 at https://github.com/ChronoRoot/ChronoRoot2. This repository contains the full software implementation and comprehensive documentation to set up the system and utilizing the software.
  - Project name: ChronoRoot 2.0
  - Project home page: https://chronoroot.github.io
  - Main Source Code repository: https://github.com/ChronoRoot/ChronoRoot2
  - Operating system(s): Platform independent
  - Programming language: Python
  - Other requirements: Conda, Apptainer, or Docker
  - License: GNU GPL 3.0
- The annotated image dataset used for training and validation contains 911 infrared images of *Arabidopsis thaliana* seedlings and 480 images of tomato with expert annotations for multiclass segmentation. This dataset is publicly available without restrictions at https://huggingface.co/datasets/ngaggion/ChronoRoot2. The dataset includes both raw images and their corresponding multi-class segmentation masks in .nii.gz format, as directly generated in the manual annotations made by our biologists. Scripts to convert to nnUNet's standardized structure for 2D images are also provided within the GitHub repo, generating the correct splits to avoid mixing videos in training, validation and test partitions.
- To facilitate reproducibility and allow users to familiarize themselves with the different analysis modules, we provide four complete demo datasets covering the scenarios presented in this paper. These datasets are available within the apptaniner and Docker images, via the ChronoRoot website, and as Supplementary Material to this manuscript:
  i. **Detailed Root Analysis:** A video for RSA characterization of individual *Arabidopsis* plants.
  ii. **Germination Screening:** A video containing hundreds of seeds to test the germination analysis module.
  iii. **Etiolation Screening:** A video of seedlings grown in darkness for testing hypocotyl growth rates.
  iv. **Tomato Comparison:** A pair of videos of tomato illustrating cross-species capability.
- To facilitate deployment and ensure reproducibility across different computing environments, we provide a pre-configured Docker image at https://hub.docker.com/r/ngaggion/chronoroot. This image includes all necessary dependencies and can be used without any installation requirements beyond Docker itself.

Declarations

List of abbreviations

FPCA: Functional principal component analysis;
GPU: graphical processing unit;
IR: infrared;
LR: lateral root;
MR: main root;
PC: principal component;
ROI: region of interest;
RSA: root system architecture;
RSML: Root System Markup Language;
SORT: Simple online realtime tracking algorithm;
TMGR: Time of maximum germination rate;
TR: total root;

Ethical Approval

Not applicable.

Consent for publication

Not applicable.

Competing Interests

The authors declare that they have no competing interests.

Funding

AB, NG, TB, MC and FA benefit from the ECOS-SUD Exchange Program (no. A20N05) and the IRP LOCOSYM (CNRS). The FA lab is funded by Agencia I+D+i, ICGEB and AXA Research Fund. AB, MC and TB benefited from the support of the French Agence Nationale de la Recherche (Saclay Plant Sciences-SPS, ANR-17-EUR-0007).

Author's Contributions

- N. Gaggion led the project implementation, developed all software components, and wrote the manuscript
- R. Bonazzola conceptualized and implemented the Functional Principal Component Analysis methodology
- F. Accavallo contributed to the development and enhancement of the ChronoRoot hardware system
- F. Ariel, A. Barrios, F. Catulo, T. Blein and N. Boccardo conducted the biological experiments analyzed in the study
- F. S. Rodriguez, F. E. Aballay, F. B. Catulo, M. F. Mammarella, M. F. Legascue, and S. N. Villarreal performed manual image annotation for training the Arabidopsis thaliana nnUNet model.
- N. Boccardo and L. Santoro performed manual image annotation for training the tomato nnUNet model.
- L. I. Pereyra-Bistrain and M. Benhamed identified the gene involved in root development, generated the tomato mutant and provided the plant material for Use Case 4.
- F. B. Catulo and M. M. Ricardi performed the validation of the hypocotyl length analyzes
- F. Ariel, E. Petrillo, M. M. Ricardi, T. Blein and M. Crespi provided oversight of all biological experiments and analyses, and evaluated the quality of the generated reports
- E. Ferrante provided oversight of the computational experiments and supervised the software design process
- T. Blein, N. Gaggion, M. Crespi, F. Ariel, and E. Ferrante contributed to the original ChronoRoot concept and its evolution to

version 2.0, and provided manuscript revision

Acknowledgements

Not applicable.

Supplementary Materials

The following supplementary materials are provided to ensure reproducibility and facilitate adoption of the ChronoRoot 2.0 system.

Text S1 Functional PCA
Provides an intuitive explanation of functional principal component analysis (FPCA) for readers without a quantitative background.

Figure S1 Functional principal component decomposition of simulated curves. Illustrates the fundamental concepts of FPCA through simplified example data.

Figure S2 The Standard Root Phenotyping Interface
Demonstrates the complete workflow of the detailed architectural analysis pipeline through six tabs: Plant Analysis (main screen), Preview Image, Analysis Overview, Plant Overlay, Generate Report, and Report.

Figure S3 The Screening Interface
Illustrates the high-throughput analysis workflow through four tabs: Analysis (main screen), Preview Image, Results, and Reports for efficient multi-plant phenotyping.

Table S1 Detailed report of segmentation performance across plant organs for *Arabidopsis thaliana*.

Table S2 Detailed report of segmentation performance for tomato

Figure S4 Functional PCA applied to etiolation experiment.

Presents FPCA of hypocotyl length, root length, and area growth curves from Use Case 3, showing mean trajectories by genotype, principal component distributions, and visual interpretations of how PC1 and PC2 modulate developmental patterns across genotypes G1, G2, and G3.

References

1. Palmer CM, Bush SM, Maloof JN. Phenotypic and Developmental Plasticity in Plants. In: John Wiley & Sons, Ltd, editor. eLS, vol. 59 Chichester, UK: John Wiley & Sons, Ltd; 2001.p. 1127.
2. Tracy SR, Nagel KA, Postma JA, Fassbender H, Wasson A, Watt M. Crop Improvement from Phenotyping Roots: Highlights Reveal Expanding Opportunities. Trends Plant Sci 2020 Jan;25(1):105–118.
3. Ingram PA, Malamy JE. In: Root System Architecture, vol. 55 of Advances in Botanical Research Elsevier; 2010. p. 75–117.
4. Narisetti N, Henke M, Seiler C, Shi R, Junker A, Altmann T, et al. Semi-automated Root Image Analysis (saRIA). Sci Rep 2019 Dec;9(1):19674.
5. Yasrab R, Atkinson JA, Wells DM, French AP, Pridmore TP, Pound MP. RootNav 2.0: Deep learning for automatic navigation of complex plant root architectures. Gigascience 2019 Nov;8(11).
6. Gaggion N, Ariel F, Daric V, Lambert E, Legendre S, Roulé T, et al. ChronoRoot: High-throughput phenotyping by deep segmentation networks reveals novel temporal parameters of plant root system architecture. GigaScience 2021;10(7):giab052.
7. Isensee F, Jaeger PF, Kohl SA, Petersen J, Maier-Hein KH. nnU-Net: a self-configuring method for deep learning-based biomedical image segmentation. Nature methods 2021;18(2):203–211.
8. Bewley A, Ge Z, Ott L, Ramos F, Upcroft B. Simple online and realtime tracking. In: 2016 IEEE international conference on image processing (ICIP) IEEE; 2016. p. 3464–3468.
9. Yushkevich PA, Gao Y, Gerig G. ITK-SNAP: An interactive tool for semi-automatic segmentation of multi-modality biomedical images. In: 2016 38th annual international conference of the IEEE engineering in medicine and biology society (EMBC) IEEE; 2016. p. 3342–3345.
10. Isensee F, Wald T, Ulrich C, Baumgartner M, Roy S, Maier-Hein K, et al. nnu-net revisited: A call for rigorous validation in 3d medical image segmentation. In: International Conference on Medical Image Computing and Computer-Assisted Intervention Springer; 2024. p. 488–498.
11. Welch G, Bishop G, et al. An introduction to the Kalman filter. University of North Carolina at Chapel Hill 1995;.
12. Kuhn HW. The Hungarian method for the assignment problem. Naval research logistics quarterly 1955;2(1-2):83–97.
13. Cormen TH, Leiserson CE, Rivest RL, Stein C. Introduction To Algorithms. MIT Press; 2001.
14. RootSystemML home page;. http://rootsystemml.github.io/, Accessed: 2025-02-04.
15. Ohlsson JA, Leong JX, Elander PH, Ballhaus F, Holla S, Dauphinee AN, et al. SPIRO–the automated Petri plate imaging platform designed by biologists, for biologists. The Plant Journal 2024;118(2):584–600.
16. Shang HL. A survey of functional principal component analysis. AStA Advances in Statistical Analysis 2014;98:121–142.
17. Youssef R, Ricordeau A, Sevestre-Ghalila S, Benazza-Benyahya A. Evaluation protocol of skeletonization applied to grayscale curvilinear structures. In: 2015 International Conference on Digital Image Computing: Techniques and Applications (DICTA) IEEE; 2015. p. 1–6.
18. Barrios A, Gaggion N, Mansilla N, Blein T, Sorin C, Lucero L, et al. The transcription factor NF-YA10 determines the area explored by Arabidopsis thaliana roots and directly regulates LAZY genes. The Plant Journal 2025;121(5):e70016. https://onlinelibrary.wiley.com/doi/abs/10.1111/tpj.70016, e70016 TPJ-00054-2025.

Supplementary Material

S1. Functional PCA

This appendix provides an intuitive explanation of functional principal component analysis (FPCA) for readers without a quantitative background. The goal is to illustrate, through a simple simulated example, how FPCA decomposes variation across a population of curves into independent modes of variation. We consider curves composed of two distinct components: a smooth, broad parabolic shape and a rapid oscillatory pattern (see Figure S1. These components vary independently across samples.

Using a large set of such simulated curves, we apply FPCA to extract the dominant patterns of variation. Each original curve can then be approximately reconstructed as a combination of a mean curve and weighted contributions from the first few principal components. This decomposition helps clarify how variation is structured across a population and which types of patterns dominate.

Fig. S1 shows five example decompositions. Each row corresponds to one simulated curve, split into its two main functional components. This illustration is meant to serve as a visual reference for understanding the role of FPCA in analyzing biological signals that vary smoothly over a continuous domain.

To make this concept more concrete, consider a plant biology scenario where we monitor the growth of plant roots over time. For each plant, we record the length of its primary root at regular intervals, generating a smooth growth curve. These curves reflect dynamic biological processes, including genetic and environmental influences on growth.

Now suppose we are studying several different genotypes or treatments. Each plant's root grows at its own pace and may exhibit unique features: some may grow rapidly early and then plateau, while others grow steadily or even display fluctuating growth due to stress or environmental factors.

By applying FPCA to this dataset of root growth curves, we can:

i. Summarize the dominant patterns of variation: For instance, the first principal component (PC1) might capture differences in overall growth speed (e.g., fast vs. slow growers), while the second component (PC2) might reflect differences in the timing of growth acceleration (e.g., early vs. late spurts).

ii. Reduce dimensionality: Rather than analyzing hundreds of time points, each curve can be represented compactly by just a few scores (weights) corresponding to its projection onto the first few functional components.

iii. Cluster or classify plants based on growth patterns: FPCA scores can be used to group plants with similar dynamic traits or to distinguish between genotypes or treatments based on how their roots grow over time.

This approach is particularly valuable in the plant phenotyping scenarios covered by Chronoroot, where growth dynamics are critical but can be challenging to summarize with static metrics. FPCA allows us to capture and quantify subtle temporal trends in a principled, interpretable way, even when the curves are complex or noisy.

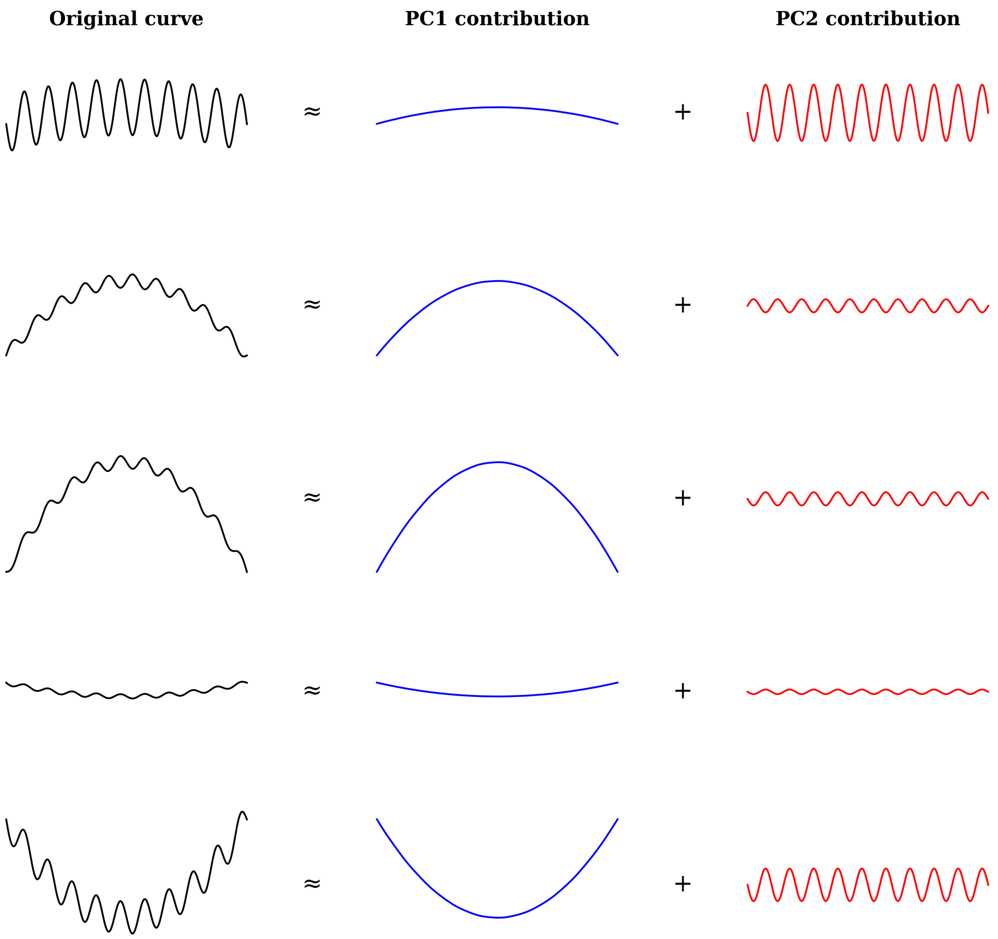

Figure S1. Functional principal component decomposition of simulated curves. Curves were generated as a linear combination of a quadratic function and a high-frequency sine wave, with coefficients drawn independently from normal distributions: $f(x) = a \cdot x^2 + b \cdot \sin(10\pi x)$ with $a \sim \mathcal{N}(0, 1^2)$ and $b \sim \mathcal{N}(0, 0.1^2)$. Each row corresponds to a random instance of such curves. Each curve was centered by subtracting its mean value. Functional PCA was applied to the dataset (using 10,000 randomly sampled curves), and the first two principal components (PCs) were extracted. In each row, the left panel shows the original curve. The middle and right panels show the contributions of the first and second components (PC1 and PC2), respectively. The components are orthogonal and reflect statistically independent sources of variation: the first captures the parabolic shape (due to variation in a), while the second captures the oscillatory pattern (variation in b).

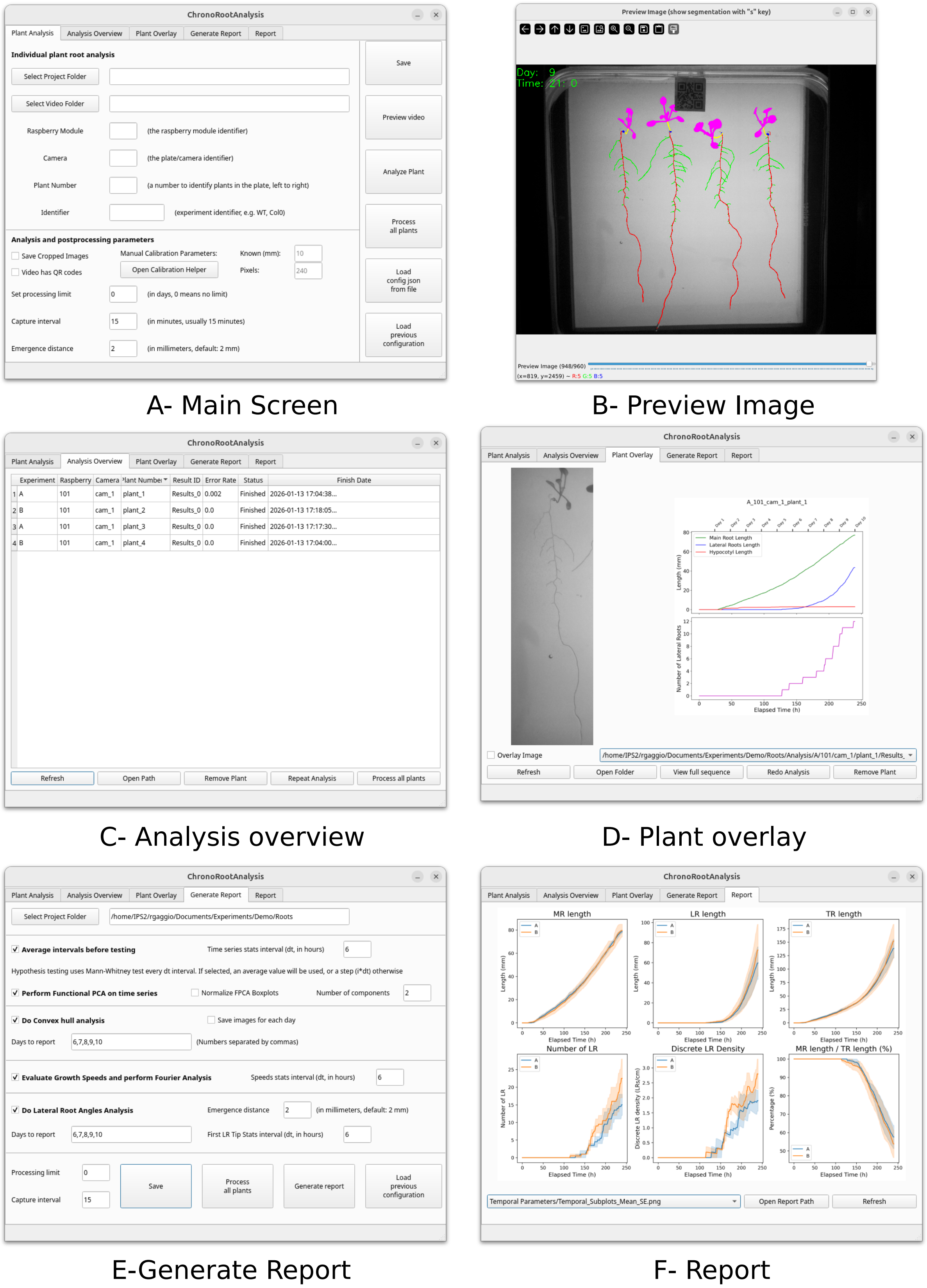

Figure S2. The Standard Root Phenotyping Interface: (A) Main Screen: Plant Analysis tab showing experiment parameters, input/output paths, and processing controls. (B) Preview Image tab with temporal navigation and segmentation toggle for quality assessment. (C) Analysis Overview tab displaying processing completion and error rates. (D) Plant Overlay tab showing individual plant measurements and segmented visualization. (E) Generate Report tab for customizing measurement selection. (F) Report tab displaying finalized architectural analysis results.

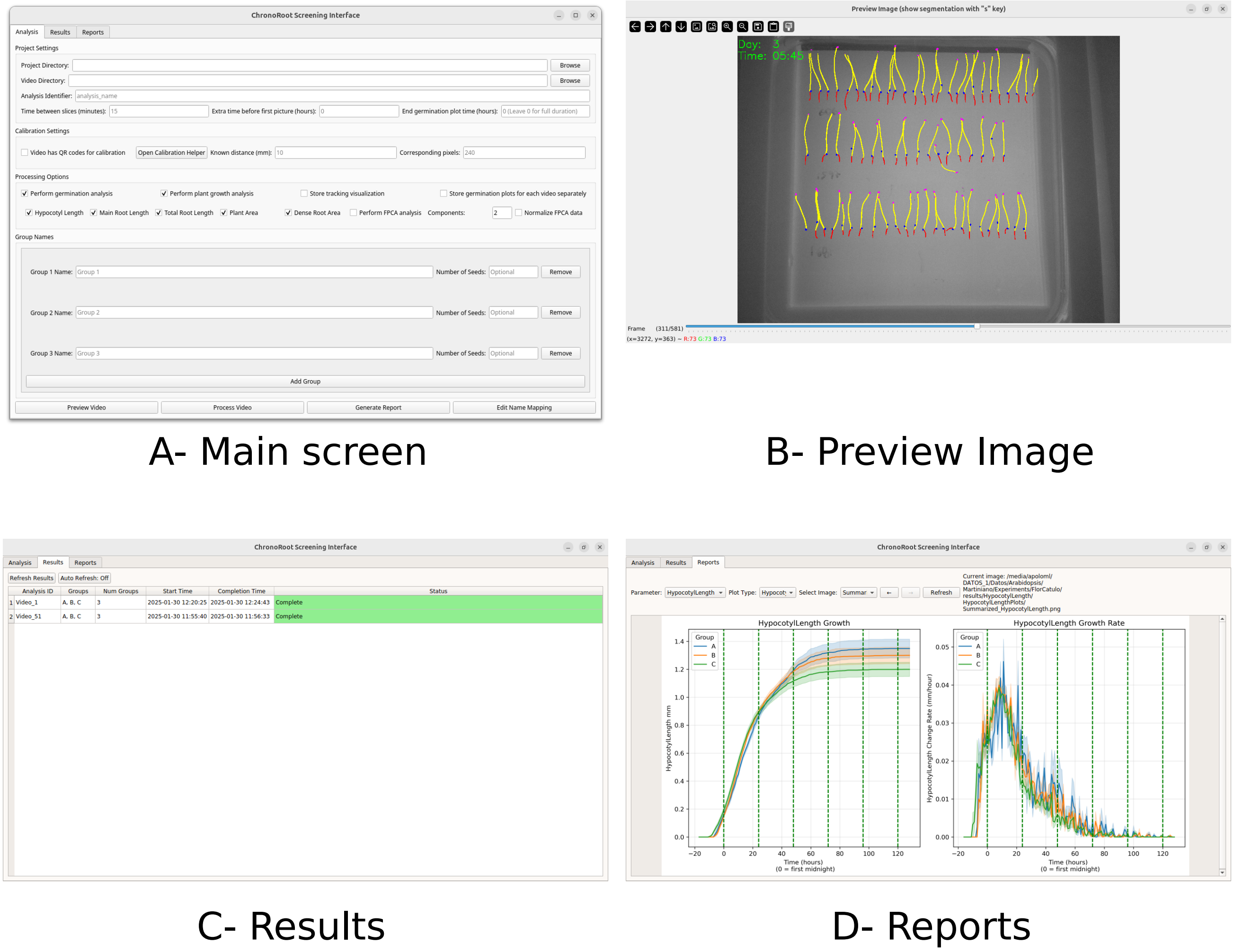

Figure S3. The Screening Interface. (A) Main Screen: Analysis tab with experiment setup, group definition, seed counting, and calibration tools. (B) Preview Image tab providing temporal navigation and segmentation quality assessment. (C) Results tab showing processing status for all plants across experimental groups. (D) Reports tab displaying comparative visualizations and statistical analyses between experimental conditions.

Table S1. Detailed report of segmentation performance across plant organs for *arabidopsis thaliana*.

Configuration	Metric	Complete Root	Main Root	Lateral Root	Seed	Hypocotyl	Leaf	Petiole
Residual	DC	0.800±0.123	0.803±0.122	0.735±0.176	0.686±0.277	0.699±0.230	0.847±0.162	0.758±0.161
	HD	6.658±9.703	6.649±9.895	8.948±11.217	14.811±16.148	10.323±13.220	5.169±7.701	7.160±12.339
	Cp	0.930	0.933	0.895	-	-	-	-
	Cr	0.937	0.939	0.897	-	-	-	-
Residual (Fast)	DC	0.797±0.126	0.800±0.126	0.726±0.183	0.699±0.256	0.695±0.228	0.844±0.163	0.751±0.162
	HD	7.162±10.081	7.243±10.218	9.931±10.178	14.850±15.882	10.051±13.968	5.348±9.297	7.592±13.285
	Cp	0.926	0.929	0.901	-	-	-	-
	Cr	0.935	0.937	0.892	-	-	-	-
Standard	DC	0.798±0.119	0.800±0.119	0.720±0.195	0.696±0.281	0.695±0.240	0.844±0.165	0.758±0.156
	HD	7.765±12.436	7.867±12.513	7.750±9.282	15.792±17.682	8.730±10.412	5.147±7.480	6.666±12.053
	Cp	0.934	0.938	0.890	-	-	-	-
	Cr	0.937	0.938	0.915	-	-	-	-
Standard (Fast)	DC	0.793±0.123	0.796±0.123	0.711±0.203	0.698±0.276	0.687±0.245	0.840±0.166	0.753±0.155
	HD	7.902±12.523	7.986±12.661	8.791±9.679	16.349±18.227	8.916±11.339	7.688±12.846	6.953±11.259
	Cp	0.929	0.933	0.874	-	-	-	-
	Cr	0.936	0.936	0.901	-	-	-	-

Table S2. Detailed report of segmentation performance across plant organs for tomato.

Training	Configuration	Metric	Complete Root	Main Root	Lateral Root	Seed	Hypocotyl	Aerial
Tomato	Standard	DC	0.836±0.178	0.840±0.178	0.643±0.294	0.866±0.202	0.844±0.197	0.778±0.225
		HD	26.470±19.212	26.726±19.700	27.811±19.615	17.744±23.242	7.578±13.767	12.602±16.797
		Cp	0.920±0.133	–	–	–	–	–
		Cr	0.779±0.230	–	–	–	–	–
Tomato	Standard (Fast)	DC	0.805±0.194	0.812±0.188	0.615±0.289	0.884±0.163	0.834±0.201	0.768±0.235
		HD	32.563±19.591	32.483±20.232	33.433±19.507	17.432±22.671	12.784±21.839	19.241±24.454
		Cp	0.910±0.157	–	–	–	–	–
		Cr	0.733±0.253	–	–	–	–	–
Tomato	Residual	DC	0.882±0.154	0.884±0.151	0.651±0.279	0.857±0.191	0.894±0.138	0.807±0.222
		HD	19.676±20.152	15.238±16.119	23.138±20.741	18.005±21.034	6.021±11.986	11.156±17.283
		Cp	0.908±0.153	–	–	–	–	–
		Cr	0.868±0.189	–	–	–	–	–
Tomato	Residual (Fast)	DC	0.871±0.165	0.876±0.159	0.632±0.277	0.839±0.202	0.885±0.147	0.785±0.216
		HD	23.438±20.654	18.147±18.024	24.354±20.547	18.172±20.642	6.290±11.842	13.965±18.417
		Cp	0.904±0.159	–	–	–	–	–
		Cr	0.853±0.197	–	–	–	–	–
Both	Standard	DC	0.853±0.173	0.862±0.162	0.639±0.324	0.885±0.159	0.850±0.186	0.791±0.218
		HD	25.934±20.458	27.324±20.089	23.581±18.204	15.667±21.435	10.579±17.906	15.651±15.909
		Cp	0.914±0.141	–	–	–	–	–
		Cr	0.816±0.223	–	–	–	–	–
Both	Standard (Fast)	DC	0.842±0.181	0.849±0.172	0.638±0.312	0.887±0.150	0.851±0.187	0.771±0.234
		HD	25.137±20.132	26.765±19.903	26.755±18.153	14.647±20.058	8.937±14.857	17.830±17.904
		Cp	0.908±0.156	–	–	–	–	–
		Cr	0.791±0.232	–	–	–	–	–
Both	Residual	DC	0.893±0.162	0.889±0.157	0.673±0.301	0.886±0.166	0.904±0.144	0.854±0.229
		HD	9.943±13.860	11.355±14.100	15.416±16.647	11.604±17.573	5.976±12.757	14.552±23.488
		Cp	0.916±0.138	–	–	–	–	–
		Cr	0.896±0.177	–	–	–	–	–
Both	Residual (Fast)	DC	0.889±0.168	0.883±0.169	0.664±0.288	0.881±0.153	0.895±0.143	0.859±0.196
		HD	10.218±13.685	12.090±13.817	17.317±18.033	12.134±17.901	6.445±13.448	13.454±19.100
		Cp	0.905±0.165	–	–	–	–	–
		Cr	0.899±0.172	–	–	–	–	–

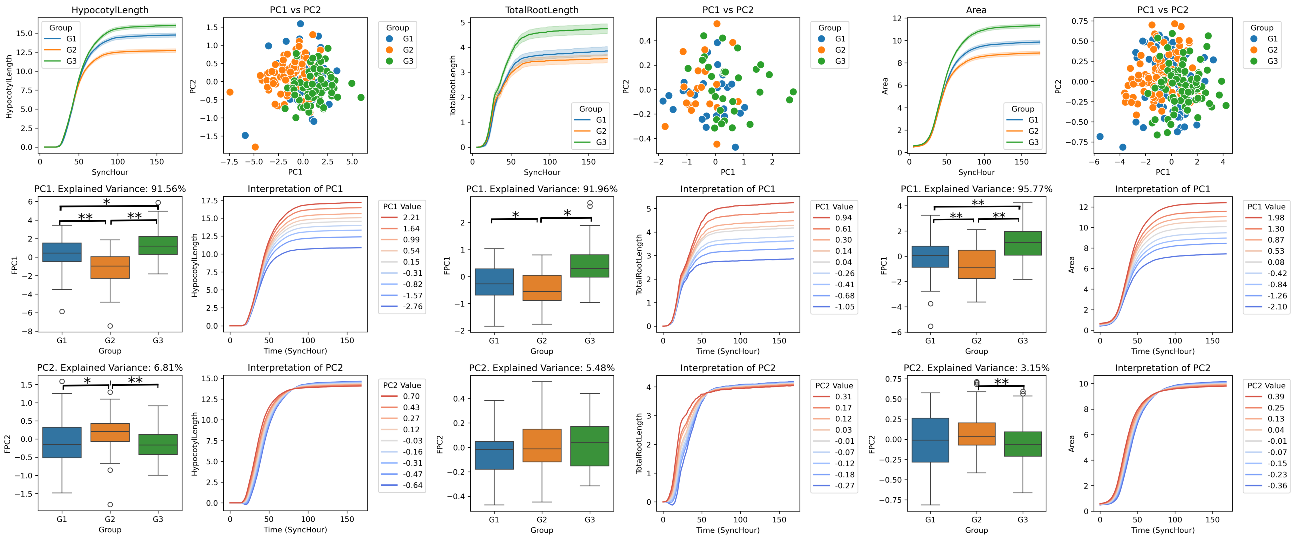

Figure S4. Use Case 3 - FPCA Analysis. To complement the growth dynamics presented in Figure 5-B, we performed FPCA to summarize and compare the developmental trajectories of hypocotyl length, total root length, and projected area across genotypes G1, G2, and G3. The first two principal components accounted for over 90% of the total variance in each trait, capturing the main temporal patterns of growth. For each metric, the top panels show the mean trajectories with standard error bars for each genotype and a scatter plot of PC1 vs PC2. The middle and bottom rows illustrate the distribution of FPCA scores by group for PC1 and PC2, along with visual interpretations of each component. p-values: * < 0.05. ** < 0.001

Manuscript with highlighted changes for easy read
Click here to access/download;Manuscript;Manuscript_Highlighted_changes

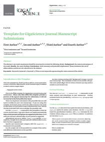

GigaScience, 2026, 1–19

doi: xx.xxxx/xxxx
Manuscript in Preparation
Technical Note

TECHNICAL NOTE

ChronoRoot 2.0: An Open AI-Powered Platform for 2D Temporal Plant Phenotyping

Nicolás Gaggion[1,2,3,4,5,*], Noelia A. Boccardo[1,2], Rodrigo Bonazzola[6], María Florencia Legascue[1,2], María Florencia Mammarella[2], Florencia Sol Rodriguez[1], Federico Emanuel Aballay[1], Florencia Belén Catulo[1], Andana Barrios[4,5], Luciano J. Santoro[1], Franco Accavallo[2], Santiago Nahuel Villarreal[1,2], Leonardo I. Pereyra-Bistrain[4,5], Moussa Benhamed[4,5], Martin Crespi[4,5], Martiniano María Ricardi[1], Ezequiel Petrillo[1], Thomas Blein[4,5], Federico Ariel[1] and Enzo Ferrante[3]

[1]Instituto de Fisiología, Biología Molecular y Neurociencias (IFIBYNE), CONICET-Universidad de Buenos Aires, Argentina and [2]APOLO Biotech, Argentina and [3]Instituto de Ciencias de la Computación, CONICET-Universidad de Buenos Aires, Argentina and [4]Université Paris-Saclay, CNRS, INRAE, Université Evry, Institute of Plant Sciences Paris-Saclay (IPS2), 91190 Gif-sur-Yvette, France and [5]Université Paris Cité, CNRS, INRAE, Institute of Plant Sciences Paris-Saclay (IPS2), 91190 Gif-sur-Yvette, France and [6]Instituto de Investigación en Señales, Sistemas e Inteligencia Computacional sinc(i), CONICET-Universidad Nacional del Litoral, Argentina

[*]Address correspondence to: ngaggion@dc.uba.ar

Abstract

Background: Plant developmental plasticity, particularly in root system architecture, is fundamental to understanding adaptability and agricultural sustainability. Existing automated phenotyping solutions face limitations including binary segmentation approaches, restricted structural analysis capabilities, and text-based interfaces that limit accessibility, with most focusing solely on root structures while overlooking valuable information from simultaneous analysis of multiple plant organs.
Findings: ChronoRoot 2.0 builds upon established low-cost hardware while significantly enhancing software capabilities and usability. The system employs nnUNet architecture for multi-class segmentation, demonstrating significant accuracy improvements while simultaneously tracking six distinct plant structures encompassing root, shoot, and seed components: main root, lateral roots, seed, hypocotyl, leaves, and petiole. This architecture enables easy retraining and incorporation of additional training data without requiring machine learning expertise. The platform introduces dual specialized graphical interfaces: a Standard Interface for detailed architectural analysis with novel gravitropic response parameters, and a Screening Interface enabling high-throughput analysis of multiple plants through automated tracking. Functional Principal Component Analysis integration enables discovery of novel phenotypic parameters through temporal pattern comparison. We demonstrate multi-species analysis, with *Arabidopsis thaliana* and *Solanum lycopersicum*, both morphologically distinct plant species. Three use cases in *Arabidopsis thaliana* and validation with tomato seedlings demonstrate enhanced capabilities: circadian growth pattern characterization, gravitropic response analysis in transgenic plants, and high-throughput etiolation screening across multiple genotypes.
Conclusions: ChronoRoot 2.0 maintains the low-cost, modular hardware advantages of its predecessor while dramatically improving accessibility through intuitive graphical interfaces and expanded analytical capabilities. The open-source platform makes sophisticated temporal plant phenotyping more accessible to researchers without computational expertise.
Software availability: https://chronoroot.github.io

Key words: Plant phenotyping; Root system architecture; Deep learning segmentation; Temporal analysis; High-throughput screening; Open-source software; *Arabidopsis thaliana*; Tomato

1 Introduction

Plants, as sessile organisms, must develop sophisticated adaptive strategies to cope with their immediate environment throughout their lifecycle. This fundamental biological constraint has driven the evolution of remarkable developmental plasticity, enabling plants to complete their life cycles under varying and often suboptimal growth conditions [1]. The root system, being the primary interface between plant and soil, exhibits particularly notable phenotypic plasticity in response to environmental variables [2]. Understanding the dynamics of root system development and its plastic responses has become increasingly critical in the context of climate change and the growing need for sustainable agriculture.

Under controlled conditions, root development is typically observed through images of plants growing vertically on semisolid agarized medium. Root system architecture (RSA) is then characterized through various parameters such as main root length and lateral root density [3]. Several semi-automatic tools assist in root phenotyping at specific time points, yet comprehensive temporal analysis remains technologically challenging [4, 5]. In addition, most existing tools focus exclusively on root structures, overlooking valuable information that could be gained from analyzing other plant organs simultaneously, from an integrative perspective.

The original ChronoRoot [6] system introduced automated temporal phenotyping through a low-cost approach combining off-the-shelf electronics, 3D printed hardware components, and deep learning models. This system demonstrated the potential for automated analysis in plant root developmental studies through high-throughput temporal phenotyping of *Arabidopsis thaliana* RSA. However, its practical application revealed several limitations that restricted its broader adoption in the plant science community.

The binary segmentation approach of the original Chronoroot, while effective for basic root architecture analysis, proved inadequate for capturing the full complexity of plant development, particularly during early growth stages. The requirement for manual seed positioning created a bottleneck in high-throughput analysis, while the text-based interface presented a barrier to adoption by researchers without computational expertise. Additionally, the system's focus on root structures alone meant that valuable information about other plant organs and their developmental relationships was not captured. These limitations highlighted the need for a more comprehensive and accessible solution that could capture the full complexity of plant development while maintaining analytical rigor.

1.1 ChronoRoot 2.0: An Integrated Solution for Plant Phenotyping

Building upon this foundation, we present ChronoRoot 2.0, which significantly expands the capabilities and accessibility of the platform through several key innovations, as illustrated in the comprehensive pipeline shown in Figure 1. The complete workflow begins with the Hardware Module (Fig. 1-A) that combines Raspberry Pi-controlled cameras and infrared LED backlighting. This setup enables consistent Image Acquisition (Fig. 1-B) irrespective of day/night cycles, with a temporal resolution of 15 minutes for extended monitoring of plant development.

At the core of these improvements is an upgraded nnUNet Segmentation module (Fig. 1-C) based on the nnUNet architecture [7], which performs simultaneous multi-class segmentation of six distinct plant structures: main root (class 1), lateral roots (class 2), seed (class 3), hypocotyl (class 4), leaves (class 5) and petiole (class 6). This advancement enables comprehensive tracking of plant development from seed to mature seedling, capturing the intricate relationships between different organs during growth. The choice of nnUNet as the core architecture was motivated by its proven success in medical image segmentation and its ability to self-configure hyperparameters, making it particularly suitable for biologists without extensive machine learning expertise. While the segmentation model is trained to work with both *Arabidopsis thaliana* and *Solanum lycopersicum* (tomato), it can be easily adapted for other species, leveraging the self-configuring capabilities of the nnUNet architecture. This architectural choice, combined with newly developed graphical user interfaces, significantly lowers the barrier to entry for researchers seeking to implement plant phenotyping in their work.

After segmentation, researchers can select between two distinct but complementary interfaces based on their experimental needs. The Standard Root Phenotyping Interface (Fig. 1-D) maintains continuity with the original ChronoRoot design, focusing on detailed RSA analysis of individual plants. This interface provides researchers with tools for precise measurement and analysis of root development patterns through a graph-based representation approach. It maintains the core strengths of the original ChronoRoot system while adding comprehensive visualization capabilities and an intuitive graphical user interface for analyzing basic architecture, growth dynamics, spatial distribution, and newly determined angular measurements.

The new Screening Interface (Fig. 1-E) extends the system's capabilities by enabling automated analysis of multiple plants simultaneously. It incorporates the Simple Online Realtime Tracking (SORT) algorithm[8] for robust plant identification across experimental groups, along with manual calibration tools for standardized measurements. This interface specializes in early development analysis through three dedicated modules: germination analysis, hypocotyl analysis, and plant analysis, enabling researchers to efficiently process and compare multiple experimental conditions.

Both interfaces implement comprehensive quality control mechanisms through real-time feedback via interactive visualization tools. The system introduces several novel analytical capabilities, including automated seed detection and tracking that eliminates the need for manual positioning. Remarkably, we have incorporated here Functional Principal Component Analysis (FPCA) for time series comparison across different groups of plants (e.g. genotypes, treatments, or combinations), enabling the discovery of new data-driven phenotypic parameters that may not be apparent through traditional analysis methods.

Through this dual-interface approach, ChronoRoot 2.0 addresses diverse needs of the plant science community. The Standard Interface provides the precise control and detailed analysis capabilities needed for in-depth root architecture studies of individual plants, while the Screening Interface enables efficient processing of multiple plants and experimental groups, making high-throughput phenotyping accessible to a broader research community.

The remainder of this paper details the technical implementations and validations of each component of Chronoroot 2.0. We begin by describing the enhanced segmentation capabilities and their validation, followed by detailed explanations of the specialized analyses enabled by each interface. We then present the statistical frameworks implemented for both detailed RSA studies and multi-plant screening experiments. Finally, we discuss the system's graphical user interfaces and their role in making advanced phenotyping accessible to the broader plant science community.

Compiled on: January 17, 2026.
Draft manuscript prepared by the author.

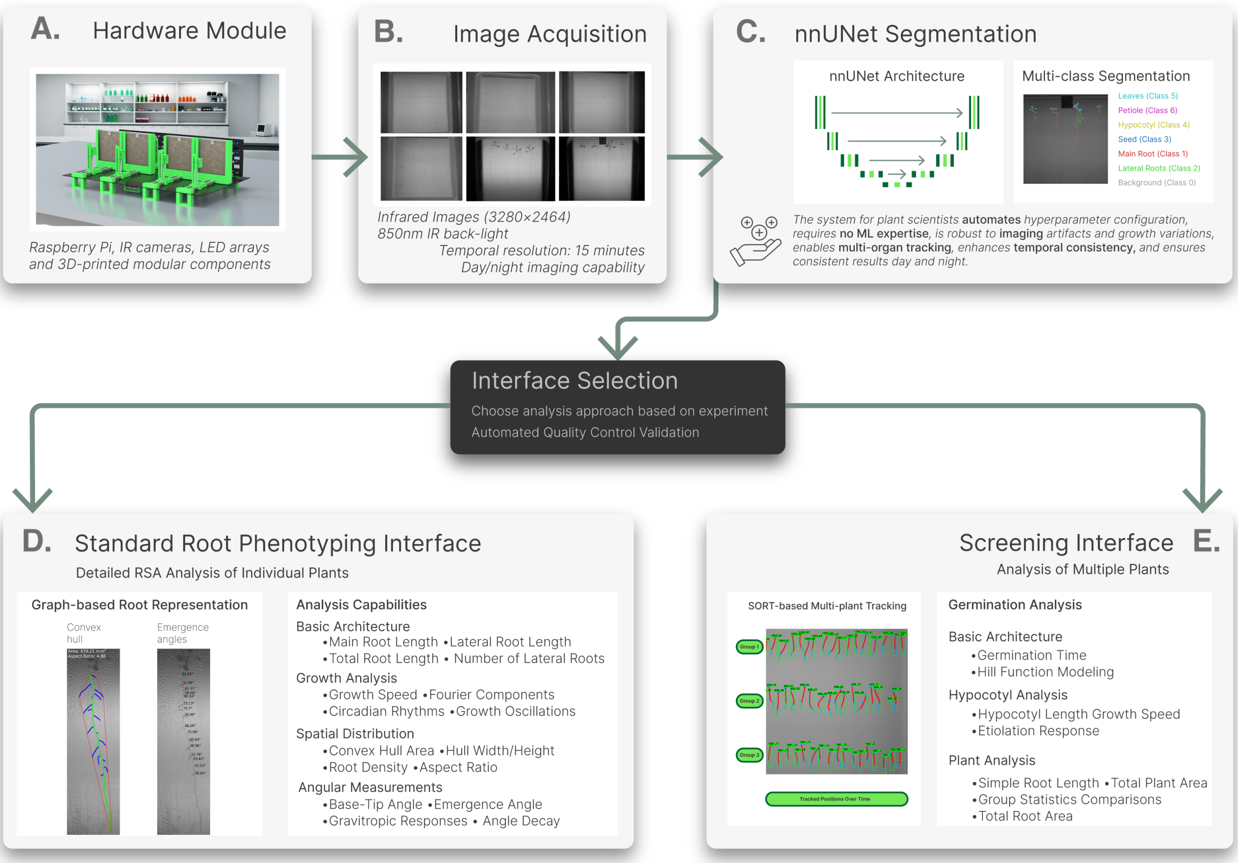

Figure 1. ChronoRoot 2.0: An integrated platform for temporal plant phenotyping. (A) The hardware module combines affordable components for automated imaging in controlled environments. (B) Infrared images are captured continuously, enabling consistent monitoring during both day and night cycles. (C) A multi-class segmentation model based on nnUNet automatically identifies and tracks six plant structures: main root, lateral roots, seed, hypocotyl, leaves, and petiole. The system routes data through an interface selection step, offering two specialized analysis workflows: (D) the Standard Interface for detailed architectural analysis of individual plants, and (E) the Screening Interface for high-throughput experiments involving multiple individuals.

2 Materials and Methods

2.1 System Architecture Overview

ChronoRoot 2.0 is built as a modular software system that integrates hardware control, image processing, and analysis capabilities within a unified framework. The system architecture comprises three main components: a hardware control module for image acquisition using fixed-focus infrared cameras, a deep learning-based segmentation core for multi-class plant structure identification, and two specialized graphical user interfaces designed for different experimental scenarios.

The components operate through a standardized data pipeline: the hardware module captures infrared images at defined intervals, which are then processed by the segmentation core to identify distinct plant structures, before being analyzed through either of the specialized interfaces. The Standard Root Phenotyping Interface enables detailed architectural analysis through graph-based representation, while the Screening Interface facilitates high-throughput analysis of multiple plants through automated plant tracking. This integrated architecture ensures consistent data processing while enabling flexible deployment for various experimental needs.

2.2 Hardware Implementation and Image Acquisition

The ChronoRoot device [6] is an affordable and modular imaging system based on 3D-printed and laser cut pieces combined with off-the-shelf electronics. Each module consists of a Raspberry Pi (v3) embedded computer controlling four fixed-zoom and fixed-focus cameras (RaspiCam v2), and an array of infrared (IR) LED back-light. In between each camera and the corresponding IR array, there is a vertical 12 x 12 cm plate for seedling growth, allowing automatic image acquisition repeatedly along the experiment without any modification or movement of the imaging setup.

The four-plate module is compact (62 x 36 x 20 cm) and can be placed in any standard plant growth chamber. The different parts of the imaging setup (back-light, plate support and camera) can be positioned along a horizontal double-rail to control the field of view of the camera and accurate lighting. In addition, the camera can be moved vertically. ChronoRoot allows image acquisition at a high temporal resolution (a set of pictures every minute). The use of an IR back-light (850 nm) and optional long pass IR filters (> 830 nm) allow acquiring images of the same quality independently from the light conditions required for the experiment, during day and night.

2.3 Deep Learning Segmentation Framework

ChronoRoot 2.0's machine learning capabilities rests on a comprehensive dataset of plant developmental sequences that represents a significant expansion from the original ChronoRoot. The dataset comprises 911 manually annotated images of *Arabidopsis thaliana* and 480 images of tomato, capturing multiple plants across various developmental stages. Expert biologists performed detailed annotation using ITK-SNAP [9], chosen for its precise annotation tools and user-friendly interface.

The annotation process captured seven distinct structural

classes:

- Class 0: Background (non-plant regions)
- Class 1: Main root (primary root axis)
- Class 2: Lateral roots (all secondary roots)
- Class 3: Seed (pre- and post-germination structures)
- Class 4: Hypocotyl (stem region between root-shoot junction and cotyledons)
- Class 5: Leaves (including both cotyledons and true leaves)
- Class 6: Petiole (stalk that attaches the leaf to the stem)

In the tomato dataset, leaves and petiole were annotated as a single combined aerial part class, reflecting species-specific morphological differences.

Image acquisition utilized the ChronoRoot hardware system's Raspberry Pi Noir V2 camera, producing infrared images at 3280 x 2464 resolution (approximately ≈ 0.04 mm per pixel). Images are processed at single channel full native resolution, preserving all structural detail throughout both training and inference. To ensure robust model generalization, the dataset incorporates specimens from various experimental conditions and genetic backgrounds.

The segmentation framework implements the nnUNet architecture [7], selected for its self-configuring capabilities and proven performance in biomedical image analysis. The network dynamically adjusts its depth, width, kernel sizes, stride patterns, normalization schemes, and learning rate schedules based on the training dataset's properties, eliminating the need for extensive hyperparameter tuning. This adaptability is particularly valuable in plant phenotyping contexts, where morphological diversity and varying experimental conditions create unique imaging challenges. Our implementation uses the official nnUNet v2 framework, facilitating straightforward adaptation to new plant species through standard retraining procedures. The framework provides two architectural variants: the standard convolutional nnUNet and the recently introduced nnUNet with residual encoder connections [10], which improves feature learning through skip connections in the encoder pathway. Additionally, the framework supports optional test-time augmentation, where predictions from multiple augmented versions of each image are averaged to improve segmentation robustness, particularly for boundary detection.

2.4 Specialized Processing Pipelines

ChronoRoot 2.0 implements a multi-stage processing pipeline that begins with temporal consistency enhancement of the segmentation outputs. All segmentations produced by the nnUNet undergo a weighted trailing average approach, with special consideration for the multi-class nature of the predictions. This temporal integration strategy significantly improves tracking robustness by incorporating historical structural information alongside new observations. This temporal averaging is selectively applied only to the main root (class 1) and lateral roots (class 2) channels, as these structures require particular stability for accurate tracking. The accumulation is expressed as $a^t = s^t + \alpha a^{t-1}$, where s^t is the current segmentation at time t, a^{t-1} is the accumulated mask up to the previous time step, and α is a weight factor determined by the temporal resolution of the sequence. This approach effectively addresses common imaging challenges in plant phenotyping, such as water droplets, condensation artifacts, or temporary occlusions, providing stable root structure detection throughout developmental timeframes.

Following this temporal processing, the **Standard Root Phenotyping Interface** implements a Region of Interest (ROI)-based analysis approach similar to the one available in the original ChronoRoot system. After initial segmentation of the full image, this interface requires user interaction to define individual ROIs for each plant to be analyzed. This manual ROI selection is crucial for ensuring accurate, independent processing without interference from neighboring specimens. Within each ROI, the system first performs binary mask refinement through morphological operations and connected component analysis. The subsequent skeletonization and graph construction processes operate solely on the refined binary mask within the current ROI, ensuring that the resulting graph structure represents only the selected plant's root system. This approach enables precise measurement of root system architecture parameters and growth patterns while maintaining the ability to analyze multiple plants from the same image sequence over time through sequential processing.

The **Screening Interface** extends the system's capabilities to high-throughput scenarios through automated multi-plant tracking based on the SORT (Simple Online Realtime Tracking) algorithm [8]. The tracking system begins with robust seed detection through contour analysis of segmentation masks and maintains plant identities across frames through a sophisticated combination of Kalman filtering [11] and the Hungarian [12] algorithm. Kalman filtering enables prediction of plant positions in subsequent frames based on their movement patterns, while the Hungarian algorithm optimizes the association between predicted and detected positions, ensuring reliable tracking even in crowded scenes. The interface supports definition of experimental groups for comparative studies and implements comprehensive quality control mechanisms during data postprocessing, including automatic removal of plants that cover or touch each other or exhibit abnormal movement patterns. This automated approach enables simultaneous but simpler analysis of multiple plants while maintaining measurement accuracy, significantly increasing experimental throughput without compromising data quality.

2.5 Analysis Frameworks

The analysis capabilities of ChronoRoot 2.0 comprise three main components: the Standard Root System Architecture Analysis that maintains continuity with the original ChronoRoot system while adding enhanced features, the High-Throughput Screening Analysis that enables efficient processing of multiple plants simultaneously, and a new module implementing Functional Principal Component Analysis (FPCA) that provides sophisticated temporal pattern analysis. In what follows we provide a more detailed description of each module.

2.5.1 *Standard Root System Architecture Analysis*

The Standard Root Phenotyping Interface provides detailed architectural analysis of individual plant root systems through time. This analysis pipeline builds upon the core capabilities of the original ChronoRoot system while introducing new measurements and enhanced processing methods.

The analysis begins with user definition of ROIs, allowing precise selection of individual plants from multi-plant images. Within these ROIs, the system processes segmentation masks through morphological operations and thinning algorithms to obtain skeletal representations of the root system. These skeletons are then analyzed to identify key nodes, which serve as the basis for constructing a graph representation using a depth-first search algorithm [13].

The graph-based approach, combined with temporal tracking of nodes across frames, enables automatic both node and edge classification and measurement of key architectural features. The system distinguishes between main root and lateral root segments through analysis of the graph structure, with special consideration for complex topologies such as loops where lateral roots reconnect with the main root axis. All measurements provided by ChronoRoot 2.0 are summarized in Table 1, organized into five categories: basic architecture, growth analysis, spatial distribution, angular measurements, and high-throughput analysis. The rightmost column indicates which use case (numbered 1-3) demonstrates the practical application of each metric, with detailed results presented in

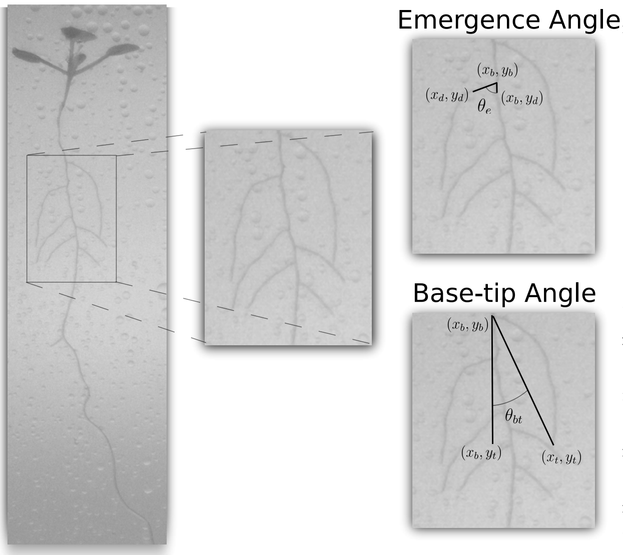

Figure 2. Angular measurements in root system architecture. Illustration of base-tip angle (θ_{bt}) and emergence angle (θ_e) calculations on a *Arabidopsis thaliana* plant showing how these complementary metrics quantify different aspects of lateral root orientation.

the Results section.

Basic architectural parameters capture the fundamental dimensions of the root system through main root length, total lateral root length, and their relationships. Growth dynamics are analyzed through temporal derivatives of these measurements, with special attention to circadian patterns revealed through Fourier analysis of filtered growth speeds. Spatial distribution metrics, computed daily, use convex hull analysis to characterize the overall root system shape and space utilization.

Building upon these established measurements, ChronoRoot 2.0 introduces novel angular parameters that provide detailed insight into lateral root development patterns. These measurements are particularly relevant for quantifying gravitropic responses and directional growth dynamics that are central to plant developmental studies. The measurements leverage both the graph structure, which provides precise identification of lateral root base and tip positions, and the labeled skeleton representation, which enables tracking of root paths for emergence angle calculations.

Two complementary angles are calculated with respect to the vertical axis, where 0 degrees represents perfectly vertical growth. The base-tip angle (θ_{bt}) measures the overall orientation using three reference points: the root base coordinates (x_b, y_b), the root tip coordinates (x_t, y_t), and the vertical projection of the tip (x_b, y_t):

$$\theta_{bt} = \arccos\left(\frac{y_t - y_b}{\sqrt{(x_t - x_b)^2 + (y_t - y_b)^2}}\right) \cdot \frac{180}{\pi} \qquad (1)$$

This measurement captures the terminal orientation of the root after all developmental adjustments have occurred. In contrast, the emergence angle (θ_e) quantifies the initial growth trajectory by measuring the angle at a fixed distance d (default 2 mm) from the base:

$$\theta_e = \arccos\left(\frac{y_d - y_b}{\sqrt{(x_d - x_b)^2 + (y_d - y_b)^2}}\right) \cdot \frac{180}{\pi} \qquad (2)$$

For these novel angular parameters (illustrated in Figure 2), the system maintains temporal consistency through careful tracking of individual root components between frames. This temporal integration is particularly important for lateral roots, whose identities must be preserved across timepoints to enable reliable measurement of architectural changes throughout development.

To facilitate data exchange and integration with the broader plant phenotyping community, all measurements are exported in the Root System Markup Language (RSML) format [14]. This standardized XML-based format stores the complete hierarchical structure of main and lateral roots along with their spatial coordinates and derived metrics, enabling interoperability with other root phenotyping tools.

To prepare the data for final analysis and visualization, all measurements pass through an automated post-processing script that ensures biological and structural consistency across the time series. To account for skeletonization noise and avoid the inclusion of false-positive "spurs" on the root axes, we implemented a structural pruning threshold where a minimum length of 5 pixels ($\approx$ 0.2 mm) is required for a skeleton branch to be preserved prior to graph construction. Additionally, a measurement post-processing script filters out "false starts" and transient misdetections through a temporal verification window: any detected structure is only validated if it persists beyond a 6-hour threshold. Finally, the pipeline enforces a monotonic growth constraint for length measurements, preventing impossible decreases in plant size over time. While these cleaned results are used for the study figures, the raw segmentations, graphs, and original RSML data remain available for researchers who wish to perform their own specific analyses.

2.5.2 *Multiple Plant Screening Analysis*

The Screening Interface is designed for efficient analysis of multiple plants simultaneously. This framework comprises three specialized analysis modules, each optimized for specific aspects of plant development while maintaining multiple plants high-throughput processing capabilities, with their corresponding metrics summarized in Table 1 under Multiple Plant Analysis.

The germination analysis module implements validated approaches from previous germination analysis systems [15] to monitor seed morphology changes and detect emergence events. The module employs the Four-Parameter Hill Function to model germination progression:

$$G(t) = G_0 + \frac{G_{max} \cdot t^n}{t_{50}^n + t^n},$$

where G_0 represents the base germination level, G_{max} is the maximum germination percentage, n denotes the steepness parameter, and t_{50} represents the time to 50% germination. The Time of Maximum Germination Rate (TMGR) is calculated as:

$$TMGR = t_{50} \cdot \left(\frac{n-1}{n+1}\right)^{1/n}.$$

The hypocotyl analysis module incorporates validation steps for reliable measurement in multi-plant scenarios. The system automatically detects physiologically impossible growth rates and artifacts that can occur when different plants touch and their segmentations combine, incorporating biological constraints by forcing non-decreasing length measurements. To ensure full transparency, the platform preserves all raw, unfiltered measurements alongside the processed results. These files are exported in standard formats, allowing researchers to apply custom validation logic or modify the underlying code for specialized needs.

The plant analysis module provides rapid quantification of basic growth parameters through efficient skeletonization techniques. While not as detailed as the graph-based analysis of the Standard Interface, this module extracts fundamental measurements such as main root length, root area, and full plant area, enabling effective high-throughput screening of general growth patterns.

Table 1. Overview of metrics provided by ChronoRoot 2.0. Measurements organized by category: Basic Architecture, Growth Analysis, Spatial Distribution, Angular Measurements, and High-throughput Analysis. The Use Case column indicates which demonstration study (#1: circadian analysis, #2: gravitropic response, #3: etiolation screening) employs each metric.

Category	Metric	Units	Measurement Method	Use Case
Basic Architecture	Main Root (MR) Length	mm	Path length along graph skeleton	1
	Lateral Root (LR) Length	mm	Sum of all LR path lengths	1
	Total Root (TR) Length	mm	MR Length + LR Length	1
	Number of Lateral Roots	count	Unique edges emerging from main root path	1
	Discrete LR Density	LRs/cm	10 * Number of LRs / MR Length	1
	Main Over Total Root	ratio	MR Length / TR Length	1
Growth Analysis*	Growth Speed	mm/h	Temporal derivative of length measurements	1
	Detrended Growth Speeds	mm/h	Raw speeds minus median-filtered signal	1
	Fourier Components	Hz	Fast Fourier Transform of detrended time series	1
Spatial Distribution	Convex Hull Area	mm^2	OpenCV convex hull function	2
	Convex Hull Width	mm	Maximum horizontal extent	2
	Convex Hull Height	mm	Maximum vertical extent	2
	Root Density	mm/mm^2	Root Length / Convex Hull Area	2
	Aspect Ratio	ratio	Height / Width	2
Angular Measurements	Base-Tip Angle	degrees	Angle between vertical and root tip	2
	Emergence Angle	degrees	Angle at 2mm from base	2
Multiple Plant Analysis	Germination Time	hours	Time to radicle emergence	3
	T50	hours	Time to 50% germination	3
	TMGR	hours	Time of maximum germination rate	3
	Final Germination	%	Percentage of germinated seeds	3
	Seed Size	mm^2	Area at experiment start	3
	Hypocotyl Length	mm	Path length of hypocotyl skeleton	3
	Hypocotyl Growth Speed	mm/h	Length difference between timepoints	3
	Total Plant Area	mm^2	Sum of all segmented regions	3
	Simple Root Length	mm	Path length of primary root skeleton	3

*Applicable to any temporal measurement

2.5.3 *Functional Data Analysis of Plant Development*

A major methodological advancement in ChronoRoot 2.0 is the implementation of FPCA for analyzing temporal patterns in plant development. FPCA [16] represents a significant analytical improvement over conventional time-series approaches by treating growth trajectories as continuous functions rather than discrete measurement points.

While traditional plant growth analysis typically relies on point-wise comparisons or summary statistics, which can miss subtle patterns in developmental dynamics, this approach considers the entire growth curve as a functional unit. This enables detection of complex temporal patterns and variations in growth rates that might be overlooked by conventional methods, particularly valuable in plant development studies where the timing and rate of growth can be as biologically relevant as final measurements. For more details and a more graphical explanation of FPCA, we refer the reader to Supplementary Material S1.

FPCA processes temporal measurements through several steps. First, each growth trajectory is converted into a functional data object using monomial basis expansion, providing a continuous representation of the development pattern. The system then performs dimensionality reduction to extract principal component functions that capture the main modes of variation in the data. These functional components are ranked by their explained variance ratio, with typically 2-3 components accounting for over 90% of the observed variation.

This analysis method can be applied to any temporal measurement extracted by either interface, including root lengths, growth rates, and organ areas. Through quantile-based reconstructions and divergent color palettes, the system provides intuitive visualizations of how components modify developmental trajectories, enabling researchers to detect subtle temporal patterns in growth, identify key time points where developmental trajectories diverge between conditions, and quantify complex growth behaviors through a reduced set of interpretable components.

2.6 Software Implementation and User Interface

ChronoRoot 2.0 introduces two dedicated graphical user interfaces developed with Python and PyQt5, replacing the original text-based configuration system. Both interfaces are built upon a shared foundation of scientific computing libraries including NumPy, Pandas, and SciPy for data processing and statistical analysis, OpenCV for image processing, and Matplotlib and Seaborn for visualization.

The Standard Root Phenotyping Interface maintains the core functionality of the original ChronoRoot system while adding modern visualization capabilities. This interface implements a comprehensive analysis pipeline through several interconnected modules (Supplementary Figure S2). The main interface provides tools for experimental configuration, ROI-based plant selection, and real-time visualization of segmentation results. Through an intuitive workflow, users can configure analysis parameters, process individual plants, and generate detailed architectural measurements. The analysis capabilities include convex hull analysis, lateral root angle measurements, growth speed evaluation with Fourier analysis, and detailed statistical testing using Mann-Whitney tests at configurable time intervals. Users can specify particular days for detailed reporting and adjust various measurement parameters such as emergence distance for lateral roots. The interface incorporates quality control through visual feedback systems that allow users to inspect the segmentation results prior to plant selection and manually define the root starting position. Once processed, the software generates growth videos overlaid with the resulting

Table 2. Segmentation performance comparison between original ChronoRoot and ChronoRoot 2.0. The nnUNet implementation outperforms previous models in both accuracy and processing speed in a separated test set (n=55). All nnUNet configurations achieve higher Dice scores than the original models. While test-time augmentation (TTA) shows no significant impact on segmentation overlap (Dice), it substantially improves boundary precision (Hausdorff), reducing error distances by removing spurious segmentations.

Model	Dice	Hausdorff (mm)	Processing Time (s)
Original ChronoRoot			
DSResUNet (Fast)	0.769 ± 0.043	7.25 ± 6.87	~0.5
Ensemble (Accurate)	0.772 ± 0.048	7.21 ± 7.02	~4.5
ChronoRoot 2.0 nnUNet			
Standard	0.809 ± 0.041	6.41 ± 5.39	2.80 ± 0.09
Standard (no TTA)	0.808 ± 0.042	11.08 ± 13.58	0.89 ± 0.07
Residual	0.812 ± 0.038	9.07 ± 9.33	5.34 ± 0.15
Residual (no TTA)	0.815 ± 0.032	13.10 ± 14.18	1.57 ± 0.04

graphs and showcases the measurements, enabling researchers to visually validate the tracking performance for each plant. Problematic individuals can then be discarded or re-analyzed before the system proceeds to the automated generation of comprehensive reports and statistical summaries.

The Screening Interface introduces a streamlined workflow for high-throughput phenotyping experiments (Supplementary Figure S3). The interface guides users through a systematic process from initial calibration to analysis, featuring a dedicated manual calibration tool for precise spatial measurements and an interactive group selection system for defining experimental conditions. Users can define regions of interest corresponding to different treatments or genotypes, and input manual seed counts when needed. The interface implements three specialized analysis modules: germination analysis, hypocotyl development tracking, and basic plant measurements. Real-time visualization tools allow users to monitor segmentation quality, tracking performance, and analysis results as they are generated.

Both interfaces employ multithreading to maintain responsiveness during computationally intensive operations. Quality control mechanisms are integrated throughout the workflows, enabling users to quickly identify and address potential issues. The system generates automated reports featuring graphical summaries and numerical statistics, making experimental results readily available for analysis and publication. The complete codebase and documentation are freely available through our GitHub repository (detailed in the Data Availability section), enabling reproducibility and further development by the community.

3 Results

The performance and capabilities of ChronoRoot 2.0 were evaluated through four key aspects. First, we assessed the core segmentation capabilities, comparing our nnUNet implementation against the original ChronoRoot system in both accuracy and computational efficiency. Second, we validated the system's multi-class detection capabilities, evaluating its performance in simultaneously identifying and tracking six distinct plant structures across both *Arabidopsis thaliana* and tomato. Third, we demonstrated the system's multi-species capability through comprehensive evaluation on both species, showcasing robust performance across morphologically distinct plants. Finally, we demonstrated the software's practical utility through four comprehensive use cases, showcasing its application in both detailed architectural analysis and high-throughput screening scenarios.

Table 3. Multi-class segmentation performance on the *Arabidopsis thaliana* dataset. All model configurations achieve similar results (n=176). Notably, the fast variants provide a significant reduction in processing time with a minor loss in segmentation performance or structural correctness.

Model	Dice	HD	Cp	Cr	Time (s)
Standard	0.763±0.196	8.519±12.500	0.934±0.074	0.937±0.106	2.821
Standard (Fast)	0.758±0.198	9.199±13.312	0.929±0.082	0.936±0.110	0.972
Residual	0.764±0.193	8.415±11.983	0.930±0.104	0.937±0.109	5.300
Residual (Fast)	0.763±0.189	8.743±12.324	0.926±0.109	0.935±0.113	1.630

3.1 Segmentation Performance with nnUNet

We first evaluated the segmentation performance of ChronoRoot 2.0's nnUNet implementation against the original ChronoRoot models using their established dataset (consisting of 339 train images and 55 test images) and metrics (Dice coefficient quantifies the overlap between predicted and ground truth segmentations, while the Hausdorff distance measures the maximum boundary error in millimeters), to validate our architectural improvements. This comparison not only validates the new segmentation approach but also demonstrates backward compatibility with the original system's binary segmentation task, ensuring continuity for existing users while providing enhanced capabilities. The nnUNet implementation showed substantial accuracy gains while maintaining practical processing speeds for high-throughput applications (Table 2).

The original ChronoRoot system offered two operational modes: a rapid DSResUNet implementation (0.5 seconds/image) and a more accurate but slower ensemble method (4.5 seconds/image). While the fast method enabled high-throughput processing, its accuracy (Dice coefficient: 0.769) limited its utility for detailed architectural studies. The ensemble approach achieved marginally better accuracy (Dice: 0.772) but at a significant computational cost.

Regarding ChronoRoot 2.0, we trained two different nnUNet architectural configurations: the standard convolutional architecture and a novel incorporation of a residual encoder architecture [10]. Our implementation allows users to activate or deactivate test-time augmentation (TTA) at inference time, providing a flexible trade-off between processing speed and segmentation quality. All nnUNet configurations substantially outperformed the original ChronoRoot models, achieving Dice coefficients above 0.808 while maintaining practical processing speeds. Disabling TTA reduces inference time by approximately 3-fold (from 2.80 to 0.89 seconds for standard architecture, and from 5.34 to 1.57 seconds for residual), enabling high-throughput processing. Importantly, test-time augmentation showed divergent effects on the two evaluation metrics: TTA had no significant impact on Dice coefficients, comparing architectures with and without TTA revealed nearly identical overlap performance, yet dramatically improved boundary precision as measured by Hausdorff distance, reducing error distances by 40-45%. This improvement stems from TTA's ability to remove spurious segmentations through prediction averaging, which primarily affects boundary outliers rather than overall segmentation overlap.

All training and inference time evaluations were conducted on a standard workstation equipped with an Intel(R) Core(TM) i7-8700 CPU, 64 GB RAM, and an NVIDIA Titan X GPU.

3.1.1 *Multi-Class Segmentation Performance*

Building upon these improvements in binary segmentation, we evaluated the nnUNet's performance in discriminating among six distinct plant structures. This multi-class capability represents a significant advancement over the original system, enabling tracking of multiple plant organs throughout development. The dataset was partitioned, within each of the three major experimental categories (etiolation, germination, and plant root analysis), into training (70%), validation (10%), and test (20%) sets following a video-based splitting strategy to prevent data leakage.

Table 4. Cross-species generalization and training strategy evaluation for the tomato dataset. Residual architectures show better results in Hausdorff Distance (HD) and Correctness (Cr), in the separated test set (n=181). The multi-species training strategy (Both) consistently outperforms training only on tomato data, showing that data diversity improves results across different morphologies.

Training	Configuration	Dice	HD	Cp	Cr	Time (s)
Tomato	Standard	0.815±0.218	19.793±20.590	0.920±0.133	0.779±0.230	2.198
Tomato	Standard (Fast)	0.801±0.220	24.357±22.973	0.910±0.157	0.733±0.253	0.813
Tomato	Residual	0.843±0.200	15.430±18.900	0.908±0.153	0.868±0.189	5.290
Tomato	Residual (Fast)	0.829±0.206	17.295±19.492	0.904±0.159	0.853±0.197	1.586
Both	Standard	0.828±0.212	19.803±20.335	0.914±0.141	0.816±0.223	2.199
Both	Standard (Fast)	0.822±0.214	19.722±19.897	0.908±0.156	0.791±0.232	0.768
Both	Residual	0.863±0.201	11.089±16.640	0.916±0.138	0.896±0.177	5.310
Both	Residual (Fast)	0.858±0.195	11.553±16.174	0.905±0.165	0.899±0.172	1.557

Beyond standard segmentation overlap metrics, successful root system analysis depends critically on preserving key morphological traits. We therefore evaluated the skeletonized root segmentations using completeness and correctness metrics, which directly assess structural fidelity [17]. Completeness measures the extent to which the extracted skeleton retains the original root structure, with higher values indicating fewer missing segments. Correctness evaluates the presence of extraneous or spurious branches in the extracted skeleton, with high values indicating that the segmentation accurately follows the true root architecture without introducing artifacts.

Table 3 presents the overall performance averaged across all plant structures, and the completeness and correctness calculated for the complete root, for each model configuration. All variants achieved similar Dice coefficients, with the standard and residual architectures showing no significant differences between them, but both significantly outperforming their respective fast (non-TTA) variants according to Wilcoxon Pair Ranked Test. Processing times ranged from 1 to 5 seconds per image, with the fast variants providing approximately 3-fold speedup. Detailed per plant organ values are shown in Supplementary Table S1.

3.1.2 *Across species generalization: Tomato.*

To evaluate the generalizability of our approach to other plant species, we trained and tested nnUNet models on a tomato dataset. The data was partitioned by experimental setup, with one complete acquisition (24 plates) reserved for testing, resulting in 299 training images and 181 test images. Note that in the tomato dataset, leaves and petioles were annotated as a single combined aerial part class, reflecting species-specific morphological differences from *Arabidopsis thaliana*.

We evaluated two training strategies: (1) models trained exclusively on tomato data, and (2) models trained on combined tomato and *Arabidopsis thaliana* datasets. For the combined training approach, *Arabidopsis* annotations were preprocessed to match the tomato class structure by merging leaf and petiole classes into a single aerial part category. All four model configurations (Standard, Standard Fast, Residual, and Residual Fast) were evaluated under both training regimes.

The results (Table 4) reveal several important findings. First, residual architectures consistently outperformed standard architectures across all metrics, with particularly notable improvements in Hausdorff distance and correctness measures. Second, incorporating *Arabidopsis* training data significantly enhanced performance across all model configurations, with the combined training strategy yielding the best results. These findings demonstrate both the transferability of knowledge across plant species and the value of diverse training data for robust segmentation performance. Detailed per plant organ values are shown in Supplementary Table S2.

3.2 Demonstration of Software Capabilities Through Use Cases

To validate ChronoRoot 2.0's practical utility across diverse experimental scenarios, we implemented three use cases. The first one examines root system architecture under long day and continuous light condition following the original publication [6], while the second analyzes published data from the transcription factor gene *NF-YA10* over-expressing plants [18]. The final use case demonstrates the high-throughput screening capabilities of the system, on an etiolation experiment. Importantly, all figure subpanels presented in these use cases are direct outputs from ChronoRoot 2.0 and serve as representative examples of the automated reports generated when users analyze their own data or the provided demo datasets. The only modification to these outputs is the addition of asterisks to indicate statistical significance: * for $p<0.05$ and ** for $p<0.001$. While the figures prioritize visual clarity for phenotype comparison, the exact statistical analysis values—including p-values, means, and standard deviations—are provided by the software as accompanying text files within the output folders, requiring no additional analysis beyond what the software automatically generates.

3.2.1 *Use Case 1 - Temporal Dynamics of Root System Architecture: Replication and Extension of ChronoRoot Findings with Fourier and FPCA*

Plant materials: *Arabidopsis thaliana* ecotype Col-0 seeds were surface sterilized and stratified at 4°C for 2d before being grown under long day conditions (16h light, 140$\mu Em^{-2}s^{-1}$/ 8h dark), or continuous light (24h light, 140$\mu Em^{-2}s^{-1}$) at 22°C, on half-strength Murashige and Skoog media (1/2 MS) (Duchefa, Netherlands) with 0.8% plant agar (Duchefa, Netherlands). Four seeds were used per plate.

Root system architecture exhibits complex temporal dynamics that can reveal fundamental aspects of plant adaptation to environmental conditions. Building upon the findings reported in [6], we investigated how different light regimes influence root development patterns, leveraging our enhanced analytical capabilities to uncover subtle temporal variations in growth dynamics.

To validate and extend the findings from ChronoRoot, we replicated its analysis pipeline and incorporated FPCA to further dissect the temporal dynamics of RSA. First, we computed conventional RSA metrics, including main root length, lateral root length, total root length, lateral root density, and the proportion of the main root relative to total root length (Figure 3-A). We then explored root growth dynamics by applying FPCA to the temporal evolution of root length.

The first functional principal component (PC1) captured the primary growth trajectory of roots (Figure 3-B), revealing differences between photoperiod conditions. The second (PC2) showed distinct divergence between long-day and continuous-light conditions, indicating temporal shifts in growth patterns. Following the ChronoRoot methodology, we also analyzed root elongation rates through Fourier Transform (Figure 3-C) to detect underlying oscillatory patterns, identifying both circadian (24-hour) and ultradian (12-hour) rhythms under long-day conditions, which were disrupted under continuous light. Similar analysis of lateral root length (Figure 3-D) showed comparable patterns.

3.2.2 *Use Case 2 - Complete RSA Characterization of different Arabidopsis thaliana genotypes: Area covered, lateral root angles and tip angle decay over time*

Plant materials: All plants used in this study are in Columbia-0 background. pNF-YA10:GFP-NF-YA10miRres (NF-YA10miRres) stable lines were obtained by transforming *Arabidopsis* plants with a construct bearing 2000 bp region upstream of the start codon of NF-YA10 amplified from genomic DNA (promoter region) and the coding sequence (CDS) of *NF-YA10* without miRNA cleavage site amplified from cDNA, thus resisting miR169-mediated post-

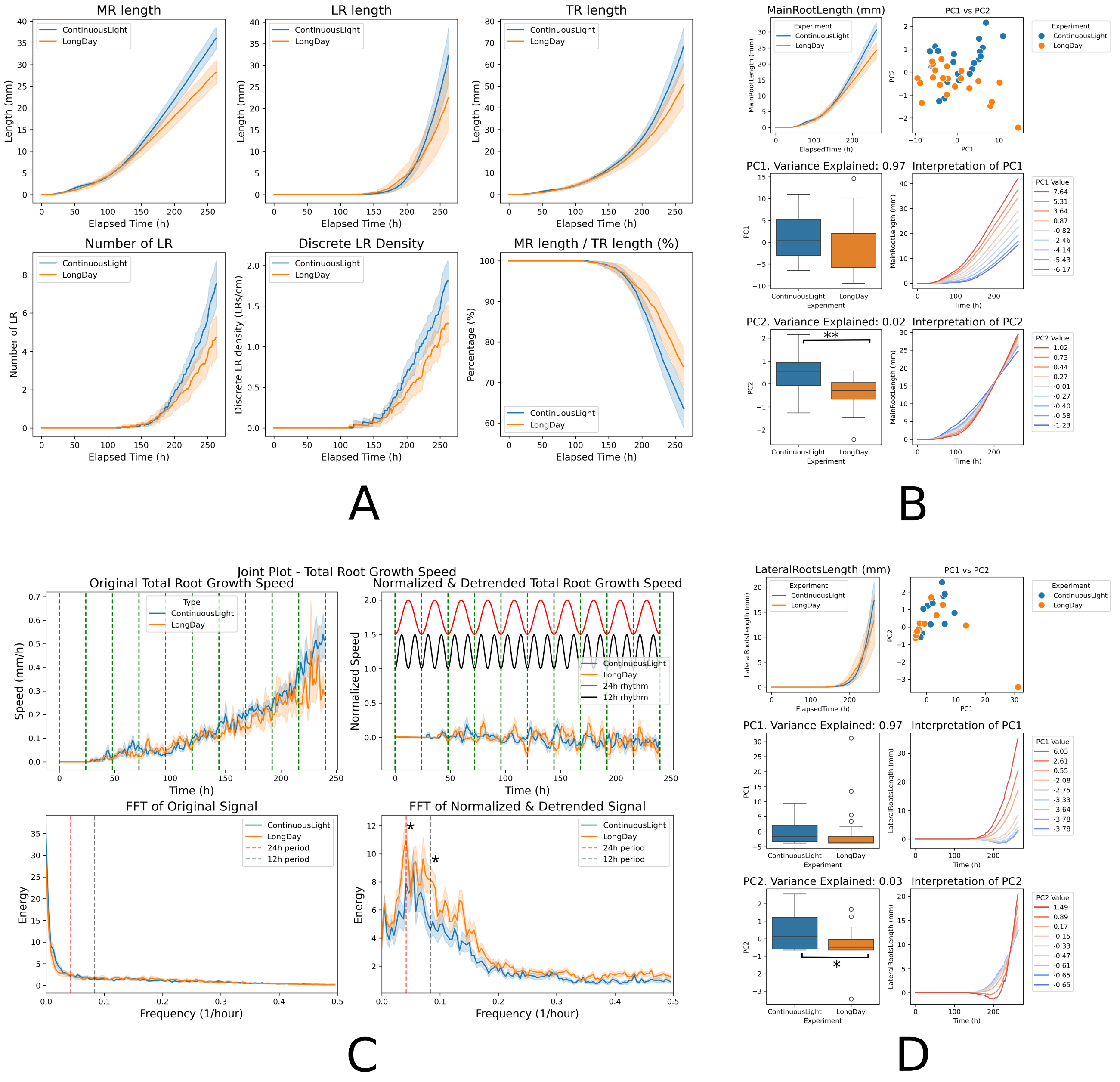

Figure 3. Use Case 1 - *Arabidopsis thaliana* Root system architecture dynamics under different light conditions. Comparison of long-day (16h/8h, blue, n=23) versus continuous light (24h, orange, n=21) shows divergent growth patterns. A - All basic Architectural RSA parameters, B - FPCA analysis of Main Root Length, significative differences found in PC2 (p-value<0.001), C - Fourier transform of Total Root Growth Rate (significative differences found at both 24h and 12h periods, p-value<0.05), D - FPCA analysis of Lateral Root Length, significative differences found in PC2 (p-value<0.05). Error bands: standard error.

transcriptional silencing of *NF-YA10* mRNA. More details were published at [18].

ChronoRoot 2.0's enhanced analytical capabilities revealed distinct architectural patterns between NF-YA10miRes and wild-type Col0 plants. Using the convex hull analysis (Figure 4-A and B), we quantified the overall root system distribution. Qualitative visualization (Figure 4-A) and quantitative metrics (Figure 4-B) showed that NF-YA10miRes plants developed significantly larger convex hull areas, indicating broader root system coverage. Moreover, these plants exhibited higher aspect ratios (height/width), suggesting that lateral roots grew at wider angles from the main root axis rather than clustering around it.

The novel angle measurement capabilities provided detailed insights into these architectural differences. During normal root development, lateral roots typically exhibit gravitropic responses, gradually bending downward after emergence - a phenomenon we term 'angle decay'. Temporal analysis of these lateral root angles (Figure 4-C) showed that NF-YA10miRes plants consistently maintained larger angles compared to wild-type, indicating an altered gravitropic response. The base-tip angle difference progressively increased, reaching a 20° differential after three days of growth. The emergence angles showed similar trends, becoming significantly different from wild-type by day 9. This temporal progression of angular differences suggests that the transcription factor NF-YA10 plays a role in regulating both the initial trajectory and subsequent gravitropic responses of lateral roots.

3.2.3 *Use Case 3 - High-throughput Analysis of Etiolation in Arabidopsis thaliana seedlings*

Plant materials: Lines G1, G2, and G3 were in the Columbia-0 (Col0) background. Seeds were surface sterilized and sown on MS medium supplemented with 1% agar in 120mm-side square petri dishes. To maximize the experimental throughput, up to 100 seeds were placed in each plate in a grid pattern, with 3 rows of 33 seeds.

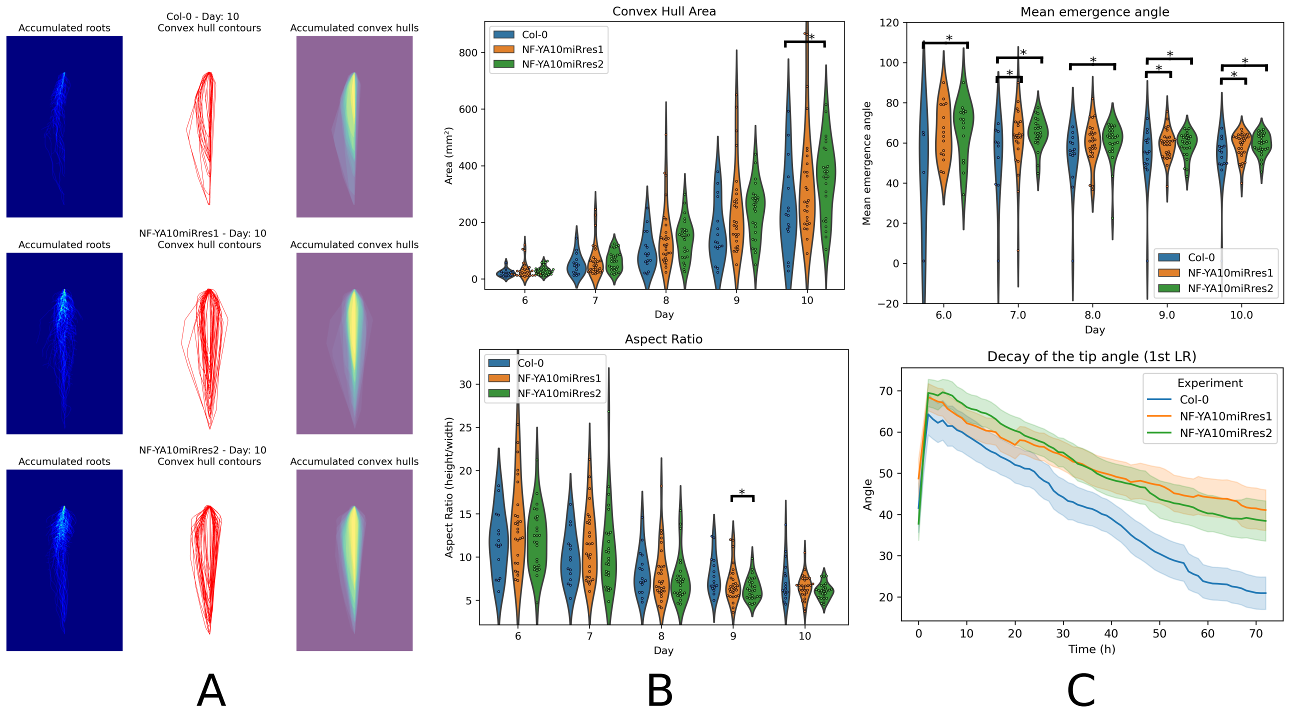

Figure 4. Use Case 2 - Altered root architecture in *Arabidopsis thaliana* NF-YA10miRres plants. A - Qualitative analysis of convex hull showing root system coverage differences between genotypes. B - Quantitative analysis of convex hull metrics (area and aspect ratio) between NF-YA10miRres plants (1 in orange, n=26), 2 in green, n=28) and Col0 controls (blue, n=16). C - Quantitative analysis of average emergence angle and base-tip angle for the first lateral root, demonstrating consistently wider angles in NF-YA10miRres plants compared to Col0. Error bars: standard error.

To demonstrate ChronoRoot 2.0's capabilities for high-throughput phenotyping under specific growth conditions, we conducted an etiolation study across three genotypes. After brief light exposure for germination synchronization, plants were grown in complete darkness for 5 days, with automated infrared imaging every 15 minutes to track development without light interference.

The system's enhanced segmentation algorithm successfully distinguished between hypocotyl and root tissues, enabling precise quantification of both structures' growth dynamics. It was also able to identify smaller structures like the cotyledons (embryonic leaves) and the seed coverage (Figure 5-A). All genotypes exhibited the characteristic etiolation response, with dramatic hypocotyl elongation as seedlings searched for light. Hypocotyl length measurements revealed significant differences in elongation between the three genotypes, with Genotype G3 exhibiting the largest hypocotyl, followed by G1 and then G2 (Figure 5-B). The same differences can be appreciated in the growth rate curves, showing that G3 had more sustained growth, followed by G1 and then G2 (Figure 5-B). Analysis of root system development showed the same temporal pattern as the hypocotyl: growth during the first 3 days followed by complete growth cessation after day 4, marking the exhaustion of seed reserves under dark conditions. This pattern was clearly visible in both main root length progression and area coverage metrics (Figure 5-B).

All genotypes showed the germination of 50% of the seedlings between 13.04 and 13.78 h post light stimulation and did not differ significantly (Figure 5-C). This implied that hypocotyl length differences are due to higher growth rate or sustained growth rather than differences on germination time.

Comparison with manual hypocotyl measurements performed on the same dataset showed no statistically significant differences, demonstrating the robustness of the segmentation and measuring process (Figure 5-D). We compared measures at 36, 48 and 60 hours for the three genotypes.

To further characterize developmental patterns across genotypes, we applied FPCA to the growth trajectories (Supplementary Figure S4). This analysis revealed that over 97% of the variance in hypocotyl length, root length, and total plant area could be explained by just two principal components. The first component (PC1) primarily captured differences in final plant size/length, while the second component (PC2) represented temporal shifts in the growth pattern, similarly to the PC2 in Use Case 1. FPCA scores confirmed the genotype differences observed in the direct measurements, with G3 showing significantly higher PC1 scores for hypocotyl elongation and area development, followed by G1 and then G2, as expected.

3.2.4 *Use Case 4 - Multi-species Capability: Tomato Analysis*

Plant materials: Tomato seeds of cultivar M82 were surface sterilized and sown on MS medium under standard conditions. To accommodate the larger size of tomato seedlings compared to *Arabidopsis*, plant density was reduced to two seeds per plate.

To further demonstrate the species-agnostic design of ChronoRoot 2.0, we applied the complete analysis pipeline to tomato seedlings, which present larger organs, thicker roots, and increased curvature compared to *Arabidopsis*. The nnUNet-based segmentation accurately identified and tracked main roots, lateral roots, and hypocotyls over time in both wild type and mutant plants (Figure 6-A), enabling the extraction of standard architectural and temporal traits without parameter tuning.

Quantitative analysis revealed marked differences between the two genotypes. Spatial descriptors such as accumulated root traces and convex hulls showed that mutant seedlings explored a substantially smaller area than wild type plants (Figure 6-B). Temporal measurements of main root, lateral root, hypocotyl, and total length highlighted an early and persistent reduction in growth in the mutant condition (Figure 6-D). Principal component analysis of the extracted traits captured most of the variance with the first two components and clearly separated wild type and mutant populations, reflecting differences in overall growth magnitude and temporal progression (Figure 6-C).

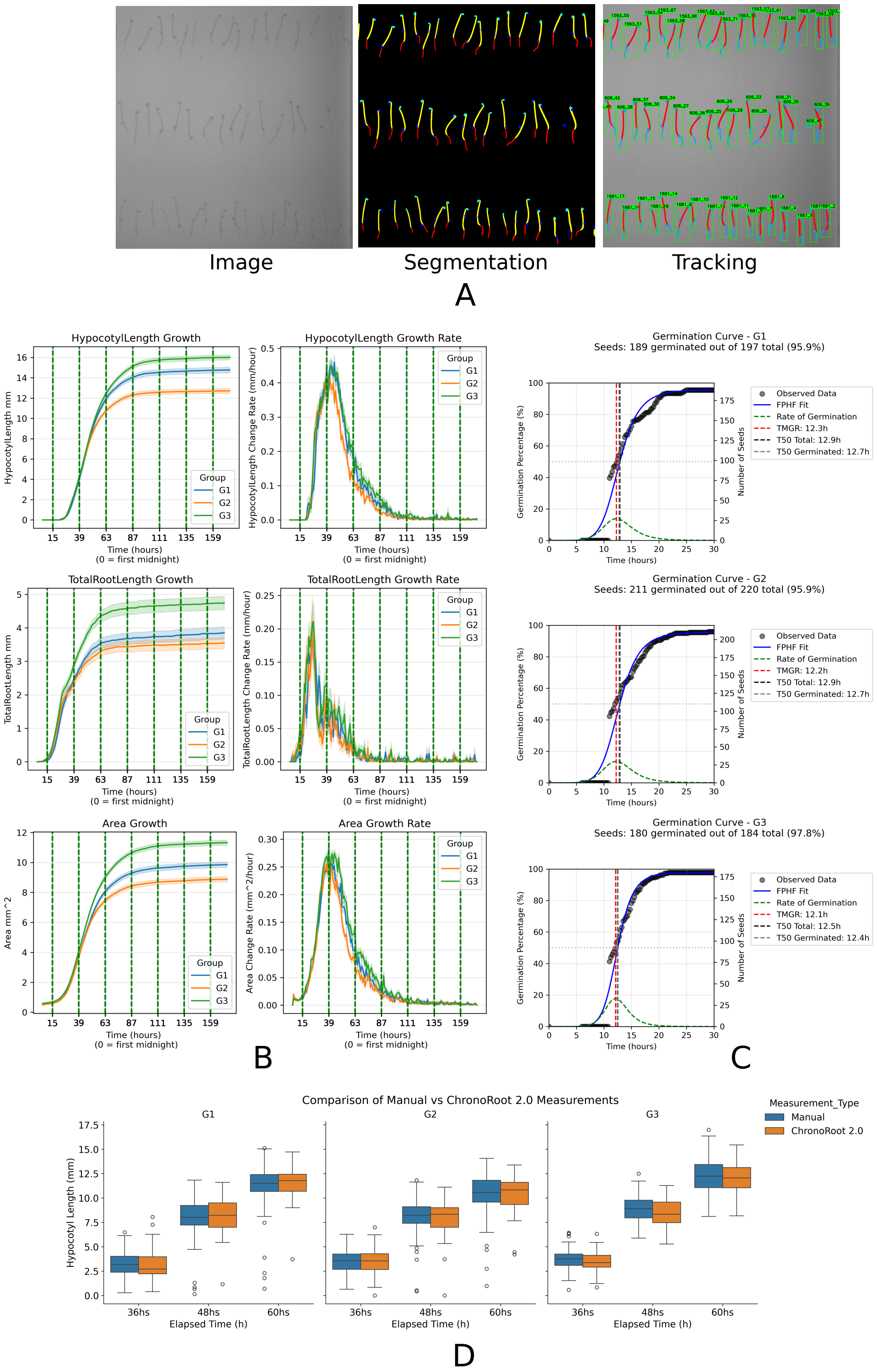

Figure 5. Use Case 3 - High-throughput analysis of *Arabidopsis thaliana* seedling etiolation. A - Representative infrared images showing temporal progression of etiolated seedling development. B - Hypocotyl, root and total area measurements with their corresponding growth rates (G1, blue, n=189. G2, orange, n=211; G3, green, n=180). C - Germination curves showed T50 at approximately 13 hs after light stimulation with no significant differences between the analyzed genotypes. D - Comparison of Manual (blue) and automatic (orange) hypocotyl length determinations at 36, 48 and 60 hours after light stimulation (no significant difference observed). Error bars: standard error.

4 Discussion

ChronoRoot 2.0 provides plant biologists with an integrated solution for analyzing root system development across multiple experimental scales, integrated with additional parameters of the seedling aerial organs. While the artificial nature of 2D growth systems on petri dishes represents an inherent limitation in root architecture studies, our results demonstrate how enhanced measurement capabilities can reveal meaningful biological patterns even within these constraints.

The multi-class segmentation approach addresses a significant challenge in developmental studies by enabling simultaneous analysis of multiple plant structures. Although root growth on agar plates differs from soil conditions, the ability to precisely track both below and above-ground organs provides valuable insights into developmental coordination. The etiolation response study demonstrates

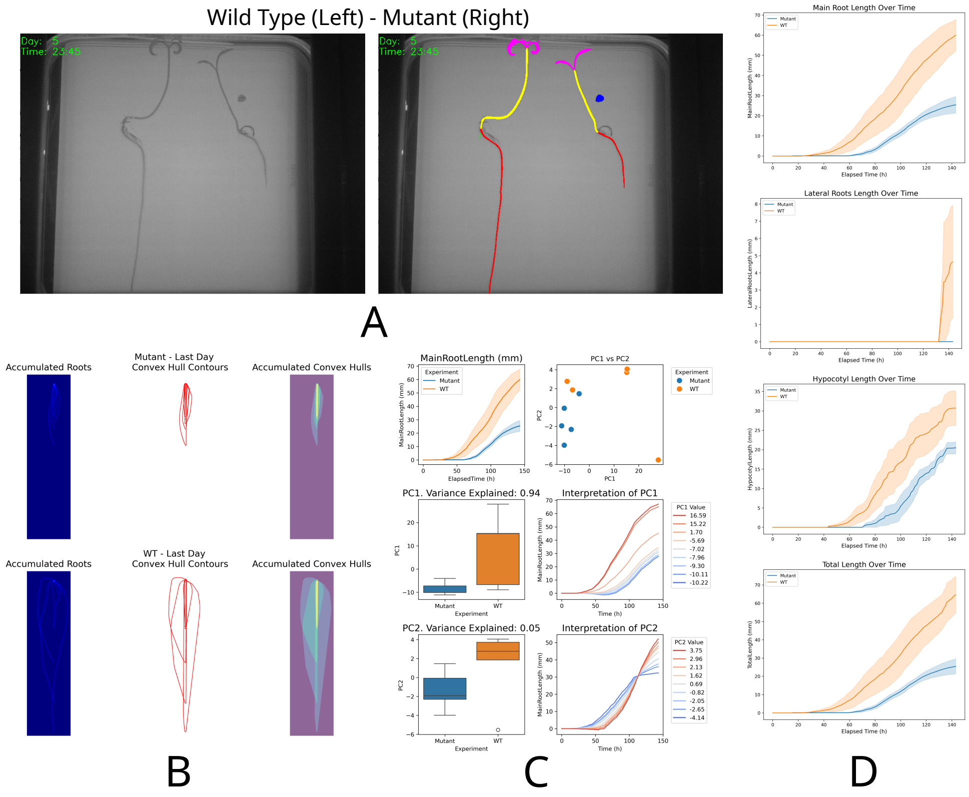

Figure 6. **Use Case 4 - ChronoRoot 2.0 multi-species analysis on tomato seedlings.** A - Representative time-lapse images of wild type (WT, left, n=5) and mutant (right, n=5) tomato seedlings, with nnUNet-based segmentation and organ tracking overlays. B - Spatial characterization of root system architecture showing accumulated root traces, last-day convex hull contours, and occupied growth area for mutant (top) and WT (bottom). C - Multivariate analysis of extracted traits: main root length dynamics, PCA variance explanation, boxplots for PC1 and PC2, and temporal interpretation of principal components. D - Organ-specific temporal growth curves for main root, lateral roots, hypocotyl, and total length, comparing WT and mutant seedlings.

how this capability can reveal resource allocation patterns during early development, with the simultaneous tracking of hypocotyl elongation and root growth providing a more complete understanding of seedling responses to dark conditions. To demonstrate the system's adaptability beyond *Arabidopsis thaliana*, we incorporated a tomato (*Solanum lycopersicum*) dataset. This addition validates that the self-configuring nnUNet core can be effectively retrained to handle the more robust and diverse morphologies of crop species. While applying the system to plants with fundamentally different architectures would require new annotated training data, this framework ensures that the adaptation process remains accessible to researchers without deep machine learning expertise.

While the software architecture is fundamentally modality-agnostic and offers a modular pathway for adaptation to other 2D imaging platforms (such as SPIRO [15]), the platform's robustness is anchored in its integration with our custom hardware. By coupling the software with an open-source, affordable, and easily assembled hardware unit, we ensure high data quality and stable temporal resolution (15-minute intervals) without presenting a significant financial barrier to adoption.

To maximize the utility of these segmentation capabilities within the spatial limits of 12x12 cm plates, we implemented a dual-interface system designed to handle specific experimental constraints. The *Standard Interface* is designed for high-precision architectural tracking of normally 4-6 plants per plate. These experiments are typically limited to 10-14 days for *Arabidopsis* or 5-7 days for tomato, concluding when the main root reaches the plate bottom or grows along the surface invisible to imaging. Conversely, the *Screening Interface* accommodates up to 100 plants per plate but limits analysis to the early developmental window (typically 3-5 days). This mode allows for massive data collection before plant crowding and physical contact prevent reliable segmentation.

The automated angle measurements introduce new possibilities for quantifying gravitropic responses in standardized conditions. While plate-based growth systems impose spatial constraints on root architecture, the precise measurement of emergence angles and their temporal evolution, as demonstrated in the NF-YA10miRres vs. Col0 analysis, enables systematic study of gravitropic regulation. These measurements provide a standardized framework for comparing gravitropic responses across genotypes and conditions, even within the limitations of 2D growth systems.

The temporal analysis capabilities represent a particular strength for understanding dynamic developmental processes. The identification of distinct growth rhythms under different light conditions demonstrates how high-resolution temporal data can reveal patterns that might be missed by endpoint measurements. While circadian patterns in artificial growth conditions may differ from natural environments, the ability to detect and quantify these rhythms provides valuable insights into the temporal organization of plant development. Moreover, the incorporation of FPCA-based analysis facilitates the interpretation of complex temporal signals by reducing their dimensionality, providing a novel and easily-explainable way to quantify dynamic growth patterns.

The analysis frameworks implemented in ChronoRoot 2.0 open new possibilities for understanding plant development, even within the constraints of traditional growth systems. The ability to automatically quantify subtle architectural differences and temporal patterns enables systematic comparison of developmental responses across genotypes and conditions. These capabilities are particularly valuable for studies investigating the genetic and environmental regulation of plant development, where precise quantification of phenotypic differences is essential. Furthermore, high-throughput comprehensive phenotyping emerges as a powerful tool for genome-wide association studies and the identification of key genes participating in plant development.

ChronoRoot 2.0's release as an open-source platform represents

our commitment to accessible, community-driven plant phenotyping tools. While the current implementation provides robust capabilities for analyzing plate-based growth experiments, the modular architecture and comprehensive documentation enable researchers to adapt and extend the system for their specific needs. By releasing both the software and hardware specifications openly, we aim to foster a collaborative community where researchers can share improvements, analytical modules, and experimental protocols. We hope that this approach to open science will not only ensure transparency and reproducibility but also allow the system to evolve alongside the changing needs of the plant biology community.

5 Code and Data Availability

The data supporting the findings of this study consists of three main components:

- The complete source code of ChronoRoot 2.0, including the implementation of all analysis methods described in this paper, is freely available under the GNU General Public License v3.0 at https://github.com/ChronoRoot/ChronoRoot2. This repository contains the full software implementation and comprehensive documentation to set up the system and utilizing the software.
  - Project name: ChronoRoot 2.0
  - Project home page: https://chronoroot.github.io
  - Main Source Code repository: https://github.com/ChronoRoot/ChronoRoot2
  - Operating system(s): Platform independent
  - Programming language: Python
  - Other requirements: Conda, Apptainer, or Docker
  - License: GNU GPL 3.0
- The annotated image dataset used for training and validation contains 911 infrared images of *Arabidopsis thaliana* seedlings and 480 images of tomato with expert annotations for multiclass segmentation. This dataset is publicly available without restrictions at https://huggingface.co/datasets/ngaggion/ChronoRoot2. The dataset includes both raw images and their corresponding multi-class segmentation masks in .nii.gz format, as directly generated in the manual annotations made by our biologists. Scripts to convert to nnUNet's standardized structure for 2D images are also provided within the GitHub repo, generating the correct splits to avoid mixing videos in training, validation and test partitions.
- To facilitate reproducibility and allow users to familiarize themselves with the different analysis modules, we provide four complete demo datasets covering the scenarios presented in this paper. These datasets are available within the apptaniner and Docker images, via the ChronoRoot website, and as Supplementary Material to this manuscript:
  1. **Detailed Root Analysis:** A video for RSA characterization of individual *Arabidopsis* plants.
  2. **Germination Screening:** A video containing hundreds of seeds to test the germination analysis module.
  3. **Etiolation Screening:** A video of seedlings grown in darkness for testing hypocotyl growth rates.
  4. **Tomato Comparison:** A pair of videos of tomato illustrating cross-species capability.
- To facilitate deployment and ensure reproducibility across different computing environments, we provide a pre-configured Docker image at https://hub.docker.com/r/ngaggion/chronoroot. This image includes all necessary dependencies and can be used without any installation requirements beyond Docker itself.

6 Declarations

6.1 List of abbreviations

FPCA: Functional principal component analysis;
GPU: graphical processing unit;
IR: infrared;
LR: lateral root;
MR: main root;
PC: principal component;
ROI: region of interest;
RSA: root system architecture;
RSML: Root System Markup Language;
SORT: Simple online realtime tracking algorithm;
TMGR: Time of maximum germination rate;
TR: total root;

6.2 Ethical Approval

Not applicable.

6.3 Consent for publication

Not applicable.

6.4 Competing Interests

The authors declare that they have no competing interests.

6.5 Funding

AB, NG, TB, MC and FA benefit from the ECOS-SUD Exchange Program (no. A20N05) and the IRP LOCOSYM (CNRS). The FA lab is funded by Agencia I+D+i, ICGEB and AXA Research Fund. AB, MC and TB benefited from the support of the French Agence Nationale de la Recherche (Saclay Plant Sciences-SPS, ANR-17-EUR-0007).

6.6 Author's Contributions

- N. Gaggion led the project implementation, developed all software components, and wrote the manuscript
- R. Bonazzola conceptualized and implemented the Functional Principal Component Analysis methodology
- F. Accavallo contributed to the development and enhancement of the ChronoRoot hardware system
- F. Ariel, A. Barrios, F. Catulo, T. Blein and N. Boccardo conducted the biological experiments analyzed in the study
- F. S. Rodriguez, F. E. Aballay, F. B. Catulo, M. F. Mammarella, M. F. Legascue, and S. N. Villarreal performed manual image annotation for training the Arabidopsis thaliana nnUNet model.
- N. Boccardo and L. Santoro performed manual image annotation for training the tomato nnUNet model.
- L. I. Pereyra-Bistrain and M. Benhamed identified the gene involved in root development, generated the tomato mutant and provided the plant material for Use Case 4.
- F. B. Catulo and M. M. Ricardi performed the validation of the hypocotyl length analyzes
- F. Ariel, E. Petrillo, M. M. Ricardi, T. Blein and M. Crespi provided oversight of all biological experiments and analyses, and evaluated the quality of the generated reports
- E. Ferrante provided oversight of the computational experiments and supervised the software design process
- T. Blein, N. Gaggion, M. Crespi, F. Ariel, and E. Ferrante contributed to the original ChronoRoot concept and its evolution to

version 2.0, and provided manuscript revision

7 Acknowledgements

Not applicable.

8 Supplementary Materials

The following supplementary materials are provided to ensure reproducibility and facilitate adoption of the ChronoRoot 2.0 system.

Text S1 Functional PCA
Provides an intuitive explanation of functional principal component analysis (FPCA) for readers without a quantitative background.

Figure S1 Functional principal component decomposition of simulated curves. Illustrates the fundamental concepts of FPCA through simplified example data.

Figure S2 The Standard Root Phenotyping Interface
Demonstrates the complete workflow of the detailed architectural analysis pipeline through six tabs: Plant Analysis (main screen), Preview Image, Analysis Overview, Plant Overlay, Generate Report, and Report.

Figure S3 The Screening Interface
Illustrates the high-throughput analysis workflow through four tabs: Analysis (main screen), Preview Image, Results, and Reports for efficient multi-plant phenotyping.

Table S1 Detailed report of segmentation performance across plant organs for *Arabidopsis thaliana*.

Table S2 Detailed report of segmentation performance for tomato

Figure S4 Functional PCA applied to etiolation experiment.

Presents FPCA of hypocotyl length, root length, and area growth curves from Use Case 3, showing mean trajectories by genotype, principal component distributions, and visual interpretations of how PC1 and PC2 modulate developmental patterns across genotypes G1, G2, and G3.

References

1. Palmer CM, Bush SM, Maloof JN. Phenotypic and Developmental Plasticity in Plants. In: John Wiley & Sons, Ltd, editor. eLS, vol. 59 Chichester, UK: John Wiley & Sons, Ltd; 2001.p. 1127.
2. Tracy SR, Nagel KA, Postma JA, Fassbender H, Wasson A, Watt M. Crop Improvement from Phenotyping Roots: Highlights Reveal Expanding Opportunities. Trends Plant Sci 2020 Jan;25(1):105–118.
3. Ingram PA, Malamy JE. In: Root System Architecture, vol. 55 of Advances in Botanical Research Elsevier; 2010. p. 75–117.
4. Narisetti N, Henke M, Seiler C, Shi R, Junker A, Altmann T, et al. Semi-automated Root Image Analysis (saRIA). Sci Rep 2019 Dec;9(1):19674.
5. Yasrab R, Atkinson JA, Wells DM, French AP, Pridmore TP, Pound MP. RootNav 2.0: Deep learning for automatic navigation of complex plant root architectures. Gigascience 2019 Nov;8(11).
6. Gaggion N, Ariel F, Daric V, Lambert E, Legendre S, Roulé T, et al. ChronoRoot: High-throughput phenotyping by deep segmentation networks reveals novel temporal parameters of plant root system architecture. GigaScience 2021;10(7):giab052.
7. Isensee F, Jaeger PF, Kohl SA, Petersen J, Maier-Hein KH. nnU-Net: a self-configuring method for deep learning-based biomedical image segmentation. Nature methods 2021;18(2):203–211.
8. Bewley A, Ge Z, Ott L, Ramos F, Upcroft B. Simple online and realtime tracking. In: 2016 IEEE international conference on image processing (ICIP) IEEE; 2016. p. 3464–3468.
9. Yushkevich PA, Gao Y, Gerig G. ITK-SNAP: An interactive tool for semi-automatic segmentation of multi-modality biomedical images. In: 2016 38th annual international conference of the IEEE engineering in medicine and biology society (EMBC) IEEE; 2016. p. 3342–3345.
10. Isensee F, Wald T, Ulrich C, Baumgartner M, Roy S, Maier-Hein K, et al. nnu-net revisited: A call for rigorous validation in 3d medical image segmentation. In: International Conference on Medical Image Computing and Computer-Assisted Intervention Springer; 2024. p. 488–498.
11. Welch G, Bishop G, et al. An introduction to the Kalman filter. University of North Carolina at Chapel Hill 1995;.
12. Kuhn HW. The Hungarian method for the assignment problem. Naval research logistics quarterly 1955;2(1-2):83–97.
13. Cormen TH, Leiserson CE, Rivest RL, Stein C. Introduction To Algorithms. MIT Press; 2001.
14. RootSystemML home page;. http://rootsystemml.github.io/, Accessed: 2025-02-04.
15. Ohlsson JA, Leong JX, Elander PH, Ballhaus F, Holla S, Dauphinee AN, et al. SPIRO–the automated Petri plate imaging platform designed by biologists, for biologists. The Plant Journal 2024;118(2):584–600.
16. Shang HL. A survey of functional principal component analysis. AStA Advances in Statistical Analysis 2014;98:121–142.
17. Youssef R, Ricordeau A, Sevestre-Ghalila S, Benazza-Benyahya A. Evaluation protocol of skeletonization applied to grayscale curvilinear structures. In: 2015 International Conference on Digital Image Computing: Techniques and Applications (DICTA) IEEE; 2015. p. 1–6.
18. Barrios A, Gaggion N, Mansilla N, Blein T, Sorin C, Lucero L, et al. The transcription factor NF-YA10 determines the area explored by Arabidopsis thaliana roots and directly regulates LAZY genes. The Plant Journal 2025;121(5):e70016. https://onlinelibrary.wiley.com/doi/abs/10.1111/tpj.70016, e70016 TPJ-00054-2025.

Supplementary Material

S1. Functional PCA

This appendix provides an intuitive explanation of functional principal component analysis (FPCA) for readers without a quantitative background. The goal is to illustrate, through a simple simulated example, how FPCA decomposes variation across a population of curves into independent modes of variation. We consider curves composed of two distinct components: a smooth, broad parabolic shape and a rapid oscillatory pattern (see Figure S1. These components vary independently across samples.

Using a large set of such simulated curves, we apply FPCA to extract the dominant patterns of variation. Each original curve can then be approximately reconstructed as a combination of a mean curve and weighted contributions from the first few principal components. This decomposition helps clarify how variation is structured across a population and which types of patterns dominate.

Fig. S1 shows five example decompositions. Each row corresponds to one simulated curve, split into its two main functional components. This illustration is meant to serve as a visual reference for understanding the role of FPCA in analyzing biological signals that vary smoothly over a continuous domain.

To make this concept more concrete, consider a plant biology scenario where we monitor the growth of plant roots over time. For each plant, we record the length of its primary root at regular intervals, generating a smooth growth curve. These curves reflect dynamic biological processes, including genetic and environmental influences on growth.

Now suppose we are studying several different genotypes or treatments. Each plant's root grows at its own pace and may exhibit unique features: some may grow rapidly early and then plateau, while others grow steadily or even display fluctuating growth due to stress or environmental factors.

By applying FPCA to this dataset of root growth curves, we can:

i. Summarize the dominant patterns of variation: For instance, the first principal component (PC1) might capture differences in overall growth speed (e.g., fast vs. slow growers), while the second component (PC2) might reflect differences in the timing of growth acceleration (e.g., early vs. late spurts).

ii. Reduce dimensionality: Rather than analyzing hundreds of time points, each curve can be represented compactly by just a few scores (weights) corresponding to its projection onto the first few functional components.

iii. Cluster or classify plants based on growth patterns: FPCA scores can be used to group plants with similar dynamic traits or to distinguish between genotypes or treatments based on how their roots grow over time.

This approach is particularly valuable in the plant phenotyping scenarios covered by Chronoroot, where growth dynamics are critical but can be challenging to summarize with static metrics. FPCA allows us to capture and quantify subtle temporal trends in a principled, interpretable way, even when the curves are complex or noisy.

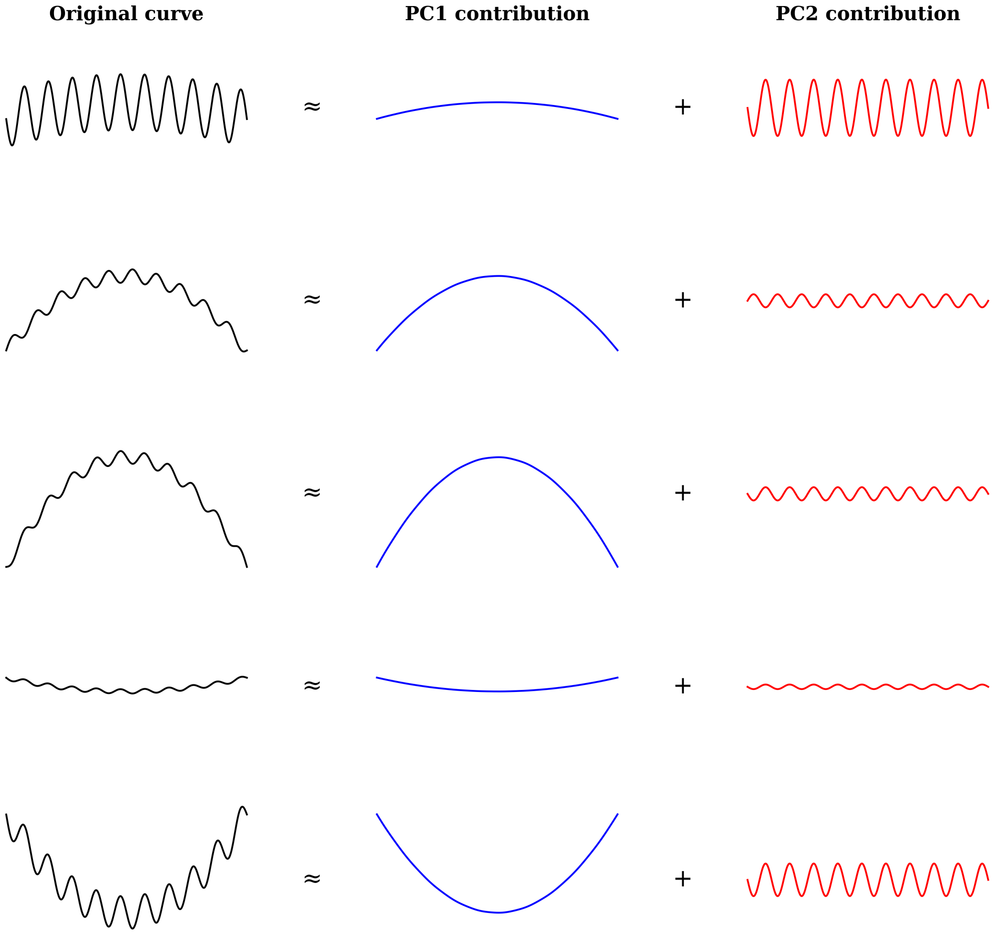

Figure S1. Functional principal component decomposition of simulated curves. Curves were generated as a linear combination of a quadratic function and a high-frequency sine wave, with coefficients drawn independently from normal distributions: $f(x) = a \cdot x^2 + b \cdot \sin(10\pi x)$ with $a \sim \mathcal{N}(0, 1^2)$ and $b \sim \mathcal{N}(0, 0.1^2)$. Each row corresponds to a random instance of such curves. Each curve was centered by subtracting its mean value. Functional PCA was applied to the dataset (using 10,000 randomly sampled curves), and the first two principal components (PCs) were extracted. In each row, the left panel shows the original curve. The middle and right panels show the contributions of the first and second components (PC1 and PC2), respectively. The components are orthogonal and reflect statistically independent sources of variation: the first captures the parabolic shape (due to variation in a), while the second captures the oscillatory pattern (variation in b).

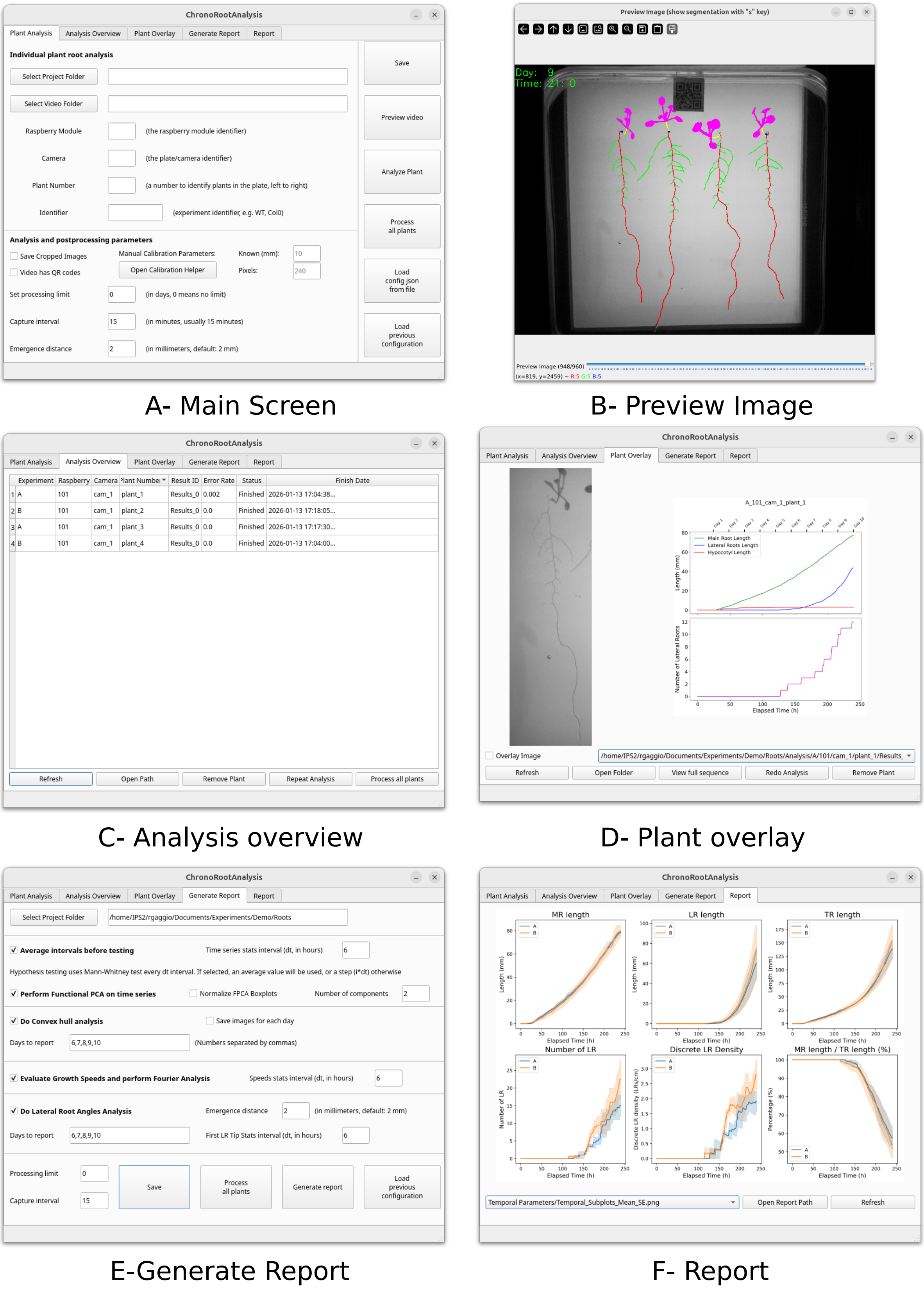

Figure S2. The Standard Root Phenotyping Interface: (A) Main Screen: Plant Analysis tab showing experiment parameters, input/output paths, and processing controls. (B) Preview Image tab with temporal navigation and segmentation toggle for quality assessment. (C) Analysis Overview tab displaying processing completion and error rates. (D) Plant Overlay tab showing individual plant measurements and segmented visualization. (E) Generate Report tab for customizing measurement selection. (F) Report tab displaying finalized architectural analysis results.

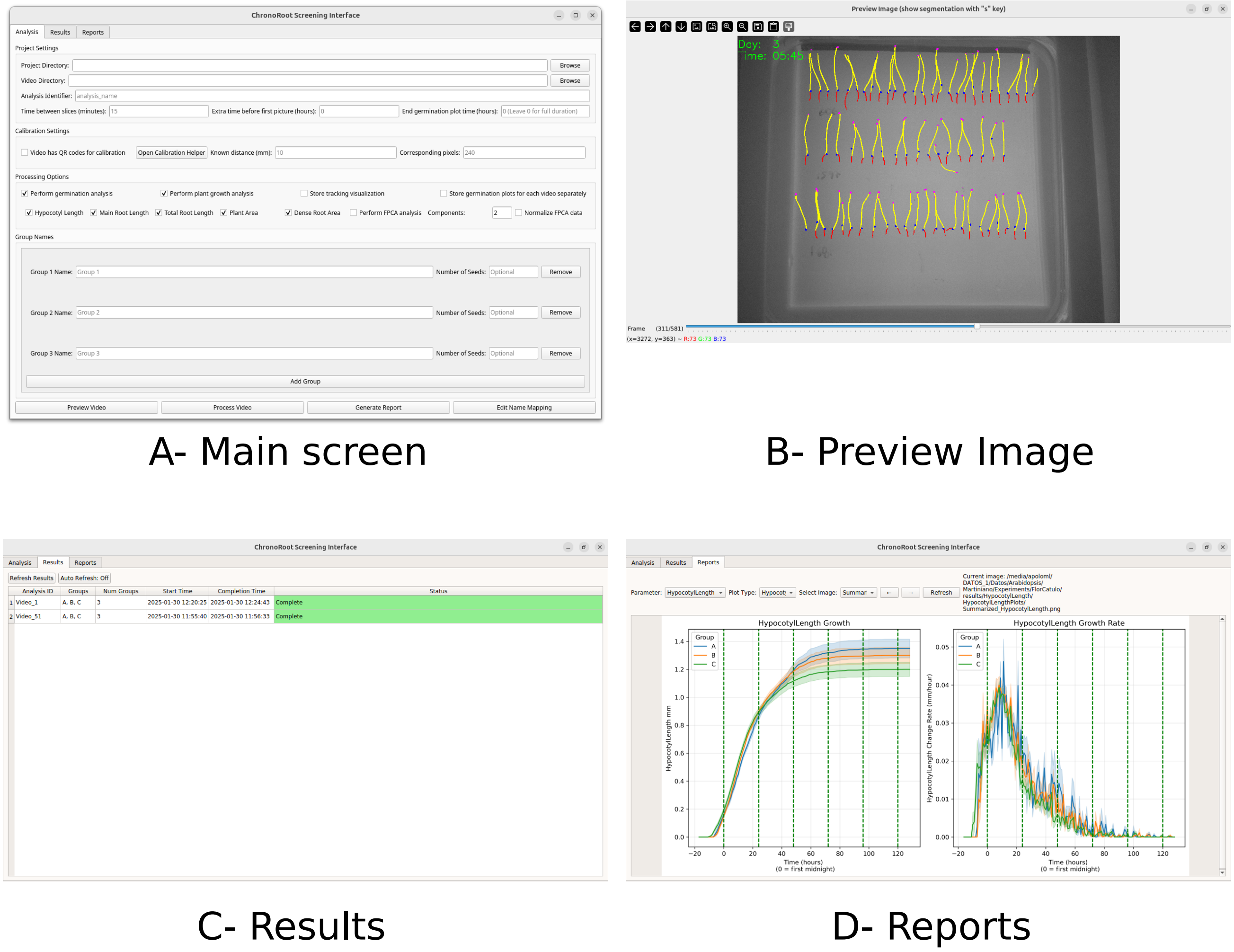

Figure S3. The Screening Interface. (A) Main Screen: Analysis tab with experiment setup, group definition, seed counting, and calibration tools. (B) Preview Image tab providing temporal navigation and segmentation quality assessment. (C) Results tab showing processing status for all plants across experimental groups. (D) Reports tab displaying comparative visualizations and statistical analyses between experimental conditions.

Table S1. Detailed report of segmentation performance across plant organs for *arabidopsis thaliana*.

Configuration	Metric	Complete Root	Main Root	Lateral Root	Seed	Hypocotyl	Leaf	Petiole
Residual	DC	0.800±0.123	0.803±0.122	0.735±0.176	0.686±0.277	0.699±0.230	0.847±0.162	0.758±0.161
	HD	6.658±9.703	6.649±9.895	8.948±11.217	14.811±16.148	10.323±13.220	5.169±7.701	7.160±12.339
	Cp	0.930	0.933	0.895	-	-	-	-
	Cr	0.937	0.939	0.897	-	-	-	-
Residual (Fast)	DC	0.797±0.126	0.800±0.126	0.726±0.183	0.699±0.256	0.695±0.228	0.844±0.163	0.751±0.162
	HD	7.162±10.081	7.243±10.218	9.931±10.178	14.850±15.882	10.051±13.968	5.348±9.297	7.592±13.285
	Cp	0.926	0.929	0.901	-	-	-	-
	Cr	0.935	0.937	0.892	-	-	-	-
Standard	DC	0.798±0.119	0.800±0.119	0.720±0.195	0.696±0.281	0.695±0.240	0.844±0.165	0.758±0.156
	HD	7.765±12.436	7.867±12.513	7.750±9.282	15.792±17.682	8.730±10.412	5.147±7.480	6.666±12.053
	Cp	0.934	0.938	0.890	-	-	-	-
	Cr	0.937	0.938	0.915	-	-	-	-
Standard (Fast)	DC	0.793±0.123	0.796±0.123	0.711±0.203	0.698±0.276	0.687±0.245	0.840±0.166	0.753±0.155
	HD	7.902±12.523	7.986±12.661	8.791±9.679	16.349±18.227	8.916±11.339	7.688±12.846	6.953±11.259
	Cp	0.929	0.933	0.874	-	-	-	-
	Cr	0.936	0.936	0.901	-	-	-	-

Table S2. Detailed report of segmentation performance across plant organs for tomato.

Training	Configuration	Metric	Complete Root	Main Root	Lateral Root	Seed	Hypocotyl	Aerial
Tomato	Standard	DC	0.836±0.178	0.840±0.178	0.643±0.294	0.866±0.202	0.844±0.197	0.778±0.225
		HD	26.470±19.212	26.726±19.700	27.811±19.615	17.744±23.242	7.578±13.767	12.602±16.797
		Cp	0.920±0.133	–	–	–	–	–
		Cr	0.779±0.230	–	–	–	–	–
Tomato	Standard (Fast)	DC	0.805±0.194	0.812±0.188	0.615±0.289	0.884±0.163	0.834±0.201	0.768±0.235
		HD	32.563±19.591	32.483±20.232	33.433±19.507	17.432±22.671	12.784±21.839	19.241±24.454
		Cp	0.910±0.157	–	–	–	–	–
		Cr	0.733±0.253	–	–	–	–	–
Tomato	Residual	DC	0.882±0.154	0.884±0.151	0.651±0.279	0.857±0.191	0.894±0.138	0.807±0.222
		HD	19.676±20.152	15.238±16.119	23.138±20.741	18.005±21.034	6.021±11.986	11.156±17.283
		Cp	0.908±0.153	–	–	–	–	–
		Cr	0.868±0.189	–	–	–	–	–
Tomato	Residual (Fast)	DC	0.871±0.165	0.876±0.159	0.632±0.277	0.839±0.202	0.885±0.147	0.785±0.216
		HD	23.438±20.654	18.147±18.024	24.354±20.547	18.172±20.642	6.290±11.842	13.965±18.417
		Cp	0.904±0.159	–	–	–	–	–
		Cr	0.853±0.197	–	–	–	–	–
Both	Standard	DC	0.853±0.173	0.862±0.162	0.639±0.324	0.885±0.159	0.850±0.186	0.791±0.218
		HD	25.934±20.458	27.324±20.089	23.581±18.204	15.667±21.435	10.579±17.906	15.651±15.909
		Cp	0.914±0.141	–	–	–	–	–
		Cr	0.816±0.223	–	–	–	–	–
Both	Standard (Fast)	DC	0.842±0.181	0.849±0.172	0.638±0.312	0.887±0.150	0.851±0.187	0.771±0.234
		HD	25.137±20.132	26.765±19.903	26.755±18.153	14.647±20.058	8.937±14.857	17.830±17.904
		Cp	0.908±0.156	–	–	–	–	–
		Cr	0.791±0.232	–	–	–	–	–
Both	Residual	DC	0.893±0.162	0.889±0.157	0.673±0.301	0.886±0.166	0.904±0.144	0.854±0.229
		HD	9.943±13.860	11.355±14.100	15.416±16.647	11.604±17.573	5.976±12.757	14.552±23.488
		Cp	0.916±0.138	–	–	–	–	–
		Cr	0.896±0.177	–	–	–	–	–
Both	Residual (Fast)	DC	0.889±0.168	0.883±0.169	0.664±0.288	0.881±0.153	0.895±0.143	0.859±0.196
		HD	10.218±13.685	12.090±13.817	17.317±18.033	12.134±17.901	6.445±13.448	13.454±19.100
		Cp	0.905±0.165	–	–	–	–	–
		Cr	0.899±0.172	–	–	–	–	–

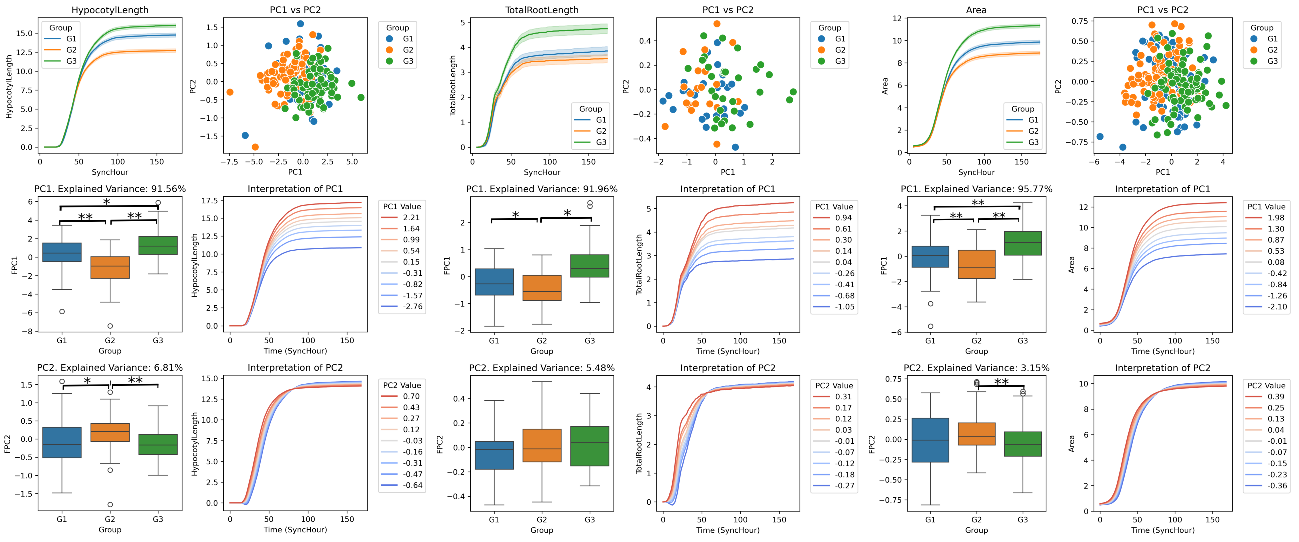

Figure S4. Use Case 3 - FPCA Analysis. To complement the growth dynamics presented in Figure 5-B, we performed FPCA to summarize and compare the developmental trajectories of hypocotyl length, total root length, and projected area across genotypes G1, G2, and G3. The first two principal components accounted for over 90% of the total variance in each trait, capturing the main temporal patterns of growth. For each metric, the top panels show the mean trajectories with standard error bars for each genotype and a scatter plot of PC1 vs PC2. The middle and bottom rows illustrate the distribution of FPCA scores by group for PC1 and PC2, along with visual interpretations of each component. p-values: * < 0.05. ** < 0.001

Click here to access/download
Supplementary Material
answer_reviews_GIGA-D-25-00276.pdf
