# Supplementary material for: ChronoRoot 2.0: an open AI-powered platform for 2D temporal plant phenotyping
Source: Gigascience. 2026 Feb 28;15:giag018. doi: 10.1093/gigascience/giag018 (PMC13042295; doi:10.1093/gigascience/giag018)
Supplement: giag018_Authors_Response_To_Reviewer_Comments_original_submission [file giag018_authors_response_to_reviewer_comments_original_submission.pdf]

Author's Response To Reviewer Comments

Answer to reviewers for Manuscript GIGA-D-25-00276

Dear GigaScience Editor,

We would like to thank you for your positive feedback and the reviewers for their comments. In what follows, we address the reviewer's comment in a point-by-point response. Editorial comments were also addressed in the main manuscript.
Reviewer 1

Comment 1:
Reviewer #1: This manuscript presents ChronoRoot 2.0, a platform that significantly enhances the continuous phenotyping of plant root systems and other organs by integrating a multi-class segmentation model based on nnUNet with low-cost hardware. The article is well-structured, methodologically sound, and presents good results, demonstrating strong engineering integration and technical depth. The following suggestions are provided from a practical perspective:

1. Generalization across species remains unverified
Although the authors claim that the model is easily transferable to other plant species, all current experiments are limited to Arabidopsis thaliana. It is recommended to add other results on at least one non-Arabidopsis species to strengthen the claim of cross-species generalizability.

Response 1:

To address the concern regarding generalization across species, we have incorporated a comprehensive tomato (Solanum lycopersicum) analysis throughout the manuscript. We present a new dataset of 480 annotated images, detailed in Section 2.3 (Lines 162-182), where we also specify the annotation strategy used for this species. A quantitative evaluation of the nnUNet performance on tomato seedlings is provided in Section 3.1.2 (Lines 568-594) and summarized in Table 4. This evaluation includes a comparison of training strategies, demonstrating that a combined dataset approach enhances performance. Furthermore, we added a new Use Case 4 (Lines 740-763) accompanied by Figure 6, which showcases the practical application of the platform for analyzing tomato phenotypic traits.

Comment 2:
2. Segmentation performance is not clearly presented
The multi-class segmentation results are mainly shown through figures. It would be helpful for reading and understanding to show these results with quantitative metrics such as Dice coefficient and Hausdorff distance for each structure in tabular format.

Response 2:

We have integrated comprehensive quantitative evaluations in tabular format. Tables 3 and 4, replacing original Figure 3, present summarized performance metrics for both Arabidopsis and tomato, while Supplementary Tables S1 and S2 were incorporated to provide detailed per-organ metrics, including Dice coefficients, Hausdorff distance, completeness, and correctness.

Comment 3:
3. Limited system generalizability
The proposed platform strictly relies on a specific hardware-software setup (e.g., infrared image input only), which restricts its applicability to other root imaging datasets. In comparison, tools such as RPT: An integrated root phenotyping toolbox have demonstrated the ability to handle more complex root architectures. Even the authors mention that using model fine-tuning is possible to improve its generalization, however nnUNet model is quite sensitivity to the input data distribution, therefore further evaluation on more diverse and complex datasets is recommended.

Response 3:

We appreciate the reviewer's point about generalizability, and agreed with his statement. To clarify: ChronoRoot 2.0 is specifically designed as the software companion to the ChronoRoot hardware platform (Gaggion et al., GigaScience 2021). The system is optimized for infrared images captured under the controlled conditions of this hardware setup, detailed in lines (183-190). While the nnUNet architecture allows adaptation to other species or imaging systems, this would require retraining with appropriately annotated data from those systems. Importantly, to showcase how our model can be adapted to other species, we have now incorporated results for Tomato plants in the revised manuscript. We incorporated all of this in the Discussion, in lines 782-798.

Comment 4:
4. Software setup remains challenging
Although the authors provide a well-structured code and detailed documentation of the software, the installation process involves numerous dependencies and remains difficult for users without a computational background. It is suggested to release an all-in-one installation package (e.g., a standalone executable) to lower the barrier for broader adoption.

Response 4:

We have completely reworked the software architecture to address this concern, transforming the installation from a complex multi-step process into a streamlined, user-friendly experience that mimics a standalone executable:
We fused the three original Conda environment to a single one to allow a simpler installation for advanced users. More importantly for general users, we used this unified architecture to build a "Standalone-Like" Installer:
Apptainer/Singularity Integration: We developed a deployment system using Apptainer that wraps our new Docker base image. Unlike standard Docker containers that can have permission issues, this runs entirely with user-level permissions.
Native Launcher Experience: We created installation scripts for both Linux and Windows (at the moment working for Windows 10 via WSL) that automatically generate system-native Start Menu shortcuts.
One-Click Execution: Users no longer need to open terminals or activate environments. They simply click the "ChronoRoot" icon in their OS menu, and the software launches instantly.
This solution provides the ease of use of a compiled executable while maintaining the scientific reproducibility of a containerized environment. These updates are reflected in the Code and Data Availability section (Lines 873-898), which now includes the updated requirements and a link to the unified container image, and are included in our website at https://chronoroot.github.io/
Reviewer 2

Comment 1:
Reviewer #2: In their manuscript entitled "ChronoRoot 2.0: An Open AI-Powered Platform for 2D Temporal Plant Phenotyping", the authors present an updated version of their previously published framework called ChronoRoot, a tool aimed at the segmentation and quantification of morphological features of plants, in particular their root system. New features have been added, the major one being a graphical interface developed to lower the computational barrier that command lines interfaces constitute for researchers without a bioinformatics background. The segmentation performances have been improved using a deep learning method based on the nnUNet architecture, which, according to authors, makes it easier to adapt to other plant species than A. thaliana. The framework can measure a large variety of morphological parameters of plants such as the length of main/lateral roots, the convex hull area, the emergence angle of roots and can also segment and classify up to 6 organs of
plants (including leaves). Temporal analysis through Fourier analysis is available, as well as functional principal component analysis (fPCA), that is used by authors to discover hidden features of plants growth. Through three use cases, authors highlight the different functionalities of ChronoRoot 2.0, showing the

improvements compared to the first version of the framework.
Overall, the manuscript is well written and the logic underlying the study is quite easy to follow. The metrics used and analytical methods are relevant given the objectives of ChronoRoot. However, the installation of the software (through Docker), despite being extensively described by authors in the related GitHub, is far from being easy, and I could not run it correctly even after a few hours of investigations. As a Windows user, I had to adapt the commands and find "tricks" to finally install the software, I guess that a Linux user would have less issues for the installation, but as the tool, with its GUI, is targeted to biologists without a computational background, the use of Docker is already a barrier; so with additional issues coming from a non-UNIX OS, I'm afraid that the target is slightly missed: users able to install ChronoRoot 2.0 are likely the ones with computational background, and then do not need a GUI.
While authors provided an extensive documentation for the installation, there are no guidelines or tutorial for the use of ChronoRoot itself; I am then lost when trying to use any of the three components (standard, screening or segmentation tools); there are no indications of the format required for the videos, the filenames, folders architecture, parameters, etc. A step-by-step tutorial with toy data for each components (standard, screening, segmentation) would be more than needed in order to help users learn how to use the framework (or simply check that the installation is correct; currently, I couldn't say if it's not running on my computer because of an incorrect installation or me not knowing how to correctly use the software…)
While I recognize the value of Docker for long-term maintenance and version compatibility, I think this still requires basic computational skills that will discourage most of the targeted users. A standalone executable version (ideally three versions for Windows, Linux or Mac) would be really great to have in addition to the Docker solution, so it would make it easier for beginners to adopt ChronoRoot as part of their workflow.
In summary, I'm convinced that ChronoRoot 2.0 with its new GUI and improved morphometrics tools, would be a great framework for the plant biologists community, and while I haven't been able to run the software myself, I am confident that it actually performs the features described in the associated manuscript. But I think authors should work to improve the accessibility of their tool before the manuscript can be accepted.
The following is a detailed list of my comments, from the major to minor comments:

[MAJOR]

1 - Clear tutorial missing on how to use the software (standard, screening and segmentation components). It would be great to have a doc for each component associated with example data, which users can download in order to reproduce the results shown in the tutorial.

Response 1:

We have addressed this by embedding Demo Data directly into our new container images and also accessible via direct download. By unifying our environment, we were able to package a standardized dataset that is instantly available to every user upon installation. We have added comprehensive, step-by-step tutorials to the new ChronoRoot website (https://chronoroot.github.io/) that utilize this specific data. This ensures that every user, regardless of their operating system, can immediately verify their installation and learn the workflow by reproducing the exact results shown in the documentation, without needing to download external files or configure paths manually.

Comment 2:
2 - A standalone executable version of ChronoRoot 2.0 would be great to have (especially for non-UNIX users).

Response 2:

We have effectively implemented this suggestion by completely re-engineering our Windows support to mimic a standalone executable. We recognized that asking biologists to routinely manage Docker commands or terminals was a significant barrier.
To solve this, we performed a complete codebase refactor, unifying our previous multi-environment structure into a single Conda environment, which allowed us to develop a novel Windows Installer (at the

moment for Windows 10). This installer bridges the gap between Windows and Linux containers with the following workflow:
One-Time Setup: The user is required to open WSL2 (Windows Subsystem for Linux) only once to run a single installation command (wget and bash installer_windows.sh that is a simple copy/paste in WSL2 terminal). This script handles all complexity: it can either install the lightweight Apptainer runtime, pull our unified container image, and set up the environment, or directly install the conda environment if a installation of Conda is available.
Start Menu Integration: Crucially, the installer automatically generates Windows Desktop and Start Menu shortcuts (.lnk files) that point to the internal Linux container.
Invisible Complexity: Once installed, the user never needs to touch the terminal again. Clicking the "ChronoRoot" icon transparently handles the WSL invocation and container launching in the background.
To the user, ChronoRoot 2.0 now behaves exactly like a native standalone executable: there is no need to type commands, manage Docker daemons, or handle file permissions during daily use. This provides the requested simplicity while preserving the scientific robustness of a containerized environment. These updates are reflected in the Code and Data Availability section (Lines 873-898), which now includes the updated requirements and a link to the unified container image.
Comment 3:
3 - ChronoRoot 2.0, with the nnUNet architecture is said to be easily adaptable to diverse plant species analysis; but authors never showed this claim on other species than A. thaliana. It would be great to test the segmentation and morphometrics measurements on other publicly available datasets with other species than A. thaliana; this would prove the claim and also highlight the interoperability of ChronoRoot (which is not restricted to ChronoRoot hardware setup).

Response 3:

We have incorporated comprehensive tomato (Solanum lycopersicum) analysis throughout the manuscript (Section 3.1.2 in lines 568-594, Tables 4 and S2, and Use Case 4 in lines 740-763), demonstrating model retraining capability across morphologically distinct species.
However, we wish to clarify an important design consideration: ChronoRoot 2.0 is specifically designed as the software companion to the ChronoRoot hardware platform (Gaggion et al., GigaScience 2021). We do not claim plug-and-play interoperability with arbitrary imaging setups or publicly available datasets from other systems. The system is optimized for infrared images captured under the controlled conditions of this hardware setup.
Testing on external public datasets would require those images to: (1) be captured with similar infrared imaging, (2) have comparable resolution and lighting conditions, (3) show plants in similar growth orientations. Most public root datasets use visible light, different resolutions, or 3D imaging systems, making them incompatible with our trained models without retraining. Our tomato example demonstrates that such retraining is feasible when appropriately annotated data from compatible imaging systems are available, but this is distinct from plug-and-play application to arbitrary external datasets. We incorporated this into the discussion between lines 782-798.

Comment 4:
4 - Similarly, authors compared the segmentation performance of ChronoRoot 2.0 versus its first version; but as other segmentation tools exist, it would be great to compare ChronoRoot 2.0 to the most recent ones, as it's a critical parameter when choosing between available tools.

Response 4:

To improve our segmentation performance analysis, we have expanded the evaluation to include multiple nnUNet architectural variants (Lines 198-208, 513-533). Tables 2-4 and S1-S2 present comprehensive comparisons between the standard nnUNet architecture and the recently introduced nnUNet with residual encoder connections (nnUNet-ResEnc, Isensee et al. 2024), representing the current state-of-the-art in the nnUNet family. Additionally, we evaluate both configurations with and without test-time augmentation (TTA), providing users with multiple accuracy-speed trade-off options.

Regarding comparison with other root segmentation tools: most existing tools (e.g., RootNav, DIRT,

SmartRoot) are designed for different imaging modalities (visible light, X-ray CT, or endpoint analysis) or focus solely on binary root/background segmentation. ChronoRoot 2.0 addresses a distinct problem—multi-class segmentation of six plant structures in infrared temporal sequences—making direct quantitative comparison inappropriate. Our comparison with the original ChronoRoot (which addresses the same imaging modality and experimental design) provides the most relevant performance benchmark, while the nnUNet architectural comparison demonstrates we employ current state-of-the-art segmentation methodology.

Comment 5:
5 - I don't understand why users need to manually define the ROI when using the standard interface, while it is automatically detected with the screening interface; similarly, the segmentation methods are different between these two interfaces, the standard one being more accurate than the screening one (as far as I understand the manuscript, since I couldn't test it myself). It would be great to have the choice both for the ROI settings (it could be an automatic detection, and users could then manually edit the ROI if needed) and for the segmentation method (users could prefer using the screening interface with accurate segmentation, even if it's more computationally expensive).

Response 5:
This is an intentional design reflecting fundamentally different experimental scales and objectives. The Standard Interface targets detailed root architecture analysis of individual plants, typically 4-6 plants per plate that can develop for up to two weeks without crossing. Manual ROI definition serves dual purposes: (1) allowing researchers to assign genotype/treatment labels to specific individuals (we are mixing the genotypes inside the plate in random positions), and (2) providing a quality control checkpoint where biologists verify plant identity and suitability before analysis. This "one ROI equals one plant" approach enables tracking complex root architectures over extended periods where precise individual identity is paramount.
The Screening Interface targets high-throughput experiments with up to 100 plants per plate. At this density, plants can only be measured for a few days before crowding/overlap make individual tracking impossible, and where plant structure is far simpler, allowing an easy automatic detection of the plant. Here, ROIs define groups of plants sharing the same genotype/treatment, not individuals. Within each group ROI, the SORT algorithm automatically detects and tracks multiple plants. This design reflects that high-density screening sacrifices long-term individual tracking for increased sample size during the brief early developmental window before crowding.
Both interfaces use identical nnUNet segmentation at their core—there is no "more accurate" versus "less accurate" segmentation method. The fundamental difference is analytical scope: Standard Interface performs deep architectural analysis of few individuals over weeks, while Screening Interface performs rapid comparative analysis of many individuals over days.
We incorporated this in Discussion lines 799-810
Comment 6:
[MINOR]
1 - (page 4; section "Specialized Processing Pipelines") "This approach effectively addresses common imaging challenges in plant phenotyping, such as water droplets, condensation artifacts, or temporary occlusions, providing stable root structure detection throughout developmental timeframes.": a reference is missing.
In a similar frame, how does nnUNet perform against imaging artefacts?

Response 6:

Regarding the performance of nnU-Net against imaging artifacts, it is important to note that while nnU-Net’s default data augmentation (e.g., Gaussian noise, blurring, and simulated intensity shifts) provides a baseline level of robustness, it is not inherently designed to recognize domain-specific artifacts like water droplets or agar scars as "background."

Our strategy addressed this through an iterative approach. Each time we encounter "failure case" images where scars in the agar, droplets, condensation or possible light reflections initially caused segmentation errors, we manually correct them, and re-integrate them into the training dataset. Empirically, we found that incorporating these specific artifacts directly into the training set led to a significant increase in

performance. Because our hardware maintains a fixed light source and camera geometry, these artifacts (droplets and agar disturbances) are the primary sources of noise; by training nnU-Net to explicitly classify them as background, the model achieves high stability across developmental timeframes.

We plan to continue this strategy in the future, evolving our training set and model weights.

Comment 7:
2 - What is the minimal detection length for lateral roots (as I guess it depends on the skeletonization process and related noise on main root)?

Response 7:
In our pipeline, the spatial resolution is approximately 0.04~0.05 mm/pixel. To mitigate skeletonization noise and ensure that spurs on the root axes are not incorrectly identified, we implement a structural pruning threshold on the skeleton prior to graph construction. This threshold is set to a minimum of 5 pixels, which establishes a theoretical minimal detection length of 0.2 mm.
Furthermore, as now described in the manuscript in lines 340-355, we also apply a temporal verification window during measurement post-processing. Any measurement, including root detections, is only validated if the structure persists for at least 6 hours, effectively filtering out transient artifacts or temporary occlusions. These combined spatial and temporal filters ensure biological consistency for all tracked organs.

Comment 8:
3 - The measurements results that can be exported in RSML format is a nice feature for interoperability, great job!

Response 8:

Thank you!

Comment 9:
4 - (page 6; section "Software Implementation and User Interface") "The interface includes quality control through visual feedback systems": I don't understand the meaning of this sentence.
Response 9:
We have revised this section of the manuscript to clarify that "visual feedback" refers to the interactive validation steps built into the software workflow. Users can first inspect the segmentation quality on the raw video before selecting individual plants and defining their specific root starting positions. After the automated graph construction, the interface allows the user to watch the growth video with the resulting skeletonized graph and measurements overlaid in real time. This allows for a direct visual inspection of the tracking performance, ensuring that any plant with biological or technical anomalies can be discarded or re-analyzed before being included in the final automated reports and statistical investigations.
This was incorporated in the tutorials and in lines 445-453 in the manuscript

Comment 10:
5 - (page 5; section "Multiple Plant Screening Analysis") "The hypocotyl analysis module incorporates validation steps for reliable measurement in multi-plant scenarios. The system automatically detects physiologically impossible growth rates and artifacts that can occur when different plants touch and their segmentations combine. Biological constraints are enforced through validation of non-decreasing length measurements and growth directions against expected gravitropic responses."
This feature is an interesting QC check, but I was wondering if user can disable it. I couldn't test it, so the following is based on conjectures: what is the threshold(s) for the impossible growth rates? Can user edit this threshold(s)? For the gravitropic responses, does the analysis assume gravity is directed to the bottom of images? I'm thinking about a very particular experimental case, but given the nature of the ChronoRoot hardware, I think this would be the typical setup for nomad experiments, especially for experiments in low gravity (in the ISS for example). In this particular case, I guess that the gravitropic responses could be studied and users would like to define the gravity field (or no gravity field). For this reason, I think it would be nice to enable users to decide if they want the QC check or not. Again, this is a very particular case, but it illustrates how a validation steps can limit the range of applications of a given setup.

Response 10:

We thank the reviewer for this observation. We agree that hard-coded assumptions about growth direction could limit the tool's applicability in novel setups, such as the microgravity or inverted experiments mentioned. In response, we have updated the analysis module to remove the directional gravitropic constraint, without finding any problems in the reproduction of our results.

Still, this validation step serves as a high-constraint filter primarily designed to detect and handle plant overlaps. When seedlings touch, segmentation algorithms often merge them, resulting in sudden, non-biological jumps in measured size. To prevent these artifacts from corrupting the dataset, the system automatically identifies and removes these compromised traces from the processed results. To address the reviewer's suggestion regarding flexibility without adding complex UI toggles, we explicitly rely on data transparency. Since the processed output prioritizes statistical purity, we ensure that researchers always have access to the raw, unfiltered measurements. This allows users to bypass our collision filtering entirely and apply custom validation logic if their specific experiment requires analyzing touching plants or unusual growth patterns. This was incorporated to the manuscript in lines 376-381.

Comment 11:
6 - I think some figures would look better in log scale: Figures 3B-3C, spectra in Figure 4, germination curves in Figure 6

Response 11:

Figures 3B-3C were changed to tables 2, 3 and 4 according to reviewer’s comments to help the readability. As for the germination curves, we removed the later time point where no change was visible, increasing the graph time-resolution where the majority of the changes are happening. We believe this makes the current version without log transformation already more readable.

Comment 12:
7 - In Figure 4's legend, the number of replicates per conditions is missing; it is an important metric as mean curves and standard deviations are used.

Response 12:

Figures 3, 4 and 5 captions now include the number of replicates per condition.

Comment 13:
8 - Figure 4D: why some negative values for LR length?

Response 13:

Figure 4D (now 3D in the revised manuscript) is intended to show how the curves vary when we modify the different values of the Principal Components, for interpretability purposes. As we vary these components to extreme values, non plausible solutions emerge. As you can see, the negative values in the explanations correspond to curves with very high (PC2) or very low (PC1) values.

Comment 14:
9 - For the FFT analysis and circadian cycles, it would be great to conduct the analysis on plants keeping in dark: how does it translate to the power spectrum?

Response 14:

This is a very interesting suggestion. However, while the question is relevant, our paper focuses on presenting a case study analysis of ChronoRoot’s enhancements, rather than conducting new experiments

to address additional biological questions. That is the reason why we believe that such an analysis is out of the scope of our current work, and would be more appropriately explored in a dedicated paper examining the regulation of root architecture by light regimes.

Comment 15:
10 - Some statistical significance tests are missing:
* difference between spectra and significance of peaks (Figure 4C)
* Figures 5B & S4

Response 15:

This was incorporated in the figures.

Comment 16:
11 - I would like to see the graphs PC1 vs. PC2 for the fPCA analyses; it's always informative to have a visual overview of the PCA projections (maybe as supplementary figures if it doesn't fit the main ones?).

Response 16:

We thank the reviewer for the idea. It is now included in Use Cases 1 (Figure 2), 3 (Figure S4) and 4 (Figure 6) .

Comment 17:
12 - It might be over my head, but I have the feeling that the fPCA is missing important correlations between morphological metrics (especially MR vs. LR lengths). It is likely due to the fact that the fPCA is conducted on a projection of the multi-dimensional dataset [MR(t), LR(t), TR(t), etc.]_i to one of its dimensions (for example MR(t)); hence losing the temporal correlations between this measurement and others. I was wondering to what extent it would be feasible to integrate all morphological metrics for a given plant to a single fPCA? I would be interested in authors' thoughts on this.

Response 17:
We thank the reviewer for this insightful observation. Indeed, the analysis currently presented in the manuscript performs functional PCA independently for each morphological metric (e.g., MR, LR, TR), and therefore does not explicitly model cross-metric temporal correlations.
From a methodological standpoint, the reviewer is correct that a multivariate extension of fPCA (often referred to as multivariate or vector-valued fPCA) could, in principle, integrate several time-varying morphological descriptors into a single joint decomposition. However, this approach would add significant methodological complexity, require careful balancing between traits, and lead to components that are much harder to interpret biologically than the univariate fPCA used here.
Our primary objective in this work is to provide a robust, interpretable, and broadly accessible framework for phenotypic time-series analysis within ChronoRoot. The current per-trait fPCA design was chosen deliberately to maximize interpretability and usability for non-expert users, while still capturing the dominant temporal modes of variation for each biologically meaningful metric. Implementing a full multivariate fPCA pipeline would significantly increase the methodological burden of the tool and shift it away from this design philosophy.
We therefore consider multivariate fPCA to be a valuable direction for advanced users who wish to perform more specialized integrative analyses, and we provide access to all raw temporal measurements so that such analyses can be carried out externally using dedicated statistical frameworks.

Comment 18:
13 - Some typos:
* Use case 1 paragraph refers to Figure 5, while it's actually Figure 4
* User case 3 paragraph mentions Figure 5, while it should be Figure 6
* In figure 4, color legends have been switched
* In use case 2 "construct bearing 2000-pb region": should be "2000 bp"

Response 18:

We have fixed all these typos.
Reviewer 3
Comment 1:
This paper presents a new machine learning and software pipeline for temporal analysis of arabidopsis seedlings. The work improves upon prior work by the same authors on Chronoroot, and shows good performance. Notably, the machine learning pipelines, measurement / analysis and user experience are substantially improved.

I have no major concerns over this paper, I think it is very good. The use cases did a good job of showing the benefits of the software, and the figures are of very high quality. The work outputs standardized file formats and will be a welcome addition to the community work in this area. The github repository hosting the software is well put together, with excellent instructions and guidance on installation and use. I have some minor suggestions for the manuscript:

- It isn't clear what resolution of images are being used during the image processing and machine learning pipeline. The native resolution of the images is quoted. It may be that nnUnet handles this, but it is useful information to include. In general the detail on the training of the networks is fairly vague, again perhaps nnUnet handles this but these parameters remain useful should someone wish to improve upon the network. Is tiling used, resizing? What about training vs inference?

Response 1:

We thank the reviewer for this observation. Images are captured at native 3280 x 2464 resolution (approximately 0.04 mm per pixel) and processed by nnUNet at full single-channel resolution without resizing. This is now detailed in Lines 184-189.

The nnUNet framework self-configures its architecture based on dataset properties, automatically determining optimal patch sizes, that is the full image in our case, network depth, kernel sizes, stride patterns, normalization schemes, and learning rate schedules during the planning phase prior to training. This self-configuration eliminates the need for manual hyperparameter tuning.

Comment 2:
- Related to this, there is a throwaway line in the discussion that it is easy to retrain to new species because of the use of nnUnet. More info here is required to make this claim, presumably you are not specifically facilitating this via the software? Training nnunet doesn't necessarily mean that the output will be compatible with your measurement pipeline, depending on the size and shape of the species? Your manuscript submission includes a fork of the nnUnet repo - what changed in your version, should people training use the original or your copy? Perhaps a more straightforward question would be, would re-training of the network require someone with a strong technical background?

Response 2:

We apologize for the confusion regarding the nnUNet repository reference. During initial development of ChronoRoot 2.0, we worked with nnUNet version 1.x, which required minor modifications for integration. ChronoRoot 2.0 now uses the official nnUNet v2 repository (https://github.com/MIC-DKFZ/nnUNet) without any modifications to the framework itself. The improved modularity of nnUNet v2 eliminated the need for custom changes.

Regarding retraining for new species: the process requires modest technical skills but no machine learning expertise. The workflow consists of: (1) manually annotating a set of images (we recommend 300-500 images minimum) using ITK-SNAP with the same five or six-class scheme (main root, lateral roots, seed, hypocotyl, aerial (or leaves and petiole), (2) organizing images and annotations following nnUNet's

standardized folder structure, (3) executing standard nnUNet preprocessing and training commands, and (4) copying the trained weights to the ChronoRoot model directory. nnUNet automatically handles architecture configuration, hyperparameter optimization, and cross-validation, where no manual tuning is required. A researcher comfortable with command-line operations and basic file management can complete this workflow by following our documentation. As for step 2, probably the most arbitrary one, we provided scripts to convert from our ground truth data to the nnUNet format. We are working on improving our documentation for this particular case, which can be found in our dedicated website at https://chronoroot.github.io/tutorials/training_on_your_images/.

To better illustrate how our model can be adapted to new species, we are now including experiments in Tomato species (see lines 162-182, 568-594), where the model has been retrained and repurposed for this plant.

Comment 3:
- It might be useful to extend your consideration of the limitations of the work - for example the age, species etc. of the seedlings you expect to work here. Other software in this space often struggles with well-established root systems with many crossovers, you mention this briefly.

Response 3:

We thank the reviewer for this suggestion. Key constraints include:

Temporal limitations: ChronoRoot hardware allows the imaging in classical 12cmx12cm square plates, and a lot of limitations come from this restricted space. Experiments typically conclude when the main root reaches the plate bottom (usually 10-14 days for Arabidopsis, 5-7 days for tomato), as roots may then grow along the bottom surface (invisible to imaging) or cease elongation and increase lateral root production. To ensure comparable developmental stages, researchers select analysis timeframes after reviewing complete image sequences.

Plant density and crowding:
The Standard Interface supports 4-6 plants per plate for extended tracking, as higher densities lead to root system overlap that prevents individual architectural analysis.
The Screening Interface accommodates up to 100 plants per plate but limits analysis to early development (3-5 days) before crowding occurs. When plants physically touch, segmentation cannot reliably separate individuals.

Species and developmental stage: The system is optimized for seedling-stage analysis when organs are clearly distinguishable. Mature plants with dense root systems featuring numerous crossovers and overlapping lateral roots present challenges for accurate segmentation and tracking. Other species with very different morphologies from our training data (Arabidopsis and tomato) will require retraining with appropriately annotated data.

We included this in Discussions line 798-809

Reviewer 4

Comment 1:
Reproducibility report for: ChronoRoot 2.0: An Open AI-Powered Platform for 2D Temporal Plant Phenotyping

- Recommendations for authors
We recommend the following improvements based on our observations to enhance the study's reproducibility:

-- ROI selection and seed placement: The results depend heavily on ROI selection and seed placement. However, the tutorial did not mention how to choose this to reproduce the end result. Adding this would improve reproducibility.

Response 1:
The reviewer is correct that the manual initialization step is the source of the numerical variation.
In the Standard Interface, the "Seed Placement" does not merely indicate the location of the seed; it defines the spatiotemporal origin of the main root's growth. To ensure accurate quantification, the user must advance the video timeline to the specific frame where the main root begins to emerge and segment, and select the point at that moment.
If the point is selected at the beginning of the video (Day 0) before emergence, the algorithm may integrate slight segmentation noise or seed coat artifacts during the dormancy period, leading to the differences in length and p-values observed by the reviewer. When the point is selected at the precise moment of emergence, the noise above this point is effectively filtered.
Tutorials for the interface now include many examples and explanations on how to place the initial point, for both tomato and arabidopsis.
Available at our new website: https://chronoroot.github.io/
Comment 2:
-- MacOS installation guide: An installation guide is provided for Linux and Windows. Adding a guide for MacOS users would enhance reproducibility.

Response 2:

We included a small section on our new docker tutorial at https://chronoroot.github.io/tutorials/docker/. We acknowledge that our support is mostly Linux and Windows 10 at the moment, and we will continue working on better support.

Comment 3:
-- p-value reporting: The p-value reported in Figs. 4B and 4D is currently only through an asterisk, indicating significance. Reporting exact values improves reproducibility.

Response 3:

We appreciate the reviewer's focus on reproducibility. To clarify, the figures presented in the use cases are direct exports from the ChronoRoot 2.0 interface, designed to showcase the platform's automated visualization and reporting capabilities. We have now standardized the notation across all figures, using * for $p<0.05$ and ** for $p<0.001$.
Regarding the reporting of exact values: because these use cases are functional demonstrations of the software's output, we chose to maintain the visual format a user would see in the software's dashboard. However, we have added a clarification to the manuscript stating that for every analysis, ChronoRoot 2.0 automatically generates text files containing the exact p-values, mean, standard deviation, and sample sizes (n) for every parameter. This ensures that while the visual output is optimized for quick interpretation, the raw numerical data is always preserved for rigorous verification. We have updated the text in lines 607–614 to make this explicit.

Cookie Preference Center

We use cookies which are necessary to make our site work. We may also use additional cookies to analyze, improve and personalize our content and your digital experience. For more information, see our Cookie Policy.

You may choose not to allow some types of cookies. However, blocking some types may impact your experience of our site and the services we are able to offer. See the different category headings below to find out more or change your settings.

Allow all

Manage Consent Preferences

Strictly Necessary Cookies

Always active

These cookies are necessary for the website to function and cannot be switched off in our systems. They are usually only set in response to actions made by you which amount to a request for services, such as setting your privacy preferences, logging in or filling in forms. You can set your browser to block or alert you about these cookies, but some parts of the site will not then work.

Functional Cookies

These cookies enable the website to provide enhanced functionality and personalisation. They may be set by us or by third party providers whose services we have added to our pages. If you do not allow these cookies then some or all of these services may not function properly.

Performance Cookies

These cookies allow us to count visits and traffic sources so we can measure and improve the performance of our site. They help us to know which pages are the most and least popular and see how visitors move around the site.

Targeting Cookies

These cookies may be set through our site by our advertising partners. They may be used by those companies to build a profile of your interests and show you relevant adverts on other sites. If you do not allow these cookies, you will experience less targeted advertising.

Cookie List

Clear

Apply Cancel
Consent Leg.Interest

Confirm my choices

Powered by onetrus
