# Supplementary material for: ChronoRoot 2.0: an open AI-powered platform for 2D temporal plant phenotyping
Source: Gigascience. 2026 Feb 28;15:giag018. doi: 10.1093/gigascience/giag018 (PMC13042295; doi:10.1093/gigascience/giag018)
Supplement: giag018_Reviewer_1_Report_original_submission — Reviewer 1 -- 8/6/2025 [file giag018_reviewer_1_report_original_submission.pdf]

Reviewer Report

Title: ChronoRoot 2.0: An Open AI-Powered Platform for 2D Temporal Plant Phenotyping

Version: Original Submission **Date:** 8/6/2025

Reviewer name: Reviewer 1

Reviewer Comments to Author:

This manuscript presents ChronoRoot 2.0, a platform that significantly enhances the continuous phenotyping of plant root systems and other organs by integrating a multi-class segmentation model based on nnUNet with low-cost hardware. The article is well-structured, methodologically sound, and presents good results, demonstrating strong engineering integration and technical depth. The following suggestions are provided from a practical perspective:1. Generalization across species remains unverifiedAlthough the authors claim that the model is easily transferable to other plant species, all current experiments are limited to Arabidopsis thaliana. It is recommended to add other results on at least one non-Arabidopsis species to strengthen the claim of cross-species generalizability.2. Segmentation performance is not clearly presentedThe multi-class segmentation results are mainly shown through figures. It would be helpful for reading and understanding to show these results with quantitative metrics such as Dice coefficient and Hausdorff distance for each structure in tabular format.3. Limited system generalizabilityThe proposed platform strictly relies on a specific hardware-software setup (e.g., infrared image input only), which restricts its applicability to other root imaging datasets. In comparison, tools such as RPT: An integrated root phenotyping toolbox have demonstrated the ability to handle more complex root architectures. Even the authors mention that using model fine-tuning is possible to improve its generalization, however nnUNet model is quite sensitivity to the input data distribution, therefore further evaluation on more diverse and complex datasets is recommended.4. Software setup remains challengingAlthough the authors provide a well-structured code and detailed documentation of the software, the installation process involves numerous dependencies and remains difficult for users without a computational background. It is suggested to release an all-in-one installation package (e.g., a standalone executable) to lower the barrier for broader adoption.

Level of Interest

Please indicate how interesting you found the manuscript: Choose an item.

Quality of Written English

Please indicate the quality of language in the manuscript: Choose an item.

Declaration of Competing Interests

Please complete a declaration of competing interests, considering the following questions:

- Have you in the past five years received reimbursements, fees, funding, or salary from an organisation that may in any way gain or lose financially from the publication of this manuscript, either now or in the future?
- Do you hold any stocks or shares in an organisation that may in any way gain or lose financially from the publication of this manuscript, either now or in the future?
- Do you hold or are you currently applying for any patents relating to the content of the manuscript?
- Have you received reimbursements, fees, funding, or salary from an organization that holds or has applied for patents relating to the content of the manuscript?
- Do you have any other financial competing interests?
- Do you have any non-financial competing interests in relation to this paper?

If you can answer no to all of the above, write 'I declare that I have no competing interests' below. If your reply is yes to any, please give details below.

I declare that I have no competing interests.

I agree to the open peer review policy of the journal. I understand that my name will be included on my report to the authors and, if the manuscript is accepted for publication, my named report including any attachments I upload will be posted on the website along with the authors' responses. I agree for my report to be made available under an Open Access Creative Commons CC-BY license (http://creativecommons.org/licenses/by/4.0/). I understand that any comments which I do not wish to be included in my named report can be included as confidential comments to the editors, which will not be published.

Choose an item.

To further support our reviewers, we have joined with Publons, where you can gain additional credit to further highlight your hard work (see: https://publons.com/journal/530/gigascience). On publication of this paper, your review will be automatically added to Publons, you can then choose whether or not to claim your Publons credit. I understand this statement.

Yes Choose an item.
