# Supplementary material for: ChronoRoot 2.0: an open AI-powered platform for 2D temporal plant phenotyping
Source: Gigascience. 2026 Feb 28;15:giag018. doi: 10.1093/gigascience/giag018 (PMC13042295; doi:10.1093/gigascience/giag018)
Supplement: giag018_Reviewer_2_Report_original_submission — Reviewer 2 -- 8/8/2025 [file giag018_reviewer_2_report_original_submission.pdf]

Reviewer Report

Title: ChronoRoot 2.0: An Open AI-Powered Platform for 2D Temporal Plant Phenotyping

Version: Original Submission **Date:** 8/8/2025

Reviewer name: Reviewer 2

Reviewer Comments to Author:

In their manuscript entitled "ChronoRoot 2.0: An Open AI-Powered Platform for 2D Temporal Plant Phenotyping", the authors present an updated version of their previously published framework called ChronoRoot, a tool aimed at the segmentation and quantification of morphological features of plants, in particular their root system. New features have been added, the major one being a graphical interface developed to lower the computational barrier that command lines interfaces constitute for researchers without a bioinformatics background. The segmentation performances have been improved using a deep learning method based on the nnUNet architecture, which, according to authors, makes it easier to adapt to other plant species than A. thaliana. The framework can measure a large variety of morphological parameters of plants such as the length of main/lateral roots, the convex hull area, the emergence angle of roots and can also segment and classify up to 6 organs of plants (including leaves). Temporal analysis through Fourier analysis is available, as well as functional principal component analysis (fPCA), that is used by authors to discover hidden features of plants growth. Through three use cases, authors highlight the different functionalities of ChronoRoot 2.0, showing the improvements compared to the first version of the framework.Overall, the manuscript is well written and the logic underlying the study is quite easy to follow. The metrics used and analytical methods are relevant given the objectives of ChronoRoot. However, the installation of the software (through Docker), despite being extensively described by authors in the related GitHub, is far from being easy, and I could not run it correctly even after a few hours of investigations. As a Windows user, I had to adapt the commands and find "tricks" to finally install the software, I guess that a Linux user would have less issues for the installation, but as the tool, with its GUI, is targeted to biologists without a computational background, the use of Docker is already a barrier; so with additional issues coming from a non-UNIX OS, I'm afraid that the target is slightly missed: users able to install ChronoRoot 2.0 are likely the ones with computational background, and then do not need a GUI.While authors provided an extensive documentation for the installation, there are no guidelines or tutorial for the use of ChronoRoot itself; I am then lost when trying to use any of the three components (standard, screening or segmentation tools); there are no indications of the format required for the videos, the filenames, folders architecture, parameters, etc. A step-by-step tutorial with toy data for each components (standard, screening, segmentation) would be more than needed in order to help users learn how to use the framework (or simply check that the installation is correct; currently, I couldn't say if it's not running on my computer because of an incorrect installation or me not knowing how to correctly use the software…)While I recognize the value of Docker for long-term maintenance and version compatibility, I think this still requires basic computational skills that will discourage most of the targeted users. A standalone executable version (ideally three versions for Windows, Linux or Mac) would be really great to have in

addition to the Docker solution, so it would make it easier for beginners to adopt ChronoRoot as part of their workflow.In summary, I'm convinced that ChronoRoot 2.0 with its new GUI and improved morphometrics tools, would be a great framework for the plant biologists community, and while I haven't been able to run the software myself, I am confident that it actually performs the features described in the associated manuscript. But I think authors should work to improve the accessibility of their tool before the manuscript can be accepted.The following is a detailed list of my comments, from the major to minor comments:[MAJOR]1 - Clear tutorial missing on how to use the software (standard, screening and segmentation components). It would be great to have a doc for each component associated with example data, which users can download in order to reproduce the results shown in the tutorial.2 - A standalone executable version of ChronoRoot 2.0 would be great to have (especially for non-UNIX users).3 - ChronoRoot 2.0, with the nnUNet architecture is said to be easily adaptable to diverse plant species analysis; but authors never showed this claim on other species than A. thaliana. It would be great to test the segmentation and morphometrics measurements on other publicly available datasets with other species than A. thaliana; this would prove the claim and also highlight the interoperability of ChronoRoot (which is not restricted to ChronoRoot hardware setup).4 - Similarly, authors compared the segmentation performance of ChronoRoot 2.0 versus its first version; but as other segmentation tools exist, it would be great to compare ChronoRoot 2.0 to the most recent ones, as it's a critical parameter when choosing between available tools.5 - I don't understand why users need to manually define the ROI when using the standard interface, while it is automatically detected with the screening interface; similarly, the segmentation methods are different between these two interfaces, the standard one being more accurate than the screening one (as far as I understand the manuscript, since I couldn't test it myself). It would be great to have the choice both for the ROI settings (it could be an automatic detection, and users could then manually edit the ROI if needed) and for the segmentation method (users could prefer using the screening interface with accurate segmentation, even if it's more computationally expensive).[MINOR]1 - (page 4; section "Specialized Processing Pipelines") "This approach effectively addresses common imaging challenges in plant phenotyping, such as water droplets, condensation artifacts, or temporary occlusions, providing stable root structure detection throughout developmental timeframes.": a reference is missing.In a similar frame, how does nnUNet perform against imaging artefacts?2 - What is the minimal detection length for lateral roots (as I guess it depends on the skeletonization process and related noise on main root)?3 - The measurements results that can be exported in RSML format is a nice feature for interoperability, great job!4 - (page 6; section "Software Implementation and User Interface") "The interface includes quality control through visual feedback systems": I don't understand the meaning of this sentence.5 - (page 5; section "Multiple Plant Screening Analysis") "The hypocotyl analysis module incorporates validation steps for reliable measurement in multi-plant scenarios. The system automatically detects physiologically impossible growth rates and artifacts that can occur when different plants touch and their segmentations combine. Biological constraints are enforced through validation of non-decreasing length measurements and growth directions against expected gravitropic responses."This feature is an interesting QC check, but I was wondering if user can disable it. I couldn't test it, so the following is based on conjectures: what is the threshold(s) for the impossible growth rates? Can user edit this threshold(s)? For the gravitropic responses, does the analysis assume gravity is directed to the bottom of images? I'm thinking about a very particular experimental case, but given the nature of the ChronoRoot hardware, I think this would

be the typical setup for nomad experiments, especially for experiments in low gravity (in the ISS for example). In this particular case, I guess that the gravitropic responses could be studied and users would like to define the gravity field (or no gravity field). For this reason, I think it would be nice to enable users to decide if they want the QC check or not. Again, this is a very particular case, but it illustrates how a validation steps can limit the range of applications of a given setup.6 - I think some figures would look better in log scale: Figures 3B-3C, spectra in Figure 4, germination curves in Figure 67 - In Figure 4's legend, the number of replicates per conditions is missing; it is an important metric as mean curves and standard deviations are used.8 - Figure 4D: why some negative values for LR length?9 - For the FFT analysis and circadian cycles, it would be great to conduct the analysis on plants keeping in dark: how does it translate to the power spectrum?10 - Some statistical significance tests are missing:* difference between spectra and significance of peaks (Figure 4C)* Figures 5B & S411 - I would like to see the graphs PC1 vs. PC2 for the fPCA analyses; it's always informative to have a visual overview of the PCA projections (maybe as supplementary figures if it doesn't fit the main ones?).12 - It might be over my head, but I have the feeling that the fPCA is missing important correlations between morphological metrics (especially MR vs. LR lengths). It is likely due to the fact that the fPCA is conducted on a projection of the multi-dimensional dataset [MR(t), LR(t), TR(t), etc.]_i to one of its dimensions (for example MR(t)); hence losing the temporal correlations between this measurement and others. I was wondering to what extent it would be feasible to integrate all morphological metrics for a given plant to a single fPCA? I would be interested in authors' thoughts on this.13 - Some typos:* Use case 1 paragraph refers to Figure 5, while it's actually Figure 4* User case 3 paragraph mentions Figure 5, while it should be Figure 6* In figure 4, color legends have been switched* In use case 2 "construct bearing 2000-pb region": should be "2000 bp"

Level of Interest

Please indicate how interesting you found the manuscript: Choose an item.

Quality of Written English

Please indicate the quality of language in the manuscript: Choose an item.

Declaration of Competing Interests

Please complete a declaration of competing interests, considering the following questions:

- Have you in the past five years received reimbursements, fees, funding, or salary from an organisation that may in any way gain or lose financially from the publication of this manuscript, either now or in the future?
- Do you hold any stocks or shares in an organisation that may in any way gain or lose financially from the publication of this manuscript, either now or in the future?
- Do you hold or are you currently applying for any patents relating to the content of the manuscript?

- Have you received reimbursements, fees, funding, or salary from an organization that holds or has applied for patents relating to the content of the manuscript?
- Do you have any other financial competing interests?
- Do you have any non-financial competing interests in relation to this paper?

If you can answer no to all of the above, write 'I declare that I have no competing interests' below. If your reply is yes to any, please give details below.

I declare that I have no competing interests

I agree to the open peer review policy of the journal. I understand that my name will be included on my report to the authors and, if the manuscript is accepted for publication, my named report including any attachments I upload will be posted on the website along with the authors' responses. I agree for my report to be made available under an Open Access Creative Commons CC-BY license (http://creativecommons.org/licenses/by/4.0/). I understand that any comments which I do not wish to be included in my named report can be included as confidential comments to the editors, which will not be published.

Choose an item.

To further support our reviewers, we have joined with Publons, where you can gain additional credit to further highlight your hard work (see: https://publons.com/journal/530/gigascience). On publication of this paper, your review will be automatically added to Publons, you can then choose whether or not to claim your Publons credit. I understand this statement.

Yes Choose an item.
