# Supplementary material for: ChronoRoot 2.0: an open AI-powered platform for 2D temporal plant phenotyping
Source: Gigascience. 2026 Feb 28;15:giag018. doi: 10.1093/gigascience/giag018 (PMC13042295; doi:10.1093/gigascience/giag018)
Supplement: giag018_Reviewer_3_Report_original_submission — Reviewer 3 -- 8/22/2025 [file giag018_reviewer_3_report_original_submission.pdf]

Reviewer Report

Title: ChronoRoot 2.0: An Open AI-Powered Platform for 2D Temporal Plant Phenotyping

Version: Original Submission **Date:** 8/22/2025

Reviewer name: Reviewer 3

Reviewer Comments to Author:

This paper presents a new machine learning and software pipeline for temporal analysis of arabidopsis seedlings. The work improves upon prior work by the same authors on Chronoroot, and shows good performance. Notably, the machine learning pipelines, measurement / analysis and user experience are substantially improved.I have no major concerns over this paper, I think it is very good. The use cases did a good job of showing the benefits of the software, and the figures are of very high quality. The work outputs standardized file formats and will be a welcome addition to the community work in this area. The github repository hosting the software is well put together, with excellent instructions and guidance on installation and use. I have some minor suggestions for the manuscript:- It isn't clear what resolution of images are being used during the image processing and machine learning pipeline. The native resolution of the images is quoted. It may be that nnUnet handles this, but it is useful information to include. In general the detail on the training of the networks is fairly vague, again perhaps nnUnet handles this but these parameters remain useful should someone wish to improve upon the network. Is tiling used, resizing? What about training vs inference?- Related to this, there is a throwaway line in the discussion that it is easy to retrain to new species because of the use of nnUnet. More info here is required to make this claim, presumably you are not specifically facilitating this via the software? Training nnunet doesn't necessarily mean that the output will be compatible with your measurement pipeline, depending on the size and shape of the species? Your manuscript submission includes a fork of the nnUnet repo - what changed in your version, should people training use the original or your copy? Perhaps a more straightforward question would be, would re-training of the network require someone with a strong technical background?- It might be useful to extend your consideration of the limitations of the work - for example the age, species etc. of the seedlings you expect to work here. Other software in this space often struggles with well-established root systems with many crossovers, you mention this briefly.

Level of Interest

Please indicate how interesting you found the manuscript: Choose an item.

Quality of Written English

Please indicate the quality of language in the manuscript: Choose an item.

Declaration of Competing Interests

Please complete a declaration of competing interests, considering the following questions:

- Have you in the past five years received reimbursements, fees, funding, or salary from an organisation that may in any way gain or lose financially from the publication of this manuscript, either now or in the future?
- Do you hold any stocks or shares in an organisation that may in any way gain or lose financially from the publication of this manuscript, either now or in the future?
- Do you hold or are you currently applying for any patents relating to the content of the manuscript?
- Have you received reimbursements, fees, funding, or salary from an organization that holds or has applied for patents relating to the content of the manuscript?
- Do you have any other financial competing interests?
- Do you have any non-financial competing interests in relation to this paper?

If you can answer no to all of the above, write 'I declare that I have no competing interests' below. If your reply is yes to any, please give details below.

I declare that I have no competing interests

I agree to the open peer review policy of the journal. I understand that my name will be included on my report to the authors and, if the manuscript is accepted for publication, my named report including any attachments I upload will be posted on the website along with the authors' responses. I agree for my report to be made available under an Open Access Creative Commons CC-BY license (http://creativecommons.org/licenses/by/4.0/). I understand that any comments which I do not wish to be included in my named report can be included as confidential comments to the editors, which will not be published.

Choose an item.

To further support our reviewers, we have joined with Publons, where you can gain additional credit to further highlight your hard work (see: https://publons.com/journal/530/gigascience). On publication of this paper, your review will be automatically added to Publons, you can then choose whether or not to claim your Publons credit. I understand this statement.

Yes Choose an item.
