# Supplementary material for: ChronoRoot 2.0: an open AI-powered platform for 2D temporal plant phenotyping
Source: Gigascience. 2026 Feb 28;15:giag018. doi: 10.1093/gigascience/giag018 (PMC13042295; doi:10.1093/gigascience/giag018)
Supplement: giag018_Reviewer_4_Report_original_submission — Reviewer 4 -- 9/17/2025 [file giag018_reviewer_4_report_original_submission.pdf]

Reviewer Report

Title: ChronoRoot 2.0: An Open AI-Powered Platform for 2D Temporal Plant Phenotyping

Version: Original Submission **Date:** 9/17/2025

Reviewer name: Reviewer 4

Reviewer Comments to Author:

Reproducibility report for: ChronoRoot 2.0: An Open AI-Powered Platform for 2D Temporal Plant PhenotypingJournal: GigaScienceID number/DOI: GIGA-D-25-00276Reviewer(s): Laura Caquelin, Department of Clinical Neuroscience, Karolinska Institutet, Sweden [Wrote the summary of the study and the scope of reproducibility section and reviewed the final report.]Tobias Wầngberg, Department of Clinical Neuroscience, Karolinska Institutet, Sweden [Worked on reproducing the results and contributed to writing the report.]--1. Summary of the StudyThe authors developped ChronoRoot 2.0, an open-source platform for temporal plant phenotyping, combining nnUNet architecture for multi-class segmentation. It was validated on Arabidopsis thaliana through three use cases.--2. Scope of reproducibilityFor the purpose of this review, we focus on the analysis comparing root growth dynamics under different light conditions.- Outcome: Main and lateral root length over time, analyzed using Functional Principal Component Analysis (FPCA).- Analysis method outcome: FPCA was used to compare growth trajectories (Figure 5-B and 5-D). Boxplots show distributions of PC scores. Asterisks indicate statistical differences between light conditions, though the statistical test used is not specified.- Main result: "The first functional principal component (PC1) captured the primary growth trajectory of roots (Figure 5-B), revealing differences between photoperiod conditions. The second (PC2) showed distinct divergence between long-day and continuous-light conditions, indicating temporal shifts in growth patterns. […] Similar analysis of lateral root length (Figure 5-D) showed comparable patterns." Page 8, section "Use Case 1".--3. Availability of Materialsa. Data- Data availability:-- Experimental time-lapse sequences: not open.-- Annotated training/validation set: open.-- Demo datasets: open.- Data completeness:-- Experimental time-lapse sequences: --- Annotated training/validation set: complete-- Demo datasets: complete- Access Method:-- Experimental time-lapse sequences: --- Annotated training/validation set: Huggingface-- Demo datasets: Docker image- Repository:-- Experimental time-lapse sequences: --- Annotated training/validation set: https://huggingface.co/datasets/ngaggion/ChronoRoot_nnUNet.-- Demo datasets: https://hub.docker.com/r/ngaggion/chronoroot- Data quality:The data is structured and clear for the training data set and the demo datasets. The experimental time-lapse data used for evaluating the software was not shared due to computational limitations (see Section 5.2 below for details).b. Code- Code availability: Open- Programming Language(s): Python- Repository link: https://github.com/ngaggion/ChronoRoot2/tree/master- License: GNU General Public License v3.0.- Repository status: Public- Documentation: The repository comes with clear documentation. A potential improvement is to provide an installation guide for MacOS users.---4. Computational environment of reproduction analysis- Operating system

for reproduction: MacOS 15.6.1 (Ubuntu 22.04.4 LTS inside the Docker image).- Programming Language(s): Python.- Code implementation approach: Using shared code.- Version environment for reproduction: Python 3.8.2 (inside docker image).--5. Results5.1 Original study results- Results: The two leading PCs of the FPCA as shown in Figs. 4B and 4D in the mansucript as well as Mann-Whitney tests comparing long-day and continuous-light conditions for PC2 of the growth of the main root length and lateral root length.5.2 Steps for reproduction-> Reproduce manuscript FPCA- Issue 1: Unable to find data to reproduce the results.-- Resolved: The authors had not shared the data since it exceeded 150 GB in size. Moreover, reproducing the results on this data would take over a week of computing time making it unfeasible for the scope of this review. To address the issue the authors shared demo datasets along with tutorials for how to reproduce the results. The correspondence is shown below.[Email sent to the authors #1]I am currently conducting a reproducibility review of your manuscript titled "ChronoRoot 2.0: An Open AI-Powered Platform for 2D Temporal Plant Phenotyping" (GIGA-D-25-00276).I am trying to reproduce the analysis for Use case 1 but have been unable to find the required data on the growth of the Arabidopsis seeds from the files you shared. Could you please direct me to the proper location for the experimental data?[Response received from the authors #1]Thank you for your patience with the reproducibility review. I apologize for the confusion regarding data access for Use Case 1.The current Use Cases 1, 2, and 3 represent comprehensive demonstrations of ChronoRoot 2.0's capabilities. The root analysis of Use Cases 1 and 2 involved 40+ plants each that require individual plant-by-plant root analysis. The raw images require more than 150GB of data each and substantial computational resources (up to a week of GPU processing time plus additional analysis days). These use cases were designed to showcase the full analytical power of our platform using complete experimental datasets from already published papers: Use Case 1 data come from the original ChronoRoot publication (https://academic.oup.com/gigascience/article/10.7/giab052/6324285), and Use Case 2 data come from "The transcription factor NF-YA10 determines the area explored by Arabidopsis thaliana roots and directly regulates LAZY genes" (https://onlinelibrary.wiley.com/doi/full/10.1111/tpj.70016). Use Case 3 demonstrates high-throughput screening capabilities using several plates with 100 plants each, though it is substantially smaller than the other two use cases.We could upload all this data to a dedicated server if you can provide one, but the replication effort may be prohibitive regardless. These use cases were conceived as comprehensive demonstrations of the platform's capabilities rather than as easily reproducible examples.However, I recognize that these computational requirements create significant barriers for reproducibility verification. To address this challenge, I propose implementing a revision of both the manuscript and the GitHub repository that introduces simplified demonstration use cases for a small subset of plants that can be completely reproducible. These would feature single time series with 4-6 plants for root architecture analysis and two sets of experiments for hypocotyl and germination analysis for screening demonstrations, enabling reproduction within hours rather than weeks.While this represents substantial manuscript modifications from our side, I believe it would significantly enhance the accessibility and impact of ChronoRoot 2.0 for the broader plant biology community. We are fully committed to undertaking these changes to ensure our work meets the highest reproducibility standards and serves as a practical resource for researchers.Would you be open to considering this revision? I am prepared to implement these changes promptly and provide the simplified demo datasets for immediate review.[Email sent to the authors #2]You mentioned that limited computational

resources would limit the reproducibility of the study. Therefore, we have not started our review yet.Revising your paper with simplified demonstration is an good idea. We will be glad to reproduce the results of this demo once you share it with us.We will do our best to complete this as quickly as possible so as not to delay the process.[Response received from the authors #2]Thank you for your patience regarding the reproducibility concerns.I have now implemented the simplified demonstration approach we discussed to address the computational resource limitations.I have updated the GitHub repository (https://github.com/ngaggion/ChronoRoot2/tree/master) to include PDF tutorials covering all interface functionalities and uploaded three lightweight demo datasets to the Docker image.Each dataset contains one video and is designed to test our three core analysis capabilities: plant root architecture analysis, germination analysis, and hypocotyl under etiolation analysis.To access these changes, please pull the updated Docker image again and the new materials will be available.The results obtained by following the tutorials are already stored along with the segmentation outputs generated by running the models.To re-run the segmentation script, you can simply use the "Delete Seg" button from the interface to re-run the analysis, otherwise this saves considerable processing time.Please let me know if you encounter any issues with the tutorials or need any additional clarification.-> Reproduce demo FPCAWe continue to reproduce the demo dataset results using the pre-trained model provided by the authors. The following command pulls the Docker image and launches the Graphical User Interface (GUI) inside the Docker environment used for the reproducibility analysis. The GUI is launched with XQuartz v. 2.8.5.docker pull ngaggion/chronoroot:latestxhost + 127.0.0.1docker run --rm -it \--platform=linux/amd64 \-e DISPLAY=host.docker.internal:0 \-e QT_X11_NO_MITSHM=1 \-e LIBGL_ALWAYS_INDIRECT=1 \-v "$PWD/chronoroot_out:/out" \--name chronoroot \ngaggion/chronoroot:latestchronorootThe remaining steps are according to the screening interface tutorial provided by the authors of the manuscript. In this review we focus on reproducing the plant root architecture analysis.From the window on the right, analyze plant is clicked for plant 1-4 and identifier sequence A-B-A-B, following the tutorial.Thereafter, the process all plants button was clicked. ROI was chosen to cover each plant; seed placement was selected to align with the seed's location at Day 0The images below illustrate ROI selection as well as seed placement (blue dot) for plant 3 at day 0 and day 10 (see screenshot).The images below show the analysis overview (see screenshot). The first image shows the authors' results and the second image shows the reproduced results. The error rates differ for plant 1,3 and 4 but align for plant 2, likely due to differences in ROI selection and seed placement.Authors' results: (see screenshot)Reproduced results: (see screenshot)The image below (see screenshot) shows the settings used to generate the final report including the main FPCA result figures.5.3 Statistical comparison Original vs Reproduced results- Results: The figures below show the reproduced FPCA result on the demo data set as well as the result provided by the authors of the manuscript. Note that these images show results on the demo data and not on the experimental data analyzed in the manuscript. As can be seen the reproduced results do not match the results reported. The reproduced Mann-Whitney test p-value was 0.67 (original 0.33) for main root length and 0.41 (original 0.67) for lateral root length.See screenshot of Reproduced FPCA result on the demo dataset (corresponding to Fig. 4B in the manuscript).See screenshot of Original FPCA result on the demo dataset (corresponding to Fig. 4B in the manuscript).See screenshot of Reproduced FPCA result on the demo dataset (corresponding to Fig. 4D in the manuscript).See screenshot of Original FPCA result on the demo dataset (corresponding to Fig. 4D in the manuscript).- Comments: The mismatches were likely due to the

ROI selection and seed placement which was done manually. The rationale and examples of selection and placement used in this review is contained in section 5.2 for transparency. As was shown in section 5.2, the error rates were aligned for plant 2 but not for the remaining plants, indicating differences in ROI selection and seed placement.- Errors detected: In the manuscript, the section "Use Case 1" refers to the figure 5 instead of the figure 4.- Statistical Consistency: The FPCA results were not consistent.---6. Conclusion- Summary of the computational reproducibility reviewThe main result of the paper was not reproduced since the original data required more computational resources than was available for this review. The reproducibility review was instead performed on a demo dataset supplied by the authors. The reproduced result on the demo data set differed from the author's results. A likely reason for the mismatch is differences in the manual ROI selection and seed placement - which the authors point out are crucial for the end result.- Recommendations for authorsWe recommend the following improvements based on our observations to enhance the study's reproducibility:-- ROI selection and seed placement: The results depend heavily on ROI selection and seed placement. However, the tutorial did not mention how to choose this to reproduce the end result. Adding this would improve reproducibility.-- MacOS installation guide: An installation guide is provided for Linux and Windows. Adding a guide for MacOS users would enhance reproducibility.-- p-value reporting: The p-value reported in Figs. 4B and 4D is currently only through an asterisk, indicating significance. Reporting exact values improves reproducibility.

Level of Interest

Please indicate how interesting you found the manuscript: Choose an item.

Quality of Written English

Please indicate the quality of language in the manuscript: Choose an item.

Declaration of Competing Interests

Please complete a declaration of competing interests, considering the following questions:

- Have you in the past five years received reimbursements, fees, funding, or salary from an organisation that may in any way gain or lose financially from the publication of this manuscript, either now or in the future?
- Do you hold any stocks or shares in an organisation that may in any way gain or lose financially from the publication of this manuscript, either now or in the future?
- Do you hold or are you currently applying for any patents relating to the content of the manuscript?
- Have you received reimbursements, fees, funding, or salary from an organization that holds or has applied for patents relating to the content of the manuscript?
- Do you have any other financial competing interests?
- Do you have any non-financial competing interests in relation to this paper?

If you can answer no to all of the above, write 'I declare that I have no competing interests' below. If your reply is yes to any, please give details below.

1. No 2. No 3. No 4. No 5. No 6. No

I agree to the open peer review policy of the journal. I understand that my name will be included on my report to the authors and, if the manuscript is accepted for publication, my named report including any attachments I upload will be posted on the website along with the authors' responses. I agree for my report to be made available under an Open Access Creative Commons CC-BY license (http://creativecommons.org/licenses/by/4.0/). I understand that any comments which I do not wish to be included in my named report can be included as confidential comments to the editors, which will not be published.

Choose an item.

To further support our reviewers, we have joined with Publons, where you can gain additional credit to further highlight your hard work (see: https://publons.com/journal/530/gigascience). On publication of this paper, your review will be automatically added to Publons, you can then choose whether or not to claim your Publons credit. I understand this statement.

Yes Choose an item.
