# Supplementary material for: ChronoRoot 2.0: an open AI-powered platform for 2D temporal plant phenotyping
Source: Gigascience. 2026 Feb 28;15:giag018. doi: 10.1093/gigascience/giag018 (PMC13042295; doi:10.1093/gigascience/giag018)
Supplement: giag018_Reviewer_4_Report_revision_1 — Reviewer 4 -- 2/4/2026 [file giag018_reviewer_4_report_revision_1.pdf]

Reviewer Report

Title: ChronoRoot 2.0: An Open AI-Powered Platform for 2D Temporal Plant Phenotyping

Version: Revision 1 **Date:** 2/4/2026

Reviewer name: Reviewer 4

Reviewer Comments to Author:

Reproducibility report for: ChronoRoot 2.0: An Open AI-Powered Platform for 2D Temporal Plant PhenotypingJournal: GigaScienceID number/DOI: GIGA-D-25-00276Reviewer(s): Tobias Waÿngberg, Department of Clinical Neuroscience, Karolinska Institutet, Sweden [Worked on reproducing the results and contributed to writing the report.]Laura Caquelin, Department of Clinical Neuroscience, Karolinska Institutet, Sweden [Reviewed the final report.]---1. ContextThis report corresponds to a second assessment of the computational reproducibility of the article GIGA-D-25-00276, following a revision by the authors after the first round of review.The scope of the computational reproducibility review is to verify that ChronoRoot 2.0 can reproduce the root-growth analysis workflow for Use Case 1 - from time-lapse images through ROI selection and root-start initialization to exported growth time-series and functional PCA outputs and associated report statistics - using the authors' provided Docker image, tutorials, and demo datasets.---2. Changes since the first reviewThe authors addressed our prior recommendations by:(1) Expanding the Standard Interface documentation to explain that the "seed" defines the spatiotemporal origin of main-root growth and should be placed at the frame where the main root first emerges, with added examples for Arabidopsis and tomato via a new documentation website;(2) Adding a brief macOS section to their Docker tutorial (XQuartz-based GUI setup) while noting macOS support is still limited;(3) And clarifying that while figures retain star-based significance notation, ChronoRoot 2.0 exports accompanying text files containing exact p-values and descriptive statistics for all reported parameters, and they standardized the star thresholds across figures.---3. Availability of Materialsa. Data- Data availability:Experimental time-lapse sequences: not open.Annotated training/validation set: open.Demo datasets: open.- Data completeness:Experimental time-lapse sequences: Incomplete.Annotated training/validation set: complete.Demo datasets: complete.- Access Method:Experimental time-lapse sequences: NA.Annotated training/validation set: Huggingface.Demo datasets: Docker image-Repository:Experimental time-lapse sequences: NA.Annotated training/validation: https://huggingface.co/datasets/ngaggion/ChronoRoot_nnUNet.Demo datasets: https://hub.docker.com/r/ngaggion/chronoroot.-Data quality: The data is structured and clear for the training data set and the demo datasets. The experimental time-lapse data used for evaluating the software was not shared due to computational limitations (see Section 5.2 in the first round report for details).b. Code- Code availability: Open- Programming Language(s): Python- Repository link: https://github.com/ChronoRoot/ChronoRoot2.- License: GNU General Public License v3.0.- Repository status: Public- Documentation: The repository comes with clear documentation.---4. Computational environment of reproduction analysis-

Operating system for reproduction: MacOS Tahoe version 26.2 (Ubuntu 22.04.4 LTS inside the Docker image).- Programming Language(s): Python.- Code implementation approach: Using shared code.- Version environment for reproduction: Python 3.8.2 (inside docker image).---5. Results5.1 Original study results- Results 1: Mann-Whitney tests on PC scores for main and lateral root length for the demo A vs B grouping.5.2 Steps for reproduction-> Obtain code and launch ChronoRoot 2.0 via DockerThe GUI was launched by running the following terminal commands.git clone https://github.com/ChronoRoot/ChronoRoot2.gitcd ChronoRoot2mkdir -p chronoroot_outxhost + 127.0.0.1docker pull --platform=linux/amd64 ngaggion/chronoroot:latestdocker run --rm -it \--platform=linux/amd64 \-e DISPLAY=host.docker.internal:0 \-e QT_X11_NO_MITSHM=1 \-e LIBGL_ALWAYS_INDIRECT=1 \-v "$PWD/chronoroot_out:/out" \--name chronoroot \ngaggion/chronoroot:latest \bashchronoroot-> Open the Standard Root Phenotyping Interface and select demo dataIn the GUI, open the Standard Interface, set Project folder to /out/… (mounted output directory), and set Video folder to the demo root dataset under /Demo/rpi101_roots. A screenshot of these settings is provided in standard_interface.png (attached as supplementary material).-> Define ROIs and main root start points (manual initialization)For each of the four plants, Analyze Plant was used to (i) draw and confirm a plant-specific ROI and (ii) select the main root start point by advancing the timeline to the first frame where the main root is clearly segmented (around Day 2) and clicking the top of the main root at the root-shoot junction to remove segmentation above the point. Screenshots documenting the manual initialization are provided as ROI1.png-ROI4.png and seed1.png-seed4.png, where the index corresponds to plant numbers 1-4 (left-to-right on the plate).-> Process plants and generate reportAfter ROI and main root start point initialization for plants 1-4, Process all plants was executed. Once all plants finished processing, the Generate Report tab was used to produce the Standard Interface report (including FPCA outputs and group-comparison statistics as configured in the GUI). A screenshot of the report-generation settings in the GUI is provided in Report_settings.png attached with this report.5.3 Statistical comparison Original vs Reproduced results- Reproduced results: ChronoRoot 2.0 exported Mann-Whitney U test statistics for FPCA component scores (PC1/PC2) in MainRootLength_stats.txt and LateralRootLength_stats.txt.In our reproduction, the reported p-values were:-- MainRootLength PC1 p = 0.6667, PC2 p = 1.0;-- LateralRootLength PC1 p = 0.6667, PC2 p = 0.6667.In the author-provided demo outputs, the reported p-values were:-- MainRootLength PC1 p = 1.0, PC2 p = 0.3333;-- LateralRootLength PC1 p = 1.0, PC2 p = 0.6667.- Comments: In both the author-provided and reproduced demo outputs, the qualitative conclusion is the same: experiments A and B are reported as not significantly different for PC1 and PC2 for both traits. Exact numerical agreement was partial (only LateralRootLength PC2 matched exactly). In addition, the p-values obtained in the current reproduction differ from those obtained in the previous review round (previous round: MainRootLength PC1 p = 1.0, PC2 p = 0.6667; LateralRootLength PC1 p = 1.0, PC2 p = 0.4142), indicating variability across runs.- Errors detected: There were discrepancies in exact p-values across (i) author-provided demo outputs, (ii) the current reproduction, and (iii) the previous review round suggesting remaining sensitivity to manual initialization (ROI selection and main-root start-point placement) and/or software/version differences, despite more detailed instructions provided by the authors.- Statistical Consistency: The statistical method was consistent (Mann-Whitney U test on FPCA component scores comparing A vs B). The inference (non-significant differences) was consistent across outputs, but exact p-values were not stable across reproductions on the demo dataset.---

--6. Conclusion- Summary of the second computational reproducibility reviewIn this second round, we verified the authors' updates to the documentation and Docker distribution and re-ran the Standard Interface workflow on the provided demo dataset. The pipeline was reproducible in the sense that:(1) The software could be launched on macOS via Docker/XQuartz,(2) The manual initialization (ROI + main-root start point) and plant processing could be completed,(3) And the report generation produced FPCA outputs and exported text files with exact Mann-Whitney U p-values for PC1/PC2.However, exact numerical agreement with the author-provided demo p-values was only partially reproduced: while the qualitative inference (A vs B non-significant for both main and lateral root FPCA components) matched, several exact p-values differed across the author-provided demo outputs, our current reproduction, and the previous review round, indicating remaining sensitivity/variability in the demo results.- Follow-up on previous recommendations:The authors have substantially addressed the main reproducibility comments:(1) ROI/seed placement: They clarified that the "seed" defines the spatiotemporal origin of growth and added detailed examples explaining that the point should be placed at the frame where the main root first emerges; this targets the previously identified source of variation.(2) MacOS guide: They added a macOS section to the Docker tutorial, though support remains less mature than for Linux/Windows.(3) P-value reporting: While figures still use asterisk notation, the software exports text files with exact p-values (and the manuscript now clarifies this). Further improvements that could strengthen exact reproducibility include automatically exporting the chosen ROI coordinates and main-root start point as metadata to reduce remaining ambiguity and run-to-run variability.

Level of Interest

Please indicate how interesting you found the manuscript: Choose an item.

Quality of Written English

Please indicate the quality of language in the manuscript: Choose an item.

Declaration of Competing Interests

Please complete a declaration of competing interests, considering the following questions:

- Have you in the past five years received reimbursements, fees, funding, or salary from an organisation that may in any way gain or lose financially from the publication of this manuscript, either now or in the future?
- Do you hold any stocks or shares in an organisation that may in any way gain or lose financially from the publication of this manuscript, either now or in the future?
- Do you hold or are you currently applying for any patents relating to the content of the manuscript?
- Have you received reimbursements, fees, funding, or salary from an organization that holds or has applied for patents relating to the content of the manuscript?

- Do you have any other financial competing interests?
- Do you have any non-financial competing interests in relation to this paper?

If you can answer no to all of the above, write 'I declare that I have no competing interests' below. If your reply is yes to any, please give details below.

1. no 2. no 3. no 4. no 5. no 6. no

I agree to the open peer review policy of the journal. I understand that my name will be included on my report to the authors and, if the manuscript is accepted for publication, my named report including any attachments I upload will be posted on the website along with the authors' responses. I agree for my report to be made available under an Open Access Creative Commons CC-BY license (http://creativecommons.org/licenses/by/4.0/). I understand that any comments which I do not wish to be included in my named report can be included as confidential comments to the editors, which will not be published.

Choose an item.

To further support our reviewers, we have joined with Publons, where you can gain additional credit to further highlight your hard work (see: https://publons.com/journal/530/gigascience). On publication of this paper, your review will be automatically added to Publons, you can then choose whether or not to claim your Publons credit. I understand this statement.

Yes Choose an item.
